# Thermodynamic Stability and Speciation of Ga(III) and Zr(IV) Complexes with High-Denticity Hydroxamate Chelators

**DOI:** 10.1021/acs.inorgchem.1c01622

**Published:** 2021-08-20

**Authors:** Yuliya Toporivska, Andrzej Mular, Karolina Piasta, Małgorzata Ostrowska, Davide Illuminati, Andrea Baldi, Valentina Albanese, Salvatore Pacifico, Igor O. Fritsky, Maurizio Remelli, Remo Guerrini, Elzbieta Gumienna-Kontecka

**Affiliations:** † University of Wroclaw, Faculty of Chemistry, 14 F. Joliot-Curie, 50-383 Wrocław, Poland; ‡ University of Ferrara, Dipartimento di Scienze Chimiche, Farmaceutiche ed Agrarie, 46 Via Luigi Borsari, 44121 Ferrara, Italy; § Taras Shevchenko National University of Kyiv, Department of Chemistry, 64 Volodymyrska Str., 01601 Kyiv, Ukraine

## Abstract

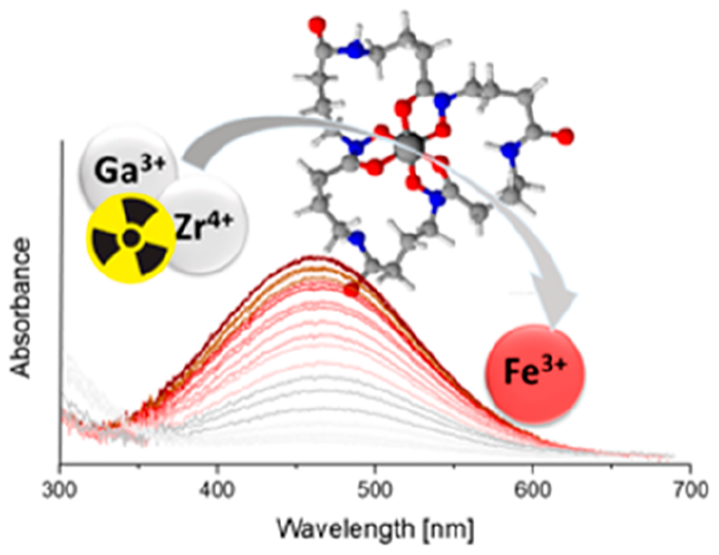

Increasing attention has been recently devoted to ^89^Zr(IV) and ^68^Ga(III) radionuclides, due to their favorable decay characteristics for positron emission tomography (PET). In the present paper, a deep investigation is presented on Ga(III) and Zr(IV) complexes with a series of tri-(**H**_**3**_**L1**, **H**_**3**_**L3**, **H**_**3**_**L4** and desferrioxamine E, **DFOE**) and tetrahydroxamate (**H**_**4**_**L2**) ligands. Herein, we describe the rational design and synthesis of two cyclic complexing agents (**H**_**3**_**L1** and **H**_**4**_**L2**) bearing three and four hydroxamate chelating groups, respectively. The ligand structures allow us to take advantage of the macrocyclic effect; the **H**_**4**_**L2** chelator contains an additional side amino group available for a possible further conjugation with a biomolecule. The thermodynamic stability of Ga(III) and Zr(IV) complexes in solution has been measured using a combination of potentiometric and pH-dependent UV–vis titrations, on the basis of metal–metal competition. The Zr(IV)-**H**_**4**_**L2** complex is characterized by one of the highest formation constants reported to date for a tetrahydroxamate zirconium chelate (log β = 45.9, pZr = 37.0), although the complex-stability increase derived from the introduction of the fourth hydroxamate binding unit is lower than that predicted by theoretical calculations. Solution studies on Ga(III) complexes revealed that **H**_**3**_**L1** and **H**_**4**_**L2** are stronger chelators in comparison to DFOB. The complex stability obtained with the new ligands is also compared with that previously reported for other hydroxamate ligands. In addition to increasing the library of the thermodynamic stability data of Ga(III) and Zr(IV) complexes, the present work allows new insights into Ga(III) and Zr(IV) coordination chemistry and thermodynamics and broadens the selection of available chelators for ^68^Ga(III) and ^89^Zr(IV).

## Abstract 2

Solution equilibria studies on Ga(III) and Zr(IV) complexes with a series of tri- and tetrahydroxamate ligands are presented. For this purpose, the rational design and synthesis of two cyclic complexing agents bearing three and four hydroxamate chelating groups was performed. The thermodynamic and speciation studies allow a discussion of the structure−complex stability dependence. The Zr(IV)-tetrahydroxamate complex is characterized by one of the highest formation constants reported to date for a hydroxamate zirconium chelator.

## Introduction

Recent research confirms an increasing interest in the use of gallium and zirconium radioiosotopes for medical diagnostic techniques such as PET or single-photon emission computed tomography (SPECT).^1−8^

The interest in the use of ^68^Ga (*t*_1/2_ = 1.13 h, *E*_β+avg_ = 830 keV, 89%) for clinical PET comes from the accessibility of its production via an easily portable and long-lived ^68^Ge/^68^Ga generator system.^9^ The favorable decay characteristics of ^89^Zr (*t*_1/2_ = 78.4 h, *E*_β+avg_ = 395.5 keV, 23%) allow high PET image resolution to be obtained, since the sufficiently long half-life is an optimal match for the pharmacokinetics of most monoclonal antibodies.^10^

To be applied to molecular imaging, the metal isotope must be converted into a radiolabeled probe that can specifically reach the target of interest *in vivo* and remain there long enough to be detected. Therefore, the metal ion must be bound by an efficient chelator to overcome metal hydrolysis and transchelation, and linked to a biologically active targeting molecule, to be properly directed to the desirable molecular target *in vivo*.

To the best of our knowledge, the most widely used chelator for ^68^Ga is 1,4,7,10-tetraazacyclododecane-1,4,7,10-tetraacetic acid (DOTA).^11^ In 2016 the FDA approved a ^68^Ga-DOTATATE (NETSPOT) kit for the preparation of a ^68^Ga-DOTATATE injection;^12^ clinical trials revealed the superiority of ^68^Ga-DOTATATE with respect to ^111^In-pentetreotide in imaging neuroendocrine tumors.^13^ Currently, the most successfully used ^89^Zr(IV) chelator is DFOB, but some decomposition has been observed over time *in vivo*, and ^89^Zr slowly accumulates in bones.^14−16^

Many chelators have been already suggested for ^68^Ga and ^89^Zr on the basis of *in vivo* assays.^14,17−23^ While scientists are currently devoting considerable efforts toward the design of more efficient ^89^Zr and ^68^Ga chelators by increasing the *in vivo* stability of the corresponding complexes, the solution equilibrium chemistry, especially of Zr(IV) systems, has still rarely been investigated.^2,24−27^ Solution studies on the coordination chemistry and formation constants of Zr(IV) complexes are not trivial for several reasons: an extremely high thermodynamic stability (requiring application of indirect competition methods with the use of other strong ligands with known stability constants), strong hydrolysis (occurring over almost the entire pH range), and the lack of spectral activity of the complexes. On the other hand, knowledge of the speciation of such complexes, especially at physiological pH, could provide information concerning the actual chemical form of the complex in biological media, and this can contribute to both a better understanding of the *in vivo* speciation and an explanation of the differences in the biological activity.

Our laboratory has recently reported the thermodynamic properties of Zr(IV)-DFOB complexes, suggesting the formation of three mononuclear complexes, i.e. [ZrHL]^2+^ [ZrL]^+^, and [ZrLH_–1_], over the pH range 1–11.^24^ The stability constants and pZr value determined for the Zr(IV)-DFOB system place DFOB among the best Zr(IV) chelators, although the formation of six-coordinate unsaturated complexes (i.e., with the coordination sphere of Zr(IV) completed by two water molecules^28^ or hydroxide ligands^29^) and the susceptibility of coordinated water molecules to deprotonation were suggested to be the reason for the *in vivo* lability of ^89^Zr(IV)-DFOB complexes. The thermodynamic stability of Zr(IV)-DFOB complexes is in line with *in vivo* research^15,16,30,31^ and also with Holland’s recent DFT calculations,^32^ indicating that our experimental approach was appropriate.

**Scheme 1:**
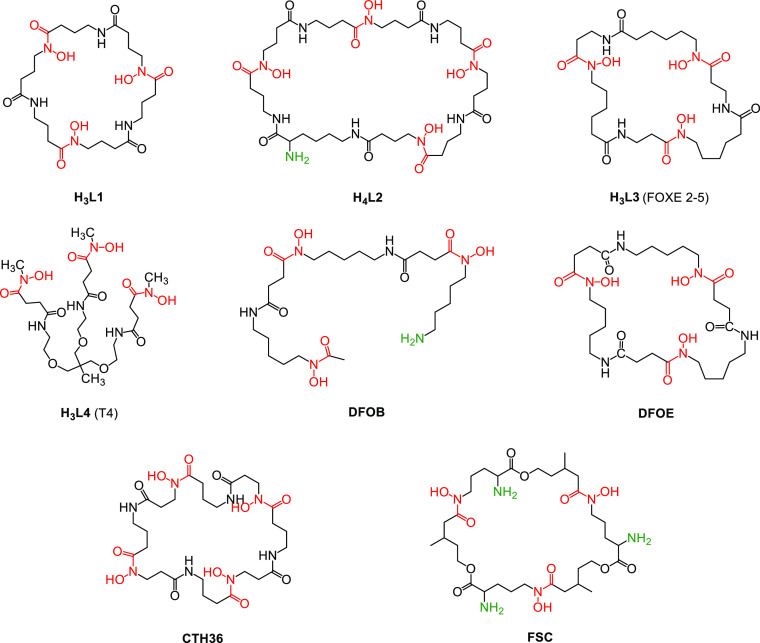
Structures of the Ligands Investigated and Discussed in This Paper

By capitalizing upon our earlier works on siderophore mimics,^33−36^ in this work we have designed, synthesized, and fully characterized tri- and tetrahydroxamic **H**_**3**_**L1** and **H**_**4**_**L2** chelators (Scheme 1), analogues of **DFOE**,^37^ and their Ga(III) and Zr(IV) complexes. In addition, the physicochemical properties of Zr(IV) complexes with three further trihydroxamic ligands, i.e. **H**_**3**_**L3** (FOXE 2-5),^38^**H**_**3**_**L4** (T4),^35^ and **DFOE** (Scheme 1), were also investigated for the sake of comparison. The ligands employed here were selected to investigate the influence of some structural elements on the physicochemical properties of Zr(IV) complexes: i.e., (i) the number of binding groups required to complete the coordination sphere of the metal cation; (ii) the possible advantage of the macrocyclic effect; (iii) the size of the ligand cavity, which should be large enough to minimize ring strain; (iv) the symmetry of the ligand, which could limit the probability of a complex challenge. Ga(III) (and Fe(III)) binding to all of these ligands was also performed and will be discussed here, as the relation between the ligand structure and the stability of the complexes has been much more studied and is better understood for trivalent metal ions. Moreover, Fe(III) complexes are used as a tool to determine the thermodynamic stability of Ga(III) and Zr(IV) analogues.

According to DFT studies, to minimize the ring strain, the cyclic tetrahydroxamic ligand should consist of a minimum of 36 atoms, which gives at least 7 chemical groups or 8 bonds.^39,40^ The tetrahydroxamic ligand **H**_**4**_**L2**, designed to completely saturate the oxophilic coordination sphere of Zr(IV), meets this criterion, possessing at least 9 bonds between binding groups. Of note, **H**_**4**_**L2** has been designed with the aim of introducing in the chelator a primary amine group, useful to improve the solubility of the ligand but, primarily, to allow the easy conjugation of the chelator to a targeting molecule for future *in vivo* studies. Trihydroxamic **H**_**3**_**L1** is a symmetrical, macrocyclic ligand, comprising 9 bonds between binding groups; it allows the determination and direct comparison of the influence of the fourth binding group of **H**_**4**_**L2** on Zr(IV) stability. This ligand also reveals the effect of the shortening of the chain in comparison to **DFOE**, **H**_**3**_**L3**, and DFOB, all with 10 bonds (Scheme 1). Tripodal **H**_**3**_**L4**, used earlier as a good mimic of a ferrichrome siderophore, was investigated here in order to compare its Zr(IV) binding capacity to those of other tri- and tetrahydroxamic ligands.^35^ In **H**_**3**_**L1**, **H**_**4**_**L2**, and **H**_**3**_**L3**(38) we have used retrohydroxamic units, with a reversed order with respect to the native hydroxamic moiety.

Until now, the stability constants for tetrahydroxamate Zr(IV) complexes have only been estimated from computational calculations^32^ for linear DFO* (log β_[Zr(DFO*)]_ = 51.56) and cyclic CTH36 (log β_[Zr(CTH36)]_ = 52.84). DFO*^41^ and CTH36^39^ possess 10 and 8 bonds between two hydroxamic groups, respectively.

Therefore, an experimental verification of the order of the stability increase between tri- and tetrahydroxamic chelators should be very useful. The same is true for examining the effects of a number of other variable structural elements, such as the symmetry of the structure and the length of the chain between the hydroxamate binding units. Although the thermodynamic formation constants do not predict *in vivo* stability and kinetic inertness, they are very useful and interesting parameters that may allow for a better design of efficient chelators for Zr(IV) ions.

## Results and Discussion

### Design and Synthesis of the Ligands

**Scheme 2:**
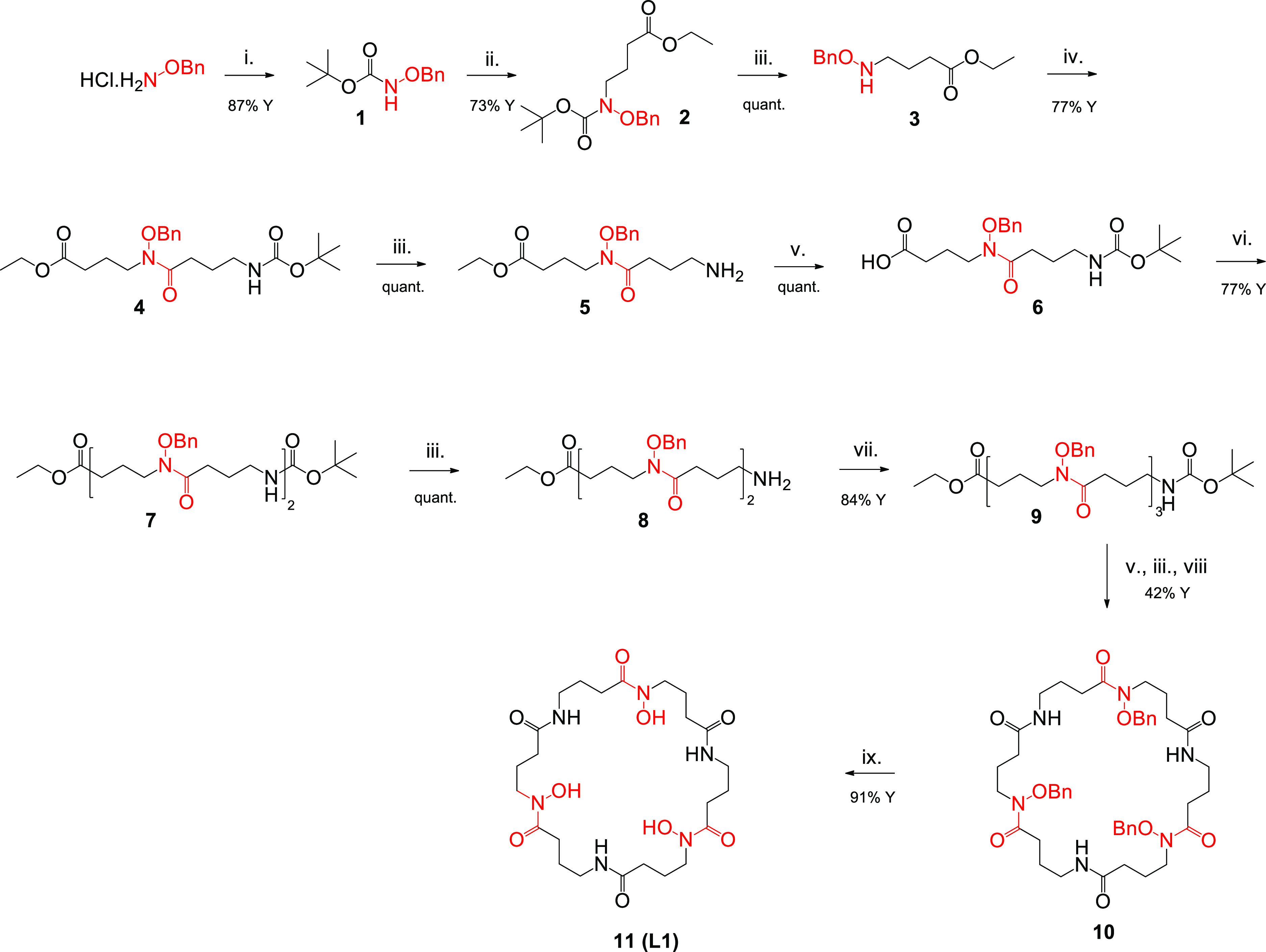
Synthetic Pathway of the Ligand **H**_**3**_**L1** Reagents and conditions: (i) (Boc)_2_O, K_2_CO_3_, H_2_O/dioxane; (ii) ethyl 4-bromobutyrate, NaH, DMF; (iii) TFA; (iv) Boc-γ-aminobutyric acid, HATU, DIPEA, DMF; (v) LiOH, H_2_O/dioxane; (vi) **5**, HATU, DIPEA, DMF; (vii) **6**, HATU, DIPEA, DMF; (viii) HATU, DIPEA, DMF; (ix) H_2_, Pd/C, MeOH.

**Scheme 3:**
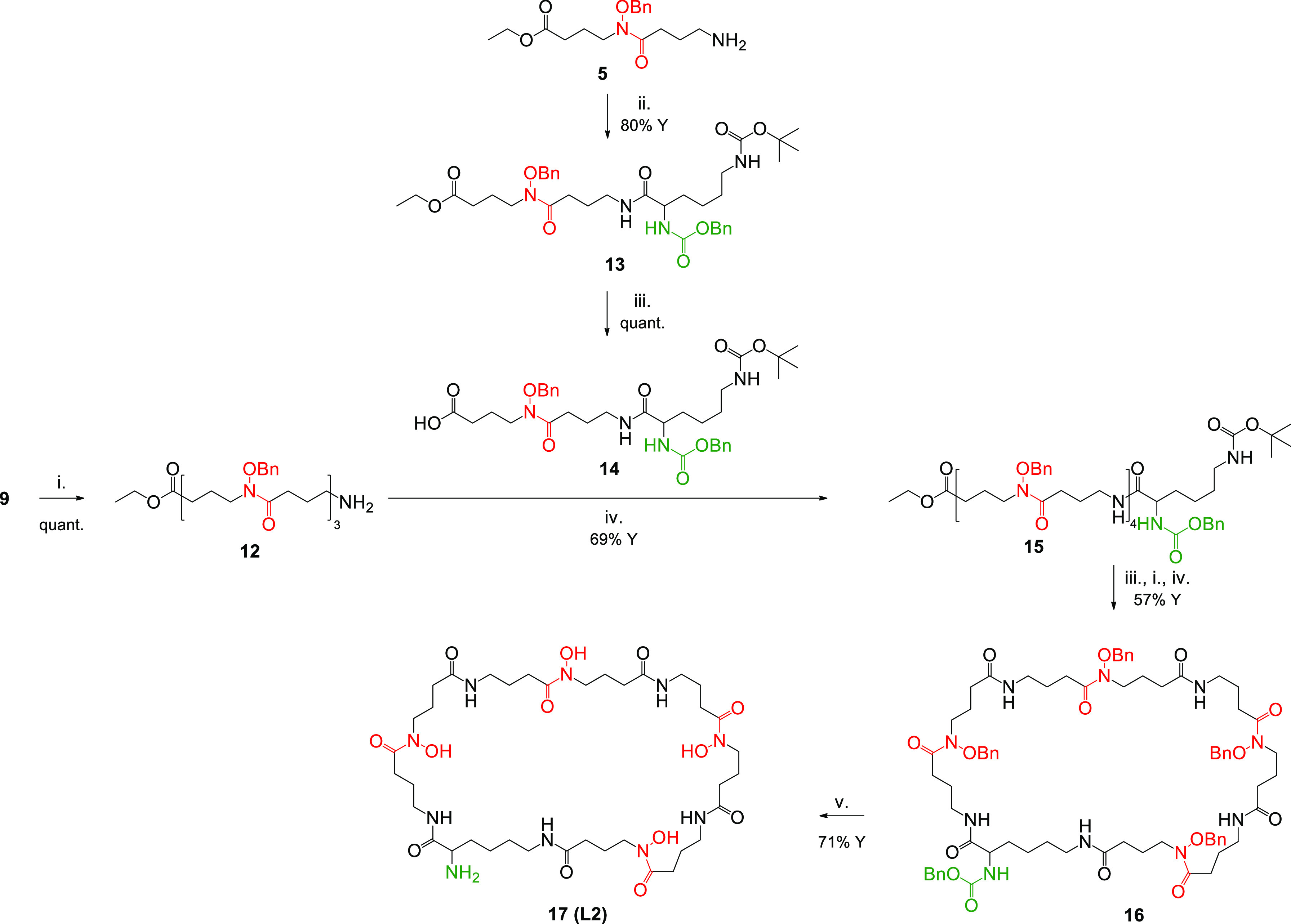
Synthetic Pathway of the Ligand **H**_**4**_**L2** Reagents and conditions: (i) TFA; (ii) Z-Lys(Boc)-OH, HATU, DIPEA, DMF; (iii) LiOH, H_2_O/dioxane; (iv) HATU, DIPEA, DMF; (v) H_2_, Pd/C, MeOH.

The synthetic approaches for the preparation of **H**_**3**_**L1** and **H**_**4**_**L2** are depicted in Schemes 2 and 3, respectively. The hydroxamate-based monomers protected either at the carboxylic group as an ethyl ester (**5**) or at the amino function with Boc (**6**) were employed as common synthetic precursors of **H**_**3**_**L1** and **H**_**4**_**L2**. The building blocks **5** and **6** were synthesized by starting from *O*-benzylhydroxylamine hydrochloride that was first reacted with di-*tert*-butyl dicarbonate to give compound **1** and then alkylated with ethyl 4-bromobutyrate in the presence of NaH to provide compound **2** (Scheme 2). Boc removal with TFA furnished the intermediate **3**, which was coupled with Boc-protected γ-aminobutyric acid using HATU as a coupling reagent. This allowed us to obtain the orthogonally protected **4** as a suitable precursor of both **5** and **6**, which were alternatively isolated after acidic and basic treatments, respectively.

For the synthesis of **H**_**3**_**L1**, **5** and **6** were linked together via a standard amide coupling followed by TFA treatment to give **8**. The reaction with another unit of **6** gave **9** as the linear protected precursor of **H**_**3**_**L1**. A head–tail HATU-mediated cyclization of **9** was realized under dilute conditions (0.5 mg/mL) after removal of the protection at the C and N terminal positions. A final Pd-catalyzed hydrogenolysis afforded the desired macrocycle **H**_**3**_**L1** in good yields.

For the synthesis of the tetrahydroxamic derivative **H**_**4**_**L2** (Scheme 3), an appropriate hydroxamate-bearing lysine derivative was first prepared as a building block (**14**). This was obtained by a coupling reaction between **5** and a residue of Z-Lys(Boc)-OH followed by saponification of the ester function. The monomeric unit **14** was coupled to **12**, which resulted from TFA-mediated Boc deprotection of **9**. The resulting intermediate **15** underwent head–tail HATU-mediated cyclization after removal of the protections at the C and N terminal positions as described above, leading to **16**. In addition in this case, the final macrocycle was obtained after Pd-catalyzed removal of the benzyl functions from the hydroxamic groups. These conditions led also to CBz cleavage, leaving the free amino group suitable for future bioconjugation strategies.

**H**_**3**_**L1**, **H**_**4**_**L2**, and their precursors were fully characterized by ^1^H and ^13^C NMR (see the Supporting Information). The degree of purity of the final product was evaluated by analytical HPLC assays (see the Supporting Information), showing a purity of higher than 95%.

### Thermodynamic Solution Studies

#### Ligand Protonation Constants

The metal affinity of a ligand depends on its acid–base properties; therefore, the protonation equilibria of **H**_**3**_**L1** and **H**_**4**_**L2** were first investigated. The proton-dissociation processes of the ligands were followed by potentiometric titrations in the pH range 2–11. The hydrolytic stability of the ligands was monitored by a second titration of the same sample with NaOH, following back-acidification of the initially titrated sample. The recorded titration curves were almost exactly superimposed; consequently, the protonation constants calculated from the two consecutive titrations were found to be equal within 0.05 log unit, which indicated that no decomposition occurred.

Data analysis allowed the determination of three protonation constants for **H**_**3**_**L1** and four protonation constants for **H**_**4**_**L2**; all of them fall in the pH range 8–10 and can be attributed to the hydroxamate groups (Table 1). For each ligand, the protonation constants were assigned by comparing them with the known protonation constants of hydroxamate ligands.^24,35,37,42^ When the changes in temperature, ionic strength, and ligand structures are allowed for, the protonation constants of **H**_**3**_**L1** and **H**_**4**_**L2** are in excellent agreement with the literature values of the cyclic hydroxamate siderophore **DFOE** (log *K*_1_ = 9.89, log *K*_2_ = 9.42, and log *K*_3_ = 8.65) reported by Anderegg et al.^37^ The pH-dependent UV–vis titrations of **H**_**3**_**L1** and **H**_**4**_**L2** (Figure S1) revealed the development of a strong band with λ_max_ = 230 nm, when the pH was increased from 7 to 11, which is usually observed for a hydroxamic group deprotonation process.^24,43^ The amino group protonation of **H**_**4**_**L2** was not detectable in the experimental pH range. We are aware that the electron-withdrawing character of both the −NH_3_^+^ and −NHOH groups affects the acidity of the other group in comparison with that in the related nonsubstituted compound **H**_**3**_**L1**. As was previously reported in the example of α- and β-alanine hydroxamic acids, the amino group may be more acidic than the hydroxamic group, or vice versa. According to the literature, the amino group may be more acidic than hydroxamic one, or vice versa.^44,45^ On the basis of the protonation constant of the amino group of DFOB (log *K*_amine_ = 10.97^24^), we assume for **H**_**4**_**L2** that it is >11. However, we keep in mind that the deprotonation processes of amino and hydroxamate groups overlap and cannot be distinguished by potentiometry. To elucidate the protonation microequilibria of **H**_**4**_**L2**, ^1^H NMR titrations should be carried out. However, such a precise analysis is not needed for the determination of the stability of **H**_**4**_**L2**-metal complexes and therefore was not performed. The species distribution diagrams of **H**_**3**_**L1** and **H**_**4**_**L2** are presented in Figure S2. The protonation constants of **H**_**3**_**L3** and **H**_**3**_**L4** were reported elsewhere (Table 1).^35,38^

**Table 1:** Protonation Constants of Ligands and log β Values of Complexes Formed with Fe(III), Ga(III), and Zr(IV)^a^ Ions

	**H**_**3**_**L1**		**H**_**4**_**L2**		**H**_**3**_**L3** (FOXE 2–5)^38^^,^^b^		**H**_**3**_**L4 (T4)**(35)^,^^c^		**DFOE**(36,38)^,^^b^	
assignt	log β	log *K*	log β	log *K*	log β	log *K*	log β	log *K*	log β	log *K*
LH	9.89(1)^d^	9.89^d^	10.06(1)^d^	10.06^d^	9.89	9.89	9.50	9.50	9.89	9.89
LH_2_	19.13(1)^d^	9.24^d^	19.65(1)^d^	9.59^d^	19.31	19.31	18.47	8.97	19.31	9.42
LH_3_	27.44(1)^d^	8.31^d^	28.59(1)^d^	8.94^d^	27.96	27.96	26.73	8.26	27.96	8.65
LH_4_			36.77(1)^d^	8.18^d^						

	**H**_**3**_**L1**		**H**_**4**_**L2**		**H**_**3**_**L3** (FOXE 2–5)^38^^,^^b^		**H**_**3**_**L4 (T4)**(35)^,^^c^		**DFOE**(36,38)^,^^b^	
	log β	p*K*_a_	log β	p*K*_a_	log β	p*K*_a_	log β	p*K*_a_	log β	p*K*_a_
[FeH_2_L]			39.96(3)^e^	3.00^d^						
				3.12^d^						
[FeHL]	31.80(3)^e^	3.21^d^	36.96(7)^e^				29.82	2.48		
		3.1^d^	36.82(6)^d^							
[FeL]	28.59(2)^e^				32.43	32.43	27.34		32.43	
	28.7(1)^d^									
										
[GaH_2_L]			38.11(3)^f^	2.20^d^						
				2.15(4)^e^						
[GaHL]	29.44(7)^f^	2.65^d^	35.91(7)^d^	8.13^d^			27.92(3)^f^	2.36^d^		
		2.53(2)^e^						2.24(1)^e^		
[GaL]	26.79(2)^d^	9.79^d^	27.60(6)^d^		29.79	29.79	25.56(1)^d^		29.79	
										
[ZrHL]			45.9(3)^f^	2.6^d^			36.4(5)^f^	2.15^d^		
				2.2(1)^e^				2.2(2)^e^		
[ZrL]	34.8(2)^f^	5.48^d^	43.3(1)^d^		35.46(5)^f^	7.03^d^	34.25(5)^d^	5.43^d^	35.54(9)^f^	
		5.34(5)^e^				7.2(1)^e^		5.5(2)^e^		
[ZrLH_–1_]	29.32(8)^d^				28.31(7)^d^		28.8(1)^d^			

a Charges omitted for clarity.

b The protonation constants of the ligands together and the stability constants of Fe(III) and Ga(III) complexes were taken from the literature.

c The protonation constants of the ligands and the stability constants of Fe(III) complexes were taken from the literature.

d Determined by potentiometric titrations.

e Determined by pH-dependent UV–vis titrations.

f Determined by metal–metal competition titrations; all measurements performed at 25 °C and *I* = 0.1 M NaClO_4_.

#### ESI-MS: Stoichiometry Evaluation

The stoichiometry of the complexes was evaluated by ESI-MS, frequently used as the first step in the determination of metal complex stoichiometry and already previously employed.^35,46,47^ When the fact that ESI-MS is not able to distinguish the ionizable protons in the species is taken into account, this method can be successfully applied to evaluate the metal to ligand stoichiometry directly from the *m*/*z* values. For all of the investigated systems, an analysis of the ESI-MS data (collected for various metal to ligand molar ratios) revealed only mononuclear complexes (for details see Figure S3 and Table S1 in the Supporting Information).

#### Determination of Complex Stability

To evaluate the thermodynamic stability of Ga(III) and Zr(IV) complexes of the investigated ligands, the binding properties and speciation of Fe(III) complexes have first been determined (all the details are given in the Supporting Information). There are several reasons for this protocol. First, (i) the electron configuration of Ga(III) (d^10^) and Zr(IV) (d^0^) hinders the attainment of spectral information for most of the complexes. Furthermore, (ii) both metal ions are highly acidic and they are readily hydrolyzed over almost the entire pH range. In addition, (iii) the high charge to size ratio of the Zr(IV) ion implies the formation of complexes with an exceptional thermodynamic stability (already at very low pH); as a consequence, the stability constants cannot be directly determined using standard potentiometric titrations. Thus, the thermodynamic stability constants of the Fe(III)-**H**_**3**_**L1** and Fe(III)-**H**_**4**_**L2** systems were first determined (using a combination of potentiometric and pH-dependent UV–vis titrations), followed by Fe(III)–Ga(III) and Fe(III)–Zr(IV) metal–metal competition experiments. Of importance, in order to get accurate results, an experiment where two metal ions compete for a ligand must fulfill two basic requirements: (i) one of the metal chelates should have a strong absorption band in teither he visible or ultraviolet region of the spectrum, with an extinction coefficient much different from that of the free metal ion, while the second metal complex should not absorb in the same region of the spectrum; (ii) the equilibrium constant for the competition reaction must not be too small or too large. These requirements are fulfilled in the competition experiments between Fe(III) and Ga(III) for both ligands. In the case of Fe(III)–Zr(IV) competition, the difference between the stability constants of Fe(III) and Zr(IV) complexes was too high; thus, an additional competitor ligand—nitrilotriacetic acid (NTA)—was used in the titrations.

NTA is one of the most widely investigated and often used chelating agents.^48,49^ It was selected for the current studies, as both Zr(IV)-NTA and Fe(III)-NTA complexes remain stable until pH 4, even at a metal to ligand molar ratio of 1:1.^27,48^ Moreover, as an additional competing agent, NTA prevents the hydrolysis of the metal ions present in solution and weakens the transchelation observed in the case of **H**_**3**_**L1** and **H**_**4**_**L2** Fe(III) complexes titrated directly by Zr(IV) ions. The accuracy of the metal–metal competition titration with NTA was checked on the Zr(IV)-DFOB system, for which experimental data gave log β_[ZrHDFOB]_ = 46.1(2) (see the Supporting Information), in very good agreement with our recently reported data (log β_[ZrHDFOB]_ = 47.7, allowing for changes in the ionic strength).^24^ Similar competition procedures are widely used for the evaluation of the stability constants of spectroscopically blind metal complexes.^25,50^

#### Ga(III) Complex Formation Equilibria

**Figure 1:**
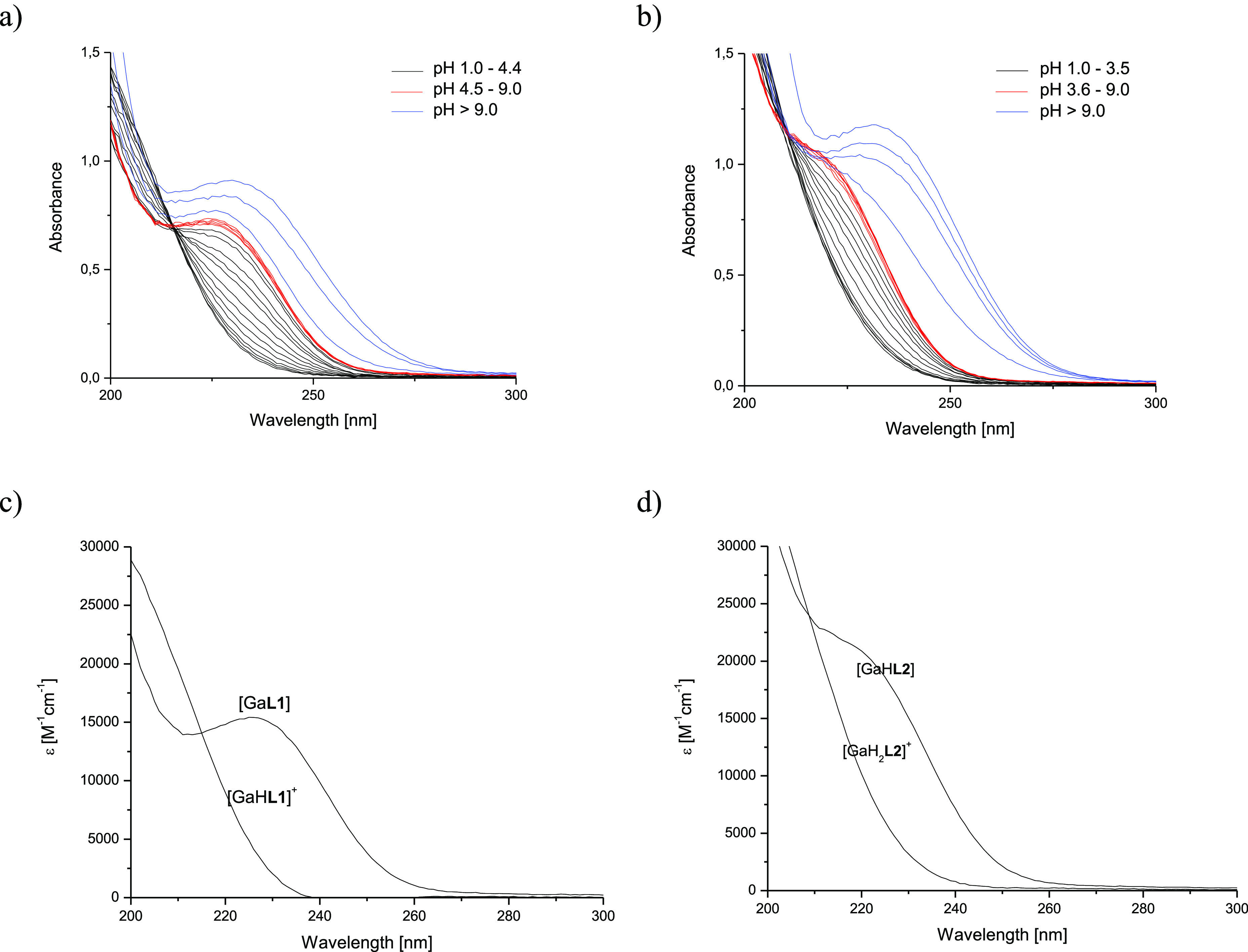
UV spectra of Ga(III)-**H**_**3**_**L1** (a, c) and Ga(III)-**H**_**4**_**L2** (b, d) systems at a metal to ligand molar ratio of 1:1 in the pH range 1.0–11.0. Conditions: *c*_**L1**_ = 0.05 mM, *c*_**L2**_ = 0.05 mM, 0.1 M NaClO_4_, *T* = 25 °C.

The evaluation of thermodynamic stability constants of Ga(III) and Zr(IV) complexes with **H**_**3**_**L1**, **H**_**4**_**L2**, and **H**_**3**_**L4** started from pH-dependent UV–vis spectrophotometric titrations in the pH range 1–11 (Figure 1 and Figure S7, respectively). The spectral changes in the 200–300 nm range, corresponding to the hydroxamate group protonation state, were monitored.^51^ The appearance of a band with a maximum at 225–230 nm with an increase in pH was observed and associated with complex formation.

The pH-dependent UV–vis titration experiments for the Ga(III)-**H**_**3**_**L1** system revealed an increase in a 230 nm transition band starting from pH 1 up to pH 4.4, with p*K*_a_ = 2.53(2) (Figure 1 and Table 1). For the Ga(III)-**H**_**4**_**L2** system, the 225 nm band development was observed starting from pH 1 up to pH 3.5, with p*K*_a_ = 2.15(4) (Figure 1 and Table 1), while for Ga(III)-**H**_**3**_**L4**, it continued to increase up to pH 4.6 with p*K*_a_ = 2.36(1) (Figure S7 and Table 1). Similar behavior was observed in the acidic range for Ga(III)-DFOB (and was assigned to two protonation constants of the complex, p*K*_a1_ = 0.78 and p*K*_a2_ = 1.10^43^) and Th(IV)-DFOB (with p*K*_a_ = 1.9^42^) complexes. For the three investigated systems, UV spectra did not reveal any additional changes up to pH 9, indicating that the fully coordinated complex [Ga**L**], in the case of the Ga(III)-**H**_**3**_**L1** and Ga(III)-**H**_**3**_**L4** systems, and the monoprotonated [GaH**L2**]^+^ complex, in the Ga(III)-**H**_**4**_**L2** system, are the dominant species in solution. When the pH was increased to above 9, an increase in absorbance below 240 nm was observed (Figure 1 and Figure S7). Considering that the spectrophotometric titrations of the free ligands showed the same absorption curves at higher pH, we can suppose that the sharp band at 230–240 nm arises from unbound deprotonated hydroxamate chromophores. Most probably, at higher pH the complex is dissociated, yielding the hydrolyzed gallium species [Ga(OH)_4_]^−^. A similar observation has already been noted for hydroxamate ligands.^43,52^

**Figure 2:**
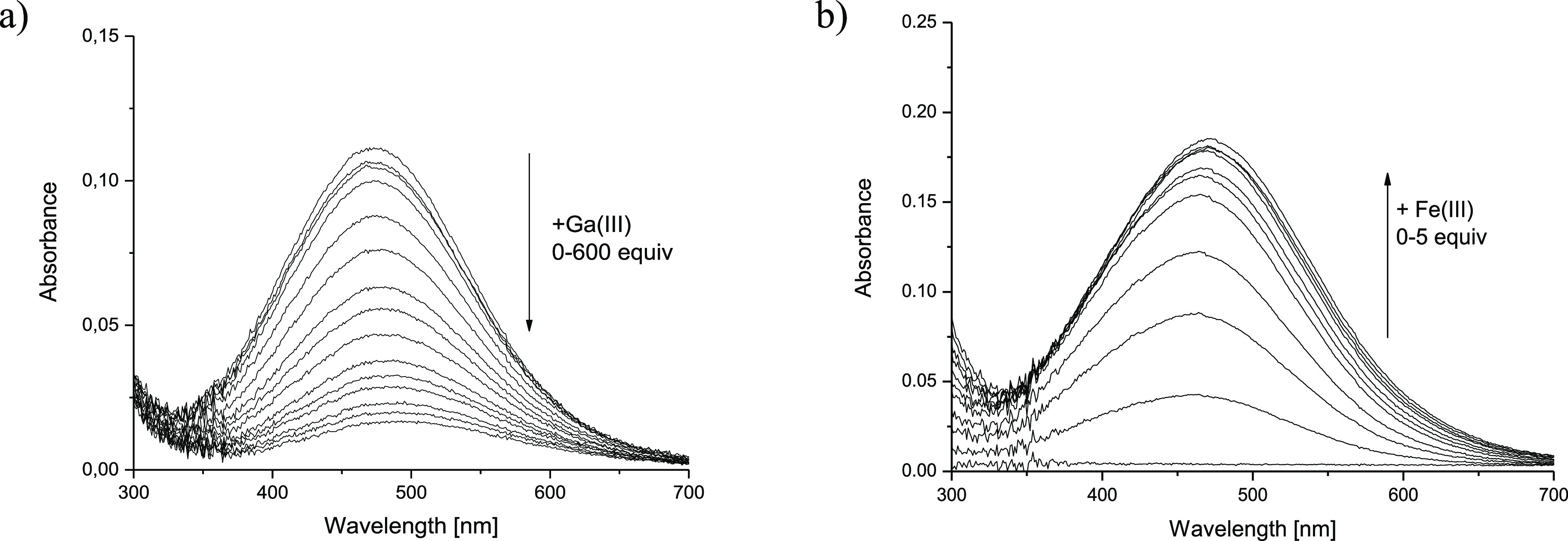
UV–vis metal–metal competition experiment for (a) Fe(III)-**H**_**3**_**L1**+Ga(III) (*c*_Fe(III)_ = 0.075 mM, *c*_L_ = 0.075 mM) and (b) Ga(III)-**H**_**3**_**L1**+Fe(III) (*c*_Fe(III)_ = 0.08 mM, *c*_**L1**_ = 0.08 mM) systems at pH 1.5, *I* = 0.1 M (NaClO_4_), and *T* = 25 °C.

Assuming the domination of the [GaH**L1**]^+^ complex below pH 2, its stability was determined by metal–metal competition titrations, (i) Fe(III)-**H**_**3**_**L1** + Ga(III) and (ii) Ga(III)-**H**_**3**_**L1** + Fe(III), both performed at pH 1.5. This pH was chosen in order to prevent the hydrolysis of the free metal ions and decomposition of the ligand, which is common for hydroxamic acids at a very acidic pH.^37,51^ Upon addition of Ga(III) (up to 600 equiv) to the Fe(III)-**H**_**3**_**L1** solution, the UV–vis band of [FeH**L1**]^+^ (λ_max_ = 470 nm, Figure 2a) slowly disappeared as a result of the [GaH**L1**]^+^ complex formation. In the next competition experiment, the appearance of an LMCT transition band (λ_max_ = 470 nm) upon addition of Fe(III) to the Ga(III)-**H**_**3**_**L1** solution was observed (Figure 2b). The data refinement using **H**_**3**_**L1** protonation constants (Table 1), Fe(III)-**H**_**3**_**L1** stability constants (Table 1), and stability constants of hydroxocomplexes of both metals (see the Experimental Section) yielded a log β_[GaH**L1**]^+^_ value of 29.44(7) for Fe(III)-**H**_**3**_**L1** + Ga(III) (Table 1) and of 28.3(6) in the case of Ga(III)-**H**_**3**_**L1** + Fe(III) competition titrations.

For the Ga(III)-**H**_**4**_**L2** system, [GaH_2_**L2**]^+^ was assumed to be the most abundant species at pH 1.5, and its stability was again determined via (i) Fe(III)-**H**_**4**_**L2** + Ga(III) and (ii) Ga(III)-**H**_**4**_**L2** + Fe(III) titrations (Figure S8). The refinement of the titration data yielded log β_[GaH_2_**L2**]^+^_ = 38.11(3) and 39.06(4) for Fe(III)-**H**_**4**_**L2** + Ga(III) and Ga(III)-**H**_**4**_**L2** + Fe(III) competition titrations, respectively.

For the Ga(III)-**H**_**3**_**L4** system, [GaH**L4**]^+^ was assumed to be the major complex at pH 1.5, and its stability was determined via an Fe(III)-**H**_**3**_**L4** + Ga(III) competition titration (Figure S9). The refinement of the titration data yielded log β_[GaH**L4**]^+^_ = 27.92(3).

For the Ga(III)-**H**_**3**_**L1** and Ga(III)-**H**_**4**_**L2** systems, the constants obtained with the two types of titrations are not far from each other but are significantly different. Most likely, the constants measured by means of Ga(III)-**H**_**3**_**L1**/**H**_**4**_**L2** + Fe(III) competition experiments are endowed with a greater error, attributable to the overlapping absorption of free iron, present in excess. Therefore, we retained the constants obtained from the Fe(III)-**H**_**3**_**L1**/**H**_**4**_**L2** + Ga(III) competition experiments (log β_[GaH**L1**]^+^_ = 29.44(7) and log β_[GaH_2_**L2**]^+^_ = 38.11(3)) as fixed values in the subsequent potentiometric data calculations. The best-fitted speciation model for Ga(III)-**H**_**3**_**L1** and Ga(III)-**H**_**3**_**L4** systems revealed the presence of one additional complex, [Ga**L**], with log β values of 26.79(2) and 25.56(1), respectively (p*K*_a1_ = 2.65 for Ga(III)-**H**_**3**_**L1** and 2.36 for Ga(III)-**H**_**3**_**L4**; Table 1). For the Ga(III)-**H**_**4**_**L2** system, in addition to [GaH_2_**L2**]^+^, [GaH**L2**] and [Ga**L2**]^−^ complexes were found with log β_[GaH**L2**]_ = 35.91(7) and log β_[Ga**L2**]^−^_ = 27.60(6) (p*K*_a1_ = 2.20 and p*K*_a2_ = 8.13; Table 1). The p*K*_a2_ value of 8.13 is in good agreement with the p*K*_a_ values of the free ligand (Table 1) and could be assigned to the deprotonation of an unbound hydroxamate group.

#### Zr(IV) Complex Formation Equilibria

**Figure 3:**
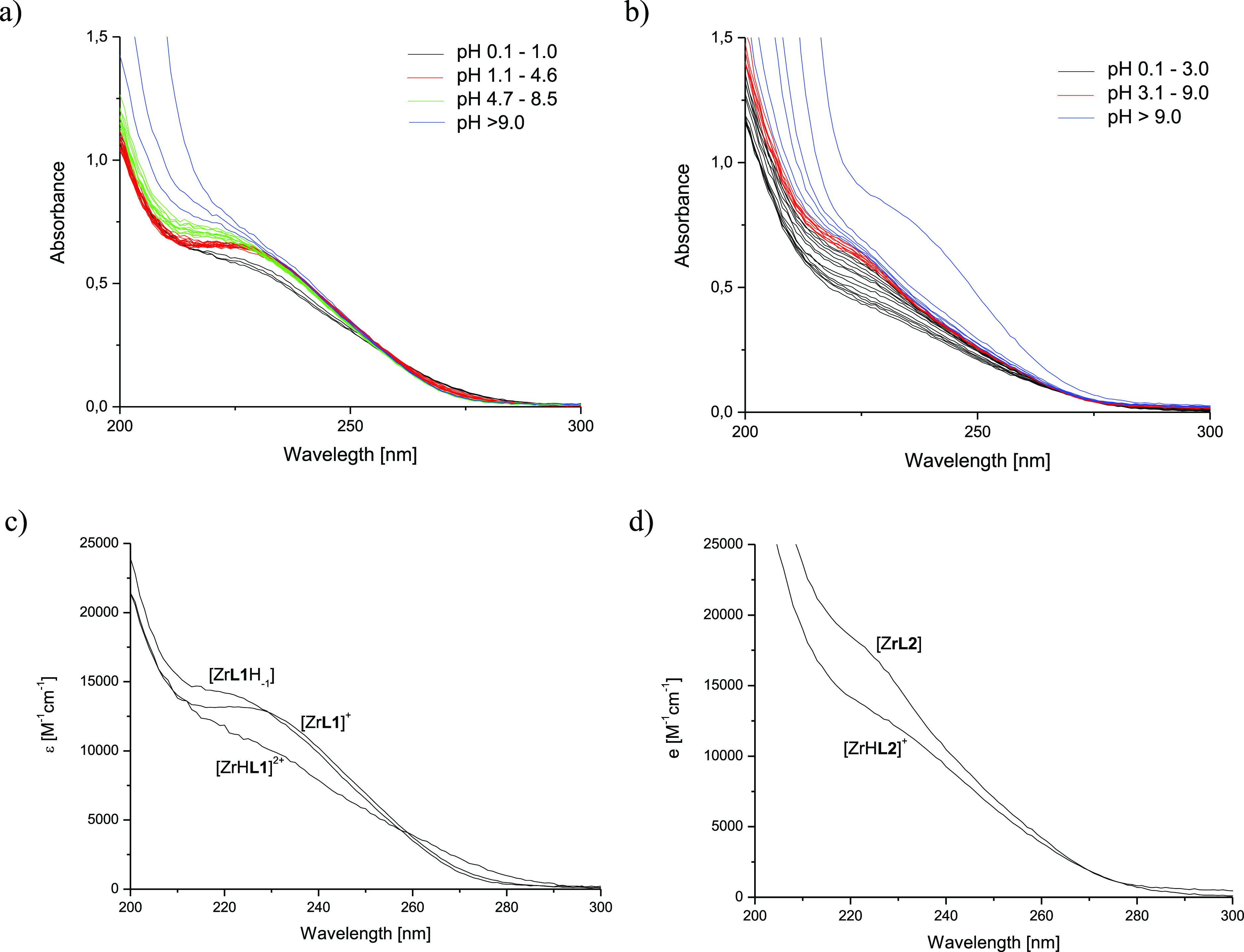
UV spectra of the Zr(IV)-**H**_**3**_**L1** (a, c) and Zr(IV)-**H**_**4**_**L2** (b, d) systems at a metal to ligand molar ratio of 1:1 in the pH range 0.1–11 Conditions: *c*_**L1**_ = 0.05 mM, *c*_**L2**_ = 0.037 mM, 0.1 M NaClO_4_.

**Figure 4:**
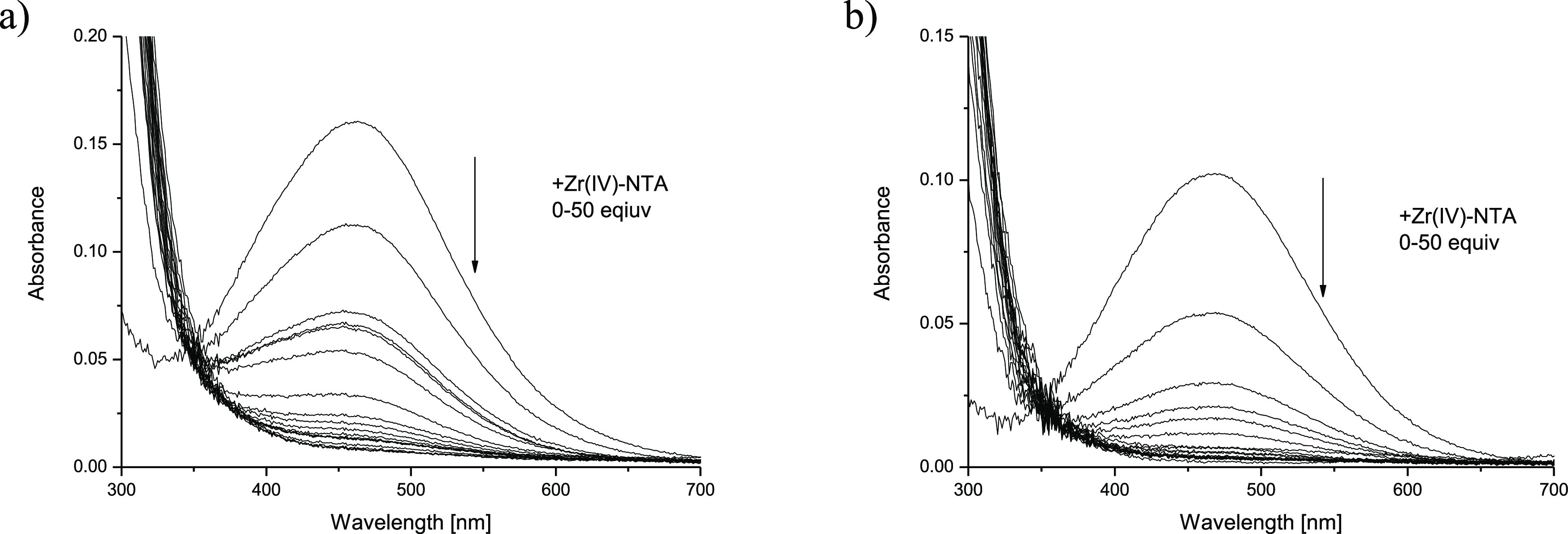
UV–vis spectra of competition titrations of the Fe(III)-**H**_**3**_**L1** + Zr(IV)-NTA (*c*_Fe(III)_ = 0.098 mM, *c*_**L1**_ = 0.098 mM) (a) and Fe(III)-**H**_**4**_**L2** + Zr(IV)-NTA (*c*_Fe(III)_ = 0.055 mM, *c*_**L2**_ = 0.055 mM) (b) systems at pH 1.5 with 0.1 M NaClO_4_.

The UV–vis titrations of Zr(IV)-**H**_**3**_**L1** equimolar solution over the pH range 0.1–11 (Figure 3a,c) showed a well-defined absorbance band in the 200–300 nm range. The significant changes and the presence of an isosbestic point observed when the pH was increased from 0.1 to 0.9, allowed us to calculate a p*K*_a1_ value of 0.4(2); afterward, the observed 230 nm shoulder remained stable up to pH 4.6. When the pH was increased to 7.0, the development of a 220 nm shoulder with an isosbestic point at 230 nm was observed, characterized by p*K*_a2_ = 5.34(5). From pH 7.0, the spectra do not reveal any significant changes until pH 9.0, where the hydrolysis probably started. Since information about hydrolysis of the hydroxamate ligands at acidic pH has beenwidely described in the literature,^51,52^ p*K*_a1_ = 0.4 indicates that the three hydroxamate groups are dissociated and therefore most probably bound to the Zr(IV) ion, already at pH <2.0. Assuming the formation of only monomeric complexes, the stability of [Zr**L1**]^+^ was determined via UV–vis competition batch experiments, using a Zr(IV)-NTA solution as a competing system for the Fe(III)-**H**_**3**_**L1** complex (Figure 4a). The large LMCT band centered at 470 nm characteristic of dihydroxamate [FeH**L1**]^+^ species decreased gradually, and the refinement of the titration data, using the Fe(III)-**H**_**3**_**L1** stability constants (Table 1) together with the Fe(III) and Zr(IV) hydrolysis constants, yielded a log β_[Zr**L1**]^+^_ value of 34.8(2).

The UV–vis titration data of the Zr(IV)-**H**_**4**_**L2** system (Figure 3b,d) showed the development of a 230 nm shoulder upon an increse in pH from 0.1 to 3.0 and allowed us to calculate a p*K*_a_ value of 2.2(1). Further, the spectra did not reveal any significant changes over the pH range 3.1–9.0, wherethe hydrolysis probably started. [ZrH**L2**]^+^ was assumed to be the major complex at pH 1.5, and its stability was determined via Fe(III)-**H**_**4**_**L2** + Zr(IV)-NTA (Figure 4b) competition titrations. The refinement of the titration data yielded log β_[ZrH**L2**]^+^_ = 45.9(3).

The UV–vis spectra of the Zr(IV)-**H**_**3**_**L3** and Zr(IV)-**H**_**3**_**L4** systems were very similar to the spectra described above (Figure S11); at a pH of around 7 a slight blue shift of the 230 nm band appeared together with the isosbestic point, allowing us to calculate p*K*_a_ = 7.2(1) for Zr(IV)-**H**_**3**_**L3** and 5.5(2) for Zr(IV)-**H**_**3**_**L4** (Table 1). For Zr(IV)-**H**_**3**_**L4**, an additional p*K*_a_ = 2.2(2) was calculated, probably corresponding to the formation of the three-hydroxamate complex (Table 1). An evaluation of the competition titration data for Fe(III)-**H**_**3**_**L3** + Zr(IV)-NTA revealed log β_[Zr**L3**]^+^_ = 35.46(5), while for Fe(III)-**H**_**3**_**L4** + Zr(IV)-NTA log β_[ZrH**L4**]^2+^_ = 36.4(5) (Figure S12 and Table 1). Additionally, we have performed the same kind of titration for Fe(III)-**DFOE** + Zr(IV)-NTA, which gave log β_[Zr**DFOE**]^+^_ = 35.54(9) (Figure S12 and Table 1). It is worth noting that, during the competition titrations for the **H**_**3**_**L3** ligand and **DFOE**, a decrease in the 430 nm (typical for three-hydroxamate iron complexes) band was observed, confirming that all three hydroxamate groups are bound already at pH <2.^38^

Using the constants obtained from competition titrations (log β_[Zr**L1**]^+^_ = 34.8(2) and log β_[ZrH**L2**]^+^_ = 45.9(3)) as fixed values, the potentiometric data were processed. The best-fitted speciation models revealed the presence of one additional complex for the Zr(IV)-**H**_**3**_**L1** system ([Zr**L1**H_–1_], log β_[Zr**L1**H_–1_]_ = 29.32(8)) and one additional complex for the Zr(IV)-**H**_**4**_**L2** system ([Zr**L2**], log β_[Zr**L2**]_ = 43.3(1)) (Table 1).

The [Zr**L1**]^+^ complex dominates the solution from pH 3 up to pH ∼5, where its deprotonation to [Zr**L1**H_–1_] occurs, with p*K*_a_ = 5.48 (Figure S13a). These results are in line with the spectroscopic data (p*K*_a_ = 5.34(5)) and could be ascribed to the dissociation of a water molecule from the coordination sphere of the Zr(IV) ion. For the Zr(IV)-EDTA system, the p*K*_a_ attributed to the deprotonation of a water molecule is 6.2,^27^ while for Zr(VI)-DFOB it is 6.36.^24^

The stability constants calculated for the Zr(IV)-**H**_**4**_**L2** system reveal p*K*_a_ = 2.6, which is in good agreement with the spectroscopic data (p*K*_a_ = 2.2(1)) and could be assigned to the deprotonation of the hydroxamic group and the formation of a fully coordinated tetrahydroxamate complex. The [Zr**L2**] complex dominates the solution from pH 3 up to pH ∼9, when the dissociation of the complex probably occurs (Figure S13b).

An evaluation of the potentiometric data of Zr(IV)-**H**_**3**_**L3** (with log β_[*Zr***L3**]_ = 35.46 fixed) revealed the presence of an additional complex, [Zr**L3**H_–1_] (log β_[Zr**L3**H_–1_]_ = 28.35(5)) (Figure S13c), while for Zr(IV)-**H**_**3**_**L4** (with log β_[ZrH**L4**]_ = 36.4 fixed), there are two additional complexes, [Zr**L4**] (log β_[Zr**L4**]_ = 34.25(5)) and [Zr**L4**H_–1_] (log β_[Zr**L4**H_–1_]_ = 28.8(1)) (Figure S13d). For both systems, the p*K*_a_ values calculated from potentiometric experiments are in excellent agreement with those from the pH-dependent UV–vis titrations (Table 1) and match the data obtained for the whole series of hydroxamate-based ligands. Of importance, the p*K*_a_ value attributed to the deprotonation of a water molecule in the Zr(IV)-**H**_**3**_**L3** system (p*K*_a_ = 7.2) is higher than those for Zr(VI)-DFOB (p*K*_a_ = 6.36)^24^ and Zr(IV)-**H**_**3**_**L1** (p*K*_a_ = 5.48), suggesting that a longer linker between the binding groups is advantageous. In Zr(IV)-**H**_**3**_**L4**, this process is not observed, as all the coordinating positions of the Zr(IV) ion are occupied by four hydroxamate ligands.

#### Ligand Sequestering Ability

Despite a large variety of PET chelators synthesized and tested in order to provide strong coordination of Ga(III) and Zr(IV) *in vivo*, until now it is has been hard to avoid the release of these metal ions in the body. Here we report two new Ga(III) and Zr(IV) hydroxamate chelators, designed to achieve an efficient sequestering of these metal ions but also to understand how the cyclization and the introduction of an additional hydroxamate group influences the stability of Zr(IV) complexes. Therefore, it is important to evaluate and compare the Ga(III)- and Zr(IV)-sequestering abilities of **H**_**3**_**L1** and **H**_**4**_**L2** with those of other chelators. However, the direct comparison of the stability constants of metal complexes is not straightforward, and other tools taking into account all the physicochemical properties of the ligands, i.e. their denticity, coordination modes, and acid–base properties, should be used.^52^ In order to reliably compare the chelating abilities of **H**_**3**_**L1** and **H**_**4**_**L2** toward Ga(III) and Zr(IV) ions, the pM values were calculated. pFe was originally introduced by Raymond for the comparison of iron-siderophore systems;^53^ pGa = −log[Ga(III)_free_] and pZr = −log[Zr(IV)_free_] were calculated at pH 7.4 with *c*_L_ = 10 μM and *c*_Ga(III)/Zr(IV)_ = 1 μM (Table 2).

**Table 2:** pGa and pZr Values for Various Synthetic and Natural Chelators^a^

ligand	pGa	pZr	chelating groups and ligand geometry
**H**_**3**_**L1**	22.5	32.4	3 hydroxamate groups in a cyclic arrangement
**H**_**4**_**L2**	22.3	37.0	4 hydroxamate groups in a linear arrangement
**H**_**3**_**L3**	25.4	31.5	3 hydroxamate groups in a cyclic arrangement
**H**_**3**_**L4**	21.9	32.6	3 hydroxamate groups in a linear arrangement
DFOB	21.6^43^	32.2^24^	3 hydroxamate groups in a linear arrangement
**DFOE**	25.2^38^	31.0	3 hydroxamate groups in a cyclic arrangement
DOTA	20.5^11^		4 macrocyclic amine groups and 4 carboxylate pendant arms
NOTA	27.4^54^		3 macrocyclic amine groups and 3 carboxylate pendant arms
H_2_hox	28.4^55^		2 8-hydroxyquinoline groups and 2 amino groups in a linear arrangement
PrP9	23.1^56^		3 macrocyclic amine groups and 3 carboxylate pendant arms
HBED	28.0^57^		2 hydroxyaromatic donor groups and 2 carboxylate pendant arms
THPN		42.7^2^	4 3-hydroxy-4-pyridinone pendant arms
3,4,3-LI-HOPO		44.0^25,58^	4 1-hydroxy-2-pyridinonates in a linear arrangement
DTPA		32.3^25,59^	3 amino groups in linear arrangement and 4 carboxylate pendant arms

a Values (re)calculated at pH 7.4 and *c*_L_ = 10 μM and *c*_Ga(III)/Zr(IV)_ = 1 μM, on the basis of the protonation and stability constants given in original publications. The hydrolysis constants of Ga(III) and Zr(IV) ions were taken from the literature^60^ and are given in the Experimental Section.

The pGa values for Ga(III)-**H**_**3**_**L1** and Ga(III)-**H**_**4**_**L2** systems are on the same order of magnitude as those of the well-known gallium chelators DFOB and PRP9 but are higher than that of the clinically used DOTA (Table 2). On the other hand, H_2_hox,^55^ NOTA,^54^ and HBED^57^ present much higher Ga(III) chelating efficacy. The observed effect reflects the differences in the number and type of chelating groups present in the ligands (and therefore the number and type of donor atoms), as well as the ligand dimensions. Of importance, there is only about a 1 order of magnitude increase between pGa values for the linear trihydroxamate ligand DFOB and the cyclic tri- and tetrahydroxamates **H**_**3**_**L1** and **H**_**4**_**L2**, respectively. This indicates that the cyclization of the structure only slightly influences the complex stability. It is worth underlining that **H**_**3**_**L1** has shorter spacers between the hydroxamate groups (9 bonds) in comparison to those in DFOB and **DFOE** (10 bonds). In **H**_**3**_**L3**, the spacers have the same length of 10 bonds and the Ga(III) complexes reach the stability of **DFOE**.^38^ Additionally, there is almost no difference in complex stability between Ga(III)-**H**_**3**_**L1** and Ga(III)-**H**_**4**_**L2**, even though the cavity of **H**_**4**_**L2** is much larger than that of **H**_**3**_**L1**, allowing higher flexibility and entropy of the complex structure.

For the Zr(IV) complexes of trihydroxamate **H**_**3**_**L1**, **H**_**3**_**L3**, and **H**_**3**_**L4** systems, the pZr value is on the same order as those for DFOB^24^ and DTPA^25^ chelators. This suggests that ligand cyclization does not provide any increase in complex stability with respect to its linear analogue; for **H**_**3**_**L3** and **DFOE** one may even claim a slight decrease in relation to DFOB. A similar conclusion was drawn from the comparison of Zr(IV) complexes of DFOB with fusarinine C (FCS, Scheme 1), where only minor differences in complex stability were observed in *in vivo* studies.^61^ An elongation of the chain between hydroxamate binding units from 9 bonds in **H**_**3**_**L1** to 10 in **H**_**3**_**L3** is not reflected in the corresponding pZr values. The flexibility of the tripodal **H**_**3**_**L4** ligand does not produce a higher stability of Zr(IV) complexes. Of importance, the pZr value for the tetrahydroxamate analogue **H**_**4**_**L2** is >4 units higher than the values calculated for Zr(IV)-DFOB and Zr(IV)-**H**_**3**_**L1** systems, reflecting the higher affinity of tetrahydroxamate **H**_**4**_**L2** for Zr(IV), as expected. This feature was already observed in biological studies of other tetrahydroxamate chelators.^4,39,41^ The high thermodynamic stability is certainly the result of the involvement of the fourth hydroxamate coordinating group of the ligand moiety.

The **H**_**3**_**L1** and **H**_**4**_**L2** ligands are very good examples to directly compare the stability of Zr(IV) complexes formed with tri- and tertrahydroxamate compounds. For these two ligands, we observe an increase in log β of 8.5 orders of magnitude for the Zr(IV)-**H**_**4**_**L2** complex with respect to Zr(IV)-**H**_**3**_**L1** (Table 1). However, this increase is lower than that predicted from the computational calculations performed by Holland^32^ for tri- and tetrahydroxamate chelators: i.e., DFOB (log β_[Zr(DFOB)]_ = 41.20) versus linear DFO*^41^ (log β _[Zr(DFO*)]_ = 51.56) and cyclic CTH36^39^ (log β_[Zr(CTH36)]_ = 52.84). Also, log β_[Zr**L2]**_ = 43.3(1) does not reach the values predicted from the above calculations for eight-coordinate Zr(IV)-DFO* and Zr(IV)-CTH36. Of note, the log β_[ZrH(DFOB)OH]_ value previously determined by us for DFOB (40.04)^24^ matches very well the computationally predicted log β_[Zr(DFOB)]_ value (41.20).^32^ Still, in the [ZrH(DFOB)OH]^+^ complex, dominating at pH 6.5–10.5, we have suggested the presence of an unbound protonated amino group and a hydrolyzed water molecule bound to Zr(IV). For the cyclic trihydroxamate ligand **H**_**3**_**L1**, characterized by 9-bond linkers between its hydroxamate units (Scheme 1), log β_[Zr**L1]**_ = 34.8(2) is not far from the value estimated for the trihydroxamate cyclic siderophore FSC,^7,62^ log β_[Zr(FSC)]_ = 38.92;^32^ this difference can be ascribed to the alterations in geometry and dimensions of the ligands. Zirconium is known to form complexes with a complicated geometry of a dodecahedron or square antiprism,^63,64^ and numerous DFT studies have revealed that minor variations in ligand geometry (such as a pendant arm elongation or a modification of the ligand cavity size) could result in significant changes in the stability of Zr(IV) complexes.^39,65,66^**H**_**4**_**L2** presented in this work possesses four hydroxamate units and amide units in the linker, but a larger cavity size and a significant asymmetry (coming from one much longer linker and with two amides and amino group) with respect to CTH36 (Scheme 1).^32,39^ These structural alterations are most probably the reason for the lower thermodynamic stability of **H**_**4**_**L2** complexes, as the coordination sphere might not be uniformly closed around the central ion. Unfortunately, the thermodynamic characterization of Zr(IV)-CTH36 complexes has not yey been reported; thus, the pZr value cannot be quantified. Another cyclic hydroxamic ligand, PPDDFOT_1_, that possesses four hydroxamic groups in a symmetrical arrangement and a cavity size even larger than that of **H**_**4**_**L2** (11 bonds between hydroxamic groups) showed superior stability vs DFOB in EDTA challenging assays.^67^ These results confirm that the Zr(IV) complex stability is strongly dependent on the ligand geometry and emphasize the demand for a thermodynamic solution study in order to understand this dependence. Other octadentate hydroxy-pyridinone chelators, such as THPN^2^ and 3,4,3-LI-HOPO,^25^ form the strongest complexes (Table 2). The reason could be not only the type of chelating groups present in the ligands but also the ligand architecture and dimensions.

## Conclusions

In the present work we have developed new chelating agents for the complete saturation of the coordination spheres of Ga(III) and Zr(IV) metals. The trihydroxamic (**H**_**3**_**L1**) and tetrahydroxamic (**H**_**4**_**L2**) ligands were successfully synthesized, and the thermodynamic properties of their Ga(III) and Zr(IV) complexes were evaluated. In addition, a series of other synthetic (**H**_**3**_**L3**, **H**_**3**_**L4**) and natural (**DFOE**) compounds was investigated. **H**_**3**_**L1** proved to be an efficient Ga(III) chelator, but the stability of its Zr(IV) complexes is about 1 order of magnitude lower than that reported for the Zr(IV)-DFOB system. **H**_**4**_**L2** is the first tetrahydroxamate ligand for which the formation constants and speciation with Zr(IV) were experimentally determined. Of importance, it revealed an enhanced stability of 8.5 orders of magnitude (log β_[Zr**L2**]_**=** 43.3, pZr = 37.0) with respect to Zr(IV)-**H**_**3**_**L1** (log β_[Zr**L1**]_**=** 34.8, pZr = 32.4), as a consequence of the introduction of a fourth hydroxamate binding unit. However, the stability increase is lower than that predicted by computational calculations for the tetrahydroxamate chelators DFO* (log β_[Zr(DFO*)]_ = 51.56) and CTH36 (log β_[Zr(CTH36)]_ = 52.84), and this effect can be ascribed to the structural alterations of the **H**_**4**_**L2** ligand.

Overall, the determination of the thermodynamic stability of metal complexes coupled with a suitable chelator design will help further developments of optimal chelators for PET imaging applications. However we are aware that there are still a great number of tests to do in order for these ligands to be used as a PET chelators, such as radiolabeling and kinetics studies, biodistribution assays, etc. Current efforts are focused on the design and studies of tetrapodal hydroxamate ligands, to interrogate how their shape and size tune the thermodynamic stability of Zr(IV) complexes.

According to the literature, the ^89^Zr radiolabeling strategies for hydroxamate ligands is usually simple, robust, and relatively rapid. They are performed under mild pH conditions, at room temperature, and take around 1 h for DFOB derivatives on their own without being attached to any targeting vectors and 1–3 h for DFO derivatives attached to targeting vectors, such as trastuzumab.^14,68,69^ Cyclic hydroxamate ligands such as C7^40^ and CTH36^39^ (both with 8 bonds between hydroxamate groups) have demonstrated excellent complexation abilities at ambient temperature (>99% complexation after 120 min for C7 and >90% of the activity within 5 min reaction time for CTH36, respectively). The slightly smaller ligands C6 and C5, reported by Guerard et al.,^40^ appear to be less suitable for radiolabeling, with higher temperatures being required to obtain high complexation yields. The cyclic ligands reported in this paper possess a larger cavity size, with at least 9 bonds between hydroxamate groups, which allows us to assume that the radiolabeling process will be highly efficient and performed under mild conditions. Preliminary radiolabeling of artificial FOXE siderophores with ^68^Ga, represented here by FOXE 2–5, was achieved after 10 min at room temperature with moderate yields and high specific activities of ^68^Ga.^38^ Further characterization is ongoing.

## Experimental Section

### Synthesis

#### General Considerations

Unless stated otherwise, all commercially available reagents and solvents were of analytical grade. For the synthesis of **H**_**3**_**L1** and **H**_**4**_**L2**, solvents and reagents were purchased from Bachem, BLDpharm, and Fluka. Crude products were purified via flash column chromatography on silica gel (Merck, 230–400 Mesh) or, for compounds **10**, **11**, **16**, and **17**, by semipreparative RP-HPLC using a Waters Prep 600 system equipped with a C18 Jupiter column (250 × 30 mm, 300 Å, 15 μm spherical particle size). Gradients were established each time by considering the analytical HPLC profile of the crude product. The column was perfused at a flow rate of 20 mL/min over 30 min with a binary system of solvent A (H_2_O + 0.1% v/v TFA) and solvent B (60% CH_3_CN in water +0.1% v/v TFA). Analytical RP-HPLC analyses were performed on a XBridge C18 column (4.6 × 150 mm, 5 μm particle size) using a flow rate of 0.7 mL/min and a linear gradient of acetonitrile (and 0.1% TFA) in water (and 0.1% TFA) from 0% to 100% over 25 min. The mass spectra were recorded on an ESI-Micromass ZMD 2000 instrument. TLC was performed on precoated plates of silica gel F254 (Merck, Darmstadt, Germany). ^1^H NMR analyses were obtained using a Varian spectrometer (400 MHz) and were referenced to residual ^1^H signals of the deuterated solvents (δ(^1^H) 7.26 for CDCl_3_; δ(^1^H) 2.50 for DMSO). The following abbreviations are used to describe the shape of the peaks: s, singlet; d, doublet; dd, doublet of doublets; t, triplet; m, multiplet.

#### Synthesis of *tert*-Butyl(benzyloxy)carbamate (**1**)

To an ice-cooled solution of *O*-benzylhydroxylamine·HCl (4.00 g, 25 mmol) in a 1,4-dioxane/H_2_O mixture (60 mL, 1/1 v/v) was added K_2_CO_3_ (10.37 g, 75 mmol). Boc_2_O (8.18 g, 37.5 mmol), previously dissolved in dioxane, was then added dropwise, and the reaction mixture was stirred at room temperature overnight. The solvent was removed under vacuum, and the crude product extracted using ethyl acetate (30 mL) and water (3 × 15 mL). The organic phase was dried over Na_2_SO_4_, filtered, and evaporated. Compound **1** (4.86 g, 87% yield) was obtained as a colorless oil, which was used without any further purification. NMR data match those reported in the literature (PMID: 11906271). ESI-MS: calcd for C_12_H_18_NO_3_, 224.28 [M + H]^+^; found, 224.13 [M + H]^+^. *T*_R_ = 19.70 min.

#### Synthesis of Ethyl 4-((Benzyloxy)(*tert*-butoxycarbonyl)amino)butanoate (**2**)

To a solution of **1** (4.86 g, 21.79 mmol) in DMF (15 mL) was added NaH (60% dispersion in mineral oil, 1.20 g, 23.94 mmol). The mixture was initially stirred at rt for 30 min, and then the reaction mixture was warmed to 60 °C and ethyl 4-bromobutyrate was added dropwise. At the completion of the reaction, the solvent was removed, and the residue was extracted with ethyl acetate and water (3 × 30 mL), dried over Na_2_SO_4_, and concentrated under vacuum. The product (**2**) was obtained as a yellowish oil (5.36 g, 73% yield). The NMR data correspond to those in the literature (PMID: 28715615). MS (ESI): calcd for C_18_H_28_NO_5_, 338.20 [M + H]^+^; found, 360.18 [M + Na]^+^, 697.37 [2M + Na]^+^. *T*_R_ = 23.51 min.

#### Synthesis of Ethyl 4-(*N*-(Benzyloxy)-4-((*tert*-butoxycarbonyl)amino)butanamido)butanoate (**4**)

Compound **2** (5.36 g, 15.90 mmol) was dissolved in trifluoroacetic acid (TFA, 6 mL), and the mixture was stirred at room temperature for 2 h. The reaction mixture was monitored by MS (ESI) before being concentrated under vacuum. The deprotected amino ester **3** was used without further purification in the next step. To an ice-cold solution of Boc-γ-aminobutiric acid (2.7 g, 13.45 mmol) in DMF (20 mL) were added 1-[bis(dimethylamino)methylene]-1*H*-1,2,3-triazolo[4,5-*b*]pyridinium 3-oxide hexafluorophosphate (HATU, 5.6 g, 14.75 mmol) and DIPEA (2.6 mL, 14.75 mmol). A portion of **3** (3.5 g, 14.75 mmol) was dissolved in DMF (10 mL), and this solution was added dropwise to the first one. Then the reaction mixture was warmed to room temperature and stirred for 1 h. After removal of the solvent, the residue was dissolved in ethyl acetate and washed with a 5% aqueous solution of citric acid, a 10% aqueous solution of NaHCO_3_, and brine. The crude product was purified by column chromatography using ethyl acetate/petroleum ether (from 1/4 to 1/1 by volume) as an eluent mixture. Compound **4** was obtained as a slightly yellowish oil (4.35 g, 76.6% yield). ESI-MS: calcd for C_22_H_35_N_2_O_6_, 423.53 [M + H]^+^; found, 423.25 [M + H]^+^, 445.23 [M + Na]^+^, 867.47 [2M + Na]^+^. *T*_R_ = 21.11 min. ^1^H NMR (400 MHz, CDCl_3_): δ 7.44–7.32 (m, 5H), 4.80 (s, 2H), 4.11 (qd, *J* = 7.1, 2.9 Hz, 2H), 3.70 (t, *J* = 6.8 Hz, 2H), 3.12 (t, *J* = 6.6 Hz, 2H), 2.42 (t, *J* = 7.2 Hz, 2H), 2.32 (t, *J* = 7.3 Hz, 2H), 1.99–1.91 (m, 2H), 1.80–1.73 (m, 2H), 1.42 (s, 9H), 1.26–1.20 (m, 3H).^13^C NMR (CDCl_3_): δ 172.9, 156.0, 134.3, 129.2, 129.0, 128.7, 79.1, 60.4, 44.6, 40.3, 31.4, 29.6, 28.4, 24.7, 22.3, 14.2.

#### Synthesis of Ethyl 10,20-Bis(benzyloxy)-2,2-dimethyl-4,9,14,19-tetraoxo-3-oxa-5,10,15,20-tetraazatetracosan-24-oate (**7**)

The Boc-deprotected derivative **5** (3.40 g, 7.8 mmol) was obtained as previously described for **3**. Compound **6** was synthesized by dissolving the ethyl ester **4** (3.0 g, 7.1 mmol) in a 1,4-dioxane/H_2_O mixture in the presence of LiOH (1 M aqueous solution, 12.5 mmol). The mixture was stirred at rt for 20–30 min. Once the reaction was complete, dioxane was evaporated and the crude product was acidified using 1 M HCl to reach pH 6. Then, the aqueous phase was extracted using ethyl acetate. Compound **6** (0.91 g, 2.31 mmol) was used in the next step without further purification. The coupling reaction was conducted as previously described for **4**, and derivative **7** was obtained as a yellowish oil (1.24 g, 77% yield) after column chromatography. ESI-MS: calcd for C_37_H_55_N_4_O_9_, 699.87 [M + H]^+^; found, 699.96 [M + H]^+^. *T*_R_ = 21.18 min. ^1^H NMR (400 MHz, CDCl_3_): δ 7.50–7.31 (m, 10H), 7.04 (bs, 1H), 4.81 (d, *J* = 5.3 Hz, 4H), 4.12 (q, *J* = 7.1 Hz, 2H), 3.71–3.69 (m, 4H), 3.26 (dd, *J* = 11.9, 6.2 Hz, 2H), 3.17–3.09 (m, 2H), 2.53–2.39 (m, 4H), 2.33 (t, *J* = 7.3 Hz, 2H), 2.20 (t, *J* = 6.8 Hz, 2H), 1.98–1.92 (m, 4H), 1.84–1.73 (m, 4H), 1.42 (s, 9H), 1.24 (t, *J* = 7.1 Hz, 3H). ^13^C NMR (CDCl_3_): δ 174.7, 173.3, 173.0, 134.2, 129.3, 129.1, 129.1, 128.8, 60.5, 44.7, 44.3, 40.0, 39.6, 33.1, 31.4, 30.0, 29.4, 28.5, 24.8, 23.9, 23.2, 22.3, 14.3.

#### Synthesis of Ethyl 10,20,30-Tris(benzyloxy)-2,2-dimethyl-4,9,14,19,24,29-hexaoxo-3-oxa-5,10,15,20,25,30-hexaazatetratriacontan-34-oate (**9**)

Compound **9** was synthesized under the same coupling conditions used for **4** by starting from the acid derivative **6** (0.91 g, 2.31 mmol) and the amino derivative **8** (1.81 g, 2.54 mmol). The desired product was obtained as a colorless oil (1.89 g, 84% yield) after column chromatography. ESI-MS: calcd for C_52_H_75_N_6_O_12_, 976.20 [M + H]^+^; found, 975.94 [M + H]^+^. *T*_R_ = 17.83.^1^H NMR (400 MHz, CDCl_3_): δ 7.39–7.35 (m, 15H), 5.05 (bs, 3H), 4.83–4.77 (m, 6H), 4.11 (q, *J* = 7.1 Hz, 2H), 3.72–3.67 (m, 6H), 3.34–3.19 (m, 4H), 3.16–3.11 (m, 2H), 2.49–2.45 (m, 6H), 2.32 (t, *J* = 7.3 Hz, 2H), 2.25–2.12 (m, 4H), 2.02–1.89 (m, 6H), 1.87–1.72 (m, 6H), 1.42 (s, 9H), 1.24 (t, *J* = 7.1 Hz, 3H). ^13^C NMR (CDCl_3_): δ 175.3, 174.1, 172.5, 157.3, 135.4, 128.9, 128.3, 128.2, 80.7, 73.8, 61.2, 48.6, 41.2, 40.4, 35.2, 31.8, 31.5, 28.4, 24.1, 21.7, 18.5, 14.7.

#### Synthesis of 1,11,21-Tris(benzyloxy)-1,6,11,16,21,26-hexaazacyclotriacontane-2,7,12,17,22,27-hexaone (**10**)

Compound **9** was Boc-deprotected as described for **3**. Then, the ethyl group was hydrolyzed by LiOH as for **6**. To a dilute solution of the fully deprotected trimer (1.92 g, 2.0 mmol) in DMF (100 mL) were added HATU (0.84 g, 2.2 mmol) and DIPEA (0.38 mL, 2.2 mmol) dropwise at 0 °C. The reaction mixture was stirred for 3 h. Then, the solvent was removed, and the residue was extracted with ethyl acetate and an aqueous solution of citric acid (10%), a solution of NaHCO_3_ (5%), and brine. The crude product was purified via semipreparative HPLC, giving the desired product as a colorless oil (0.70 g, 42% yield). ESI-MS: calcd for C_45_H_61_N_6_O_9_, 830.02 [M + H]^+^; found, 829.90 [M + H]^+^. *T*_R_ = 21.47 min. ^1^H NMR (400 MHz, CDCl_3_): δ 7.39–7.31 (m, 18H), 4.77 (s, 6H), 3.69–3.67 (m, 6H), 3.25–3.23 (m, 6H), 2.46 (t, *J* = 6.7 Hz, 6H), 2.19 (t, *J* = 7.0 Hz, 6H), 1.97–1.91 (m, 6H), 1.85–1.70 (m, 6H). ^13^C NMR (CDCl_3_): δ 174.9, 173.8, 133.8, 129.3, 128.8, 44.2, 39.4, 33.0, 29.5, 23.7, 23.2.

#### Synthesis of 1,11,21-Trihydroxy-1,6,11,16,21,26-hexaazacyclotriacontane-2,7,12,17,22,27-hexaone (**11**, **H**_**3**_**L1**)

To a solution of the benzyl-protected derivative **10** (0.70 g, 0.84 mmol) in MeOH (30 mL) was added glacial acetic acid (1 mL). The mixture was treated with a catalytic amount (0.084 mmol) of palladium on activated charcoal (10% Pd basis) under a hydrogen atmosphere. After 24 h, the reaction mixture was filtered through Celite, concentrated under reduced pressure, diluted with water, and alkalinized with saturated sodium bicarbonate. The aqueous phase was extracted with ethyl acetate (4 × 10 mL), and the combined organic layers were dried over Na_2_SO_4_, filtered, and concentrated under vacuum. Compound **11** was obtained as a light yellow oil after preparative HPLC purification (0.43 g, 91% yield). ESI-MS: calcd for C_24_H_43_N_6_O_9_, 559.64 [M + H]^+^; found, 559.79 [M + H]^+^. *T*_R_ = 15.79 min. ^1^H NMR (400 MHz, CDCl_3_): δ 9.59 (bs, 3H), 7.83–7.81 (m, 3H), 3.53–3.38 (m, 6H), 3.15–2.93 (m, 6H), 2.40–2.24 (m, 5H), 2.20–2.16 (m, 2H), 2.02 (t, *J* = 7.1 Hz, 5H), 1.76–1.70 (m, 6H), 1.64–1.50 (m, 6H). ^13^C NMR (CDCl_3_): δ 174.5, 173.0, 172.2, 169.5, 158.9, 158.5, 129.9, 50.5, 47.3, 47.1, 38.7, 38.6, 33.0, 31.5, 31.2, 30.3, 29.7, 24.9, 24.8, 23.0, 22.3, 19.7. HR-ESI-MS *m*/*z* 559.30894; calcd for C_24_H_43_N_6_O_9_ ([M + H]^+^) 559.30860. Anal. Calcd for C_24_H_42_N_6_O_9_: C, 51.6; H, 7.6; N, 15.0. Found: C, 51.4; H, 7.5; N, 14.9.

#### Synthesis of Ethyl 17-(Benzyloxy)-10-(((benzyloxy)carbonyl)amino)-2,2-dimethyl-4,11,16-trioxo-3-oxa-5,12,17-triazahenicosan-21-oate (**13**)

Compound **13** was synthesized under the same coupling conditions used for compounds **4** and **9** bystarting from Z-Lys(Boc)-OH (1.05 g, 2.75 mmol) and the amino derivative **5** (1.09 g, 2.50 mmol). The desired product was obtained as a yellowish oil (1.40 g, 80% yield) after column chromatography. ESI-MS: calcd for C_36_H_53_N_4_O_9_, 685.84 [M + H]^+^; found, 685.73 [M + H]^+^. T_R_ = 26.16 min. ^1^H NMR (400 MHz, CDCl_3_): δ 7.37–7.35 (m, 5H), 7.31–7.26 (m, 5H), 6.99 (s, 1H), 5.85 (d, *J* = 7.7 Hz, 1H), 5.13–4.96 (m, 2H), 4.76 (s, 2H), 4.07 (q, *J* = 7.1 Hz, 2H), 3.73–3.59 (m, 2H), 3.28–3.13 (m, 2H), 3.03–2.97 (m, 2H), 2.47–2.40 (m, 2H), 2.28 (t, *J* = 7.3 Hz, 2H), 1.94–1.88 (m, 2H), 1.84–1.69 (m, 3H), 1.62–1.58 (m, 1H), 1.39 (s, 11H), 1.31 (dd, *J* = 19.1, 12.2 Hz, 2H), 1.19 (t, *J* = 7.1 Hz, 3H). ^13^C NMR (CDCl_3_): δ 13C NMR (101 MHz, cdcl3) δ 174.3, 172.9, 172.0, 156.2, 136.2, 134.1, 129.2, 128.9, 128.6, 128.4, 128.0, 127.9, 78.9, 76.2, 66.8, 60.4, 54.8, 44.4, 39.9, 39.2, 38.5, 32.2, 31.2, 29.7, 29.4, 28.3, 23.7, 22.4, 22.1, 14.1.

#### Synthesis of Ethyl 17,27,37,47-Tetrakis(benzyloxy)-10-(((benzyloxy)carbonyl)amino)-2,2-dimethyl-4,11,16,21,26,31,36,41,46-nonaoxo-3-oxa-5,12,17,22,27,32,37,42,47-nonaazahenpentacontan-51-oate (**15**)

The tetramer **15** was synthesized under the same coupling conditions used for compounds **4**, **9**, and **13** by starting from the acid derivative **14** (0.61 g, 0.93 mmol) and the amino derivative **12** (0.90 g, 1.02 mmol). The desired product was obtained as a colorless oil (0.97 g, 69% yield) after column chromatography. ESI-MS: calcd for C_81_H_113_N_10_O_18_, 1514.85 [M + H]^+^; found, 1514.16 [M + H]^+^, 775.95 [M + 2H]^2+^. *T*_R_ = 25.88 min. ^1^H NMR (400 MHz, CDCl_3_): δ 7.47–7.28 (m, 25H), 5.13–4.96 (m, 2H), 4.86–4.65 (m, 8H), 4.09 (dd, *J* = 13.8, 6.8 Hz, 3H), 3.80–3.53 (m, 8H), 3.32–3.10 (m, 7H), 3.04–3.00 (m, 2H), 2.54–2.34 (m, 7H), 2.29 (t, *J* = 7.0 Hz, 2H), 2.23–2.05 (m, 6H), 1.96–1.90 (m, 8H), 1.82–1.68 (m, 8H), 1.66–1.53 (m, 2H), 1.40 (s, 12H), 1.25–1.19 (m, 4H), 0.94–0.85 (m, 2H). ^13^C NMR (CDCl_3_): δ 174.5, 172.9, 136.2, 134.0, 129.2, 129.1, 128.8, 128.5, 128.2, 128.0, 67.0, 60.5, 55.0, 44.1, 39.4, 39.0, 32.9, 32.0, 31.3, 29.8, 29.7, 29.5, 28.4, 23.9, 23.0, 22.5, 22.2, 14.2.

#### Synthesis of Benzyl (6,16,26,36-Tetrakis(benzyloxy)-2,7,12,17,22,27,32,37,42-nonaoxo-1,6,11,16,21,26,31,36,41-nonaazacycloheptatetracontan-43-yl)carbamate (**16**)

Compound **15** was Boc-deprotected as described for **3**. Then, the ethyl group was hydrolyzed by LiOH as for **6**. To a dilute solution of the fully deprotected tetramer (0.55 g, 0.367 mmol) in DMF (40 mL) were added HATU (0.154 g, 0.40 mmol) and DIPEA (0.07 mL, 0.40 mmol) dropwise at 0 °C. The reaction mixture was stirred for 3 h. Then, the solvent was removed, and the residue was extracted with ethyl acetate and an aqueous solution of citric acid (10%), a solution of NaHCO_3_ (5%), and brine. The crude product was purified via semipreparative HPLC, giving the desired product as a colorless oil (0.29 g, 57% yield). ESI-MS: calcd for C_74_H_99_N_10_O_15_, 1368.66 [M + H]^+^; found, 1368.36 [M + H]^+^, 684.74 [M+2H]^2+^. *T*_R_ = 25.73 min. ^1^H NMR (400 MHz, DMSO-*d*_6_): δ 7.96–7.63 (m, 6H), 7.56–7.13 (m, 25H), 5.06–4.89 (m, 2H), 4.85–4.71 (m, 8H), 4.00–3.76 (m, 3H), 3.09–2.88 (m, 10H), 2.84–2.61 (m, 1H), 2.43–2.25 (m, 8H), 2.09–1.93 (m, 9H), 1.75–1.71 (m, 9H), 1.59–1.53 (m, 8H), 1.39–1.08 (m, 7H), 1.07–0.76 (m, 2H). ^13^C NMR (DMSO-*d*_6_): δ 171.8, 156.4, 135.2, 129.8, 129.1, 128.9, 128.7, 128.2, 128.1, 75.7, 65.8, 55.1, 44.5, 38.5, 33.0, 29.5, 24.7, 23.2.

#### Synthesis of (*S*)-43-Amino-6,16,26,36-tetrahydroxy-1,6,11,16,21,26,31,36,41-nonaazacycloheptatetracontane-2,7,12,17,22,27,32,37,42-nonaone (**17**, **H**_**4**_**L2**)

Compound **17** was synthesized as previously described for **11** by starting from derivative **16** (0.13 g, 71% yield). ESI-MS: calcd for C_38_H_69_N_10_O_13_, 874.03 [M + H]^+^; found, 873.75 [M + H]^+^. *T*_R_ = 21.28 min. ^1^H NMR (400 MHz, DMSO-*d*_6_): δ 9.64–9.59 (m, 3H), 8.07–8.04 (m, 2H), 7.86–7.71 (m, 4H), 3.47–3.44 (m, 9H), 3.04–2.99 (m, 10H), 2.42–2.22 (m, 8H), 2.04–2.00 (m, 8H), 1.76–1.49 (m, 20H), 1.47–1.16 (m, 5H). ^13^C NMR (DMSO-*d*_6_): δ 172.9, 172.2, 168.7, 158., 56.5, 52.7, 47.2, 38.7, 33.0, 31.2, 29.7, 29.1, 24.8, 24.5, 23.0, 22.1. HR-ESI-MS *m*/*z* 873.50488; calcd for C_38_H_69_N_10_O_13_ ([M + H]^+^) 873.50401. Anal. Calcd for C_38_H_68_N_10_O_13_: C, 52.3; H, 7.9; N, 16.0. Found: C, 52.3; H, 7.8; N, 16.1.

### Thermodynamic Solution Studies

#### General Considerations

Unless otherwise stated, all commercially available reagents and solvents were of analytical grade, were purchased from commercial suppliers (Sigma-Aldrich, Titripur, Merck, Fisher Scientific, Fluka), and were used as received without further purification. All solutions were prepared in doubly distilled water. A stock solution of Fe(III) was prepared immediately before use from Fe(ClO_4_)_3_·*x*H_2_O in 0.01 M HClO_4_ and standardized by an inductively coupled plasma–optical emission spectrometer (ICP-OES; iCAP 7400 Duo ICP-OES) along with spectrophotometric determination, on the basis of the molar extinction coefficient ε = 4160 M^–1^ cm^–1^ at 240 nm.^70,71^ Stock solutions of Ga(III) and Zr(IV) were prepared immediately before use from Ga(ClO_4_)_3_·*x*H_2_O and anhydrous ZrCl_4_, respectively, in 0.1 M HClO_4_ to prevent hydrolysis and standardized by ICP-OES (iCAP 7400 Duo ICP-OES) along with direct titration with ethylenediaminetetraacetic acid (EDTA).^72,73^ The HClO_4_ solutions were titrated with standardized NaOH (0.1 N). The carbonate-free NaOH solution was standardized by titration with potassium hydrogen phthalate (KHP). All stock solutions were prepared using a R200D Sartorius analytical balance (with 0.01 mg precision).

All measurements were performed at 0.1 M NaClO_4_ ionic strength, which was chosen instead of 1.0 M NaClO_4_ ionic strength in order to increase the solubility of the investigated ligands and their complexes. We are aware that some measurements were performed at a very acidic pH (<1), where the ionic strength 0.1 M is not enough to keep the ionic activity stable, but due to the decomposition of hydroxamate ligands in strong acids,^43,51^ all measurements performed below pH 1 were assumed to be endowed with a large error and (i) were not taken into account during data evaluation or (ii) precluded from the discussion.

#### Electrospray Ionization Mass Spectrometry (ESI-MS)

ESI**-**MS data were recorded on a Bruker Q-FTMS spectrometer. The instrumental parameters were as follows: scan range, *m*/*z* 200–1600; dry gas, nitrogen; temperature, 170 °C; capillary voltage, 4500 V; ion energy, 5 eV. The capillary voltage was optimized to the highest signal to noise ratio. The spectra were recorded in the positive mode. Compounds were dissolved in a MeOH/H_2_O solution (80/20 by weight); the same solvent mixture was used to dilute the matrix solutions to the concentration range of 0.01 mM. The Fe(III), Ga(III), and Zr(IV) and stock solutions were prepared as described previously and added to the ligand solutions in 1/1, 2/1 and 1/3 mixtures for Fe(III) and Ga(III) and 1/1 and 1/3 mixtures for Zr(IV), all at pH 3 (the pH was adjusted by using acetic acid). The free hydrogen ion concentration was measured with a Mettler-Toledo InLab Semi-Micro combined glass electrode filled with NaCl in MeOH/H_2_O (80/20 by weight). Potential differences were measured with a Beckman ϕ72 pH meter, standardized according to the classical methods with buffers prepared according to reported procedures in MeOH/H_2_O solvent (80/20 by weight).^74,75^

#### Potentiometric Titrations

The potentiometric titrations of ligands and their complexes were carried out using a Titrando 905 (Metrohm) automatic titrator system, equipped with a combined glass electrode (Mettler Toledo, InLab Semi-Micro, with XEROLYT EXTRA Polymer filling) and a 800 Dosino dosing system, equipped with a a 2 mL micro buret. The ionic strength was fixed at *I* = 0.1 M with NaClO_4_. The electrode was calibrated daily in terms of hydrogen ion concentration using HClO_4_ (0.1 M) with CO_2_-free NaOH solutions (0.1 M).^76^ A stream of high-purity argon, presaturated with water vapor, was passed over the surface of the solution cell, the cell was filled with 50 mL of the studied solution, and the system was thermostated at 25.0 ± 0.2 °C. At least three titrations were performed for each system, with a starting concentration of the ligand of 1 mM and a 1:1 metal to ligand molar ratio with a 10% excess of the ligand in the pH range 2–11. The purity and exact concentration of the ligand solutions were determined using the Gran method.^77^ Special care was taken to ensure that complete equilibration was attained. The titration curves were carefully checked and did not display any pH fluctuations that often accompany the precipitation of metal hydroxides. The potentiometric data were refined with the SUPERQUAD^78^ and HYPERQUAD^79^ programs, which use nonlinear least-squares methods. The successive protonation constants of the ligand were calculated from the cumulative constants determined with the program and defined by eqs 1 and 2 (charges are omitted for clarity).
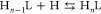
1
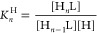
2

The stability constants calculated for metal complexes are defined by eqs 3 and 4:
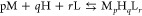
3
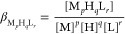
4

The uncertainties in the log *K* values correspond to the added standard deviations in the cumulative constants.

#### pH-Dependent UV–Vis Titrations

The pH-dependent UV–vis spectrophotometric experiments for the Fe(III)-**H**_**3**_**L1**, Fe(III)-**H**_**4**_**L2**, Ga(III)-**H**_**3**_**L1**, Ga(III)-**H**_**4**_**L2**, Ga(III)-**H**_**3**_**L4**, Zr(IV)-**H**_**3**_**L1**, Zr(IV)-**H**_**4**_**L2**, Zr(IV)-**H**_**3**_**L3**, and Zr(IV)-**H**_**3**_**L4** systems were carried out as a function of concentration with a Varian Cary 300 Bio spectrophotometer in the 300–700 nm range for iron complexes and 200–300 nm range for gallium and zirconium solutions using Hellma quartz optical cells with a 1 cm path length. To calculate the stability constants for investigated systems, two sets of pH-dependent UV–vis titrations were carried out: in the pH ranges (i) 0.1–2 and (ii) 2–11. In the (i) series, the experiments were performed by making 20 samples, differing by 0.1 pH unit, with a constant total volume of 0.7 mL and concentration of metal ion of ∼0.05–0.1 mM and metal to ligand molar ratio of 1:1; for all samples, the ionic strength was adjusted to 0.1 M by the addition of NaClO_4_ and the pH (range 0.1–2.0) was controlled by the concentration of HClO_4_. After preparation, each solution was allowed to equilibrate for about 1 h, and then its UV–vis spectrum was recorded. This was necessary to minimize the effects of hydroxamate ligand hydrolysis, which occurs in strong acid.^43,51^ In the (ii) set of experiments, 3 mL of a solution containing a 1:1 Fe(III):ligand molar ratio, where the ferric concentration was around 0.20 mM, was introduced into a cell and the pH was adjusted by adding the proper microvolume of HClO_4_; the solutions were allowed to equilibrate (up to 30 min) and checked with a Mettler Toledo Super Easy pH meter with an accuracy of ±0.01, and then the spectra were recorded.

#### Metal Competition Batch UV–Vis Titrations

In order to calculate the stability constants of the investigated complexes, several competitive titrations were performed. All of them were carried out as a function of concentration with a Varian Cary 300 Bio spectrophotometer in the 300–650 nm range (with 1 nm precision) using Hellma quartz optical cells with a 1 cm path length. Spectrophotometric titrations were performed on samples with a concentration of the ligand of ∼0.05–0.1 mM and *I* = 0.1 M (completed by adding NaClO_4_), at 25.0 ± 0.1 °C; pH 1.5 or 2.0 was adjusted by adding the proper volume of HClO_4_. In general, the stock solution of the starting complex (Fe(III)-L, Ga(III)-L, or Zr(IV)-L, respectively) was divided into several aliquots to which an excess of titrant (Ga(III), Fe(III), or Zr(IV)-NTA, respectively) was added. After preparation, each solution was allowed to equilibrate for about 1 h, and then its UV–vis spectrum was recorded. The vials were kept in the dark, and the absorbance was measured again after 24, 48, and 120 h. Changes were observed only between the spectra collected after 1 and 24 h, indicating that an equilibrium was attained.

In order to determine the log β values of [GaH**L**]^+^ for **H**_**3**_**L1** and **H**_**3**_**L4** and of [GaH_2_**L2**]^+^, competition experiments at pH 1.5 of (i) Fe(III)-**H**_**3**_**L** + Ga(III) and (ii) Ga(III)-**L** + Fe(III) were performed. For (i) 15 samples with a constant concentration of Fe(III) ions and **L** (1:1) were titrated by up to 600 equiv of Ga(III) ions; for Ga(III)-**L** + Fe(III), 18 samples with a constant concentration of Ga(III) ions and **H**_**3**_**L** (1:1) were titrated by up to 4 equiv of Fe(III) ions.

In order to determine the log β values of [Zr**L**]^+^ for **H**_**3**_**L1**, **H**_**4**_**L2**, **H**_**3**_**L3**, **H**_**3**_**L4**, and **DFOE** and for [ZrH**L2**]^+^, competition experiments at pH 1.5 or 2, Fe(III)-**L** + Zr(IV)-NTA, were performed. In each experiment 18 samples with a constant concentration of Fe(III) ions and ligands were titrated by up to 50 equiv of a Zr(IV)-NTA solution with a metal to ligand molar ratio of 1:3, starting from 0 equiv.

#### Data Treatment

In the calculations of complex stability constants, the protonation constants of free ligands (Table 1) and the constants were related to hydrolytic species being taken into account: Ga(III),^60^ Ga(OH)^2+^ log β_GaH_–1__ = −3.11, Ga(OH)_2_^+^ log β_GaH_–2__ = −7.66, Ga(OH)_3_ log β_GaH_–3__ = −11.94, Ga(OH)_4_^–^ logβ_GaH_–4__ = −15.66, to Fe(III),^80^ Fe(OH)^2+^ log β_FeH_–1__ = −2.56, Fe(OH)_2_^+^ log β_FeH_–2__= −6.2, Fe(OH)_3_ log β_FeH_–3__ = −11.44, Fe(OH)_4_^–^ log β_FeH_–4__ = −21.88, Fe(OH)_5_^2–^ log β_FeH_–5__ = −2.74, Fe(OH)_6_^3–^, and Zr(IV),^60^ Zr(OH)^3+^ log β_ZrH_–1__ = −0.56, Zr(OH)_2_^2+^ log β_ZrH_–2__ = −1.44, Zr(OH)_4_ log β_ZrH_–4__ = −8.85, Zr(OH)_6_^2–^ log β_ZrH_–6__ = −30.6, Zr_3_(OH)_4_^8+^ log β_Zr_3_H_–4__ = −6.96, Zr_4_(OH)_8_^8+^ log β_Zr_4_H_–8__ = 6.52, Zr_3_(OH)_9_^3+^ log β_Zr_3_H_–9__ = 12.19. The Zr(IV) hydrolysis constants for the species Zr(OH)^3+^, Zr(OH)_2_^2+^, Zr(OH)_4_, and Zr_3_(OH)_4_^8+^ were recalculated for 0.1 M NaClO_4_ ionic strength according to literature parameters.^60^ The p*K*_w_ value used in the calculation at the 0.1 M NaClO_4_ ionic strength was −13.77.^81^

The UV–vis data were refined to obtain the overall binding constant using SPECFIT/32 software^82−84^ that adjusts the absorptivity and the stability constants of the species formed at equilibrium. Specfit uses factor analysis to reduce the absorbance matrix and to extract the eigenvalues prior to the multiwavelength fit of the reduced data set according to the Marquardt algorithm.^82−84^ Uncertainties in log β were calculated from the standard deviation.

The competition data were refined to obtain the overall binding constant using SPECFIT/32 software.^82−84^ The protonation constants of ligands and formation constants for iron complexes (Table 1 and the literature^24^) were used as fixed parameters during data analysis. The concentration of iron complexes was calculated from the absorbance spectra (collected in the 300–700 nm range). Hydrolytic forms of the ferric ion in the studied pH range are characterized by an absorption band with a λ_max_ value of below 300 nm, and therefore they are beyond the experimental wavelength window. However, the spectrum of Fe(III) in solution at the pH of the experiment was fixed in the calculations.^85,86^ The stability constants of the Fe(III)-NTA^48^ and Zr(IV)-NTA^27^ complexes were taken from the literature and were used as fixed constants during the evaluation of the stability constants of the zirconium complexes.

The competition equilibrium is described by eqs 5 and 6:
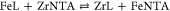
5
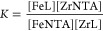
6

The molecular charges are omitted for clarity. The data were processed using Origin 7.0. The species distribution diagrams were computed with the HYSS program.^79^

## Supporting Information Available

ESI-MS spectra and UV–vis spectroscopic data from solution studies, detail of the Fe(III) complex solution study, and ^1^H/^13^C NMR spectra and chromatograms of the synthesized compounds (PDF)
