# Supplementary material for: Thermodynamic Stability and Speciation of Ga(III) and Zr(IV) Complexes with High-Denticity Hydroxamate Chelators
Source: Inorg Chem. 2021 Aug 20;60(17):13332–47. doi: 10.1021/acs.inorgchem.1c01622 (PMC8424644; doi:10.1021/acs.inorgchem.1c01622)
Supplement: Supplementary file 1 — ic1c01622_si_001.pdf [file ic1c01622_si_001.pdf]

Supporting information for:

Thermodynamic Stability and Speciation of Ga(III) and Zr(IV) Complexes with High-Denticity Hydroxamate Chelators

Yuliya Toporivska,[a] Andrzej Mular,[a] Karolina Piasta,[a] Małgorzata Ostrowska,[a] Davide Illuminati,[b] Andrea Baldi,[b] Valentina Albanese,[b] Salvatore Pacifico,[b] Igor O. Fritsky,[c] Maurizio Remelli,[b] Remo Guerrini[b] and Elzbieta Gumienna-Kontecka[a]*

[a)] *University of Wroclaw, Faculty of Chemistry, 14 F. Joliot-Curie, 50-383 WROCLAW, Poland*

[b)] *University of Ferrara, Dipartimento di Scienze Chimiche, Farmaceutiche ed Agrarie, 46 Via Luigi Borsari, 44121 FERRARA, Italy*

[c)] *Taras Shevchenko National University of Kyiv, Department of Chemistry, 64 Volodymyrska Str., 01601 KYIV, Ukraine*

e-mail: elzbieta.gumienna-kontecka@chem.uni.wroc.pl

Ligand protonation constants

a) b)

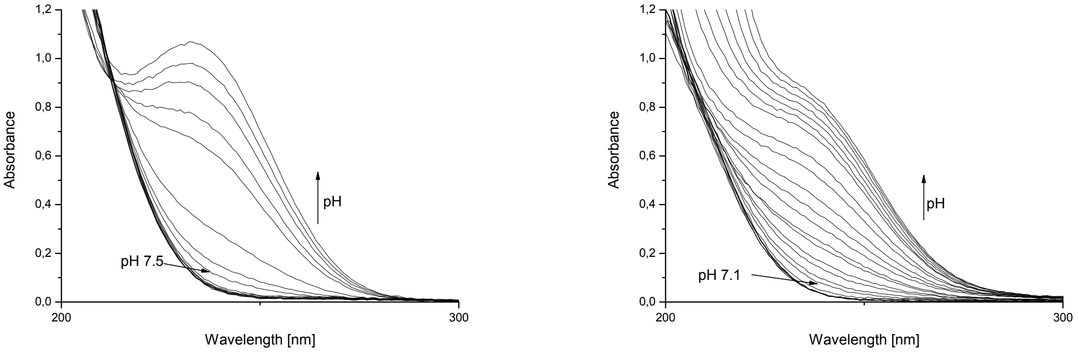

Fig. S1. UV-Vis titration of $\mathbf{H_3L1}$ in pH range 1.0 -11, $c_{\mathbf{L1}}$ = 0.045 mM (a) and $\mathbf{H_4L2}$ in pH range 1.0 -11, $c_{\mathbf{L2}}$ = 0.05 mM (b), I = 0.1 M $NaClO_4$, T = 25°C.

a) b)

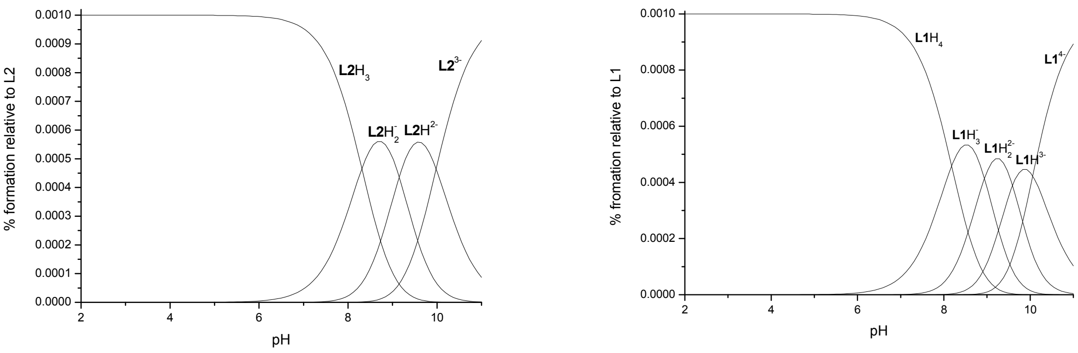

Fig. S2. Calculated species distribution diagram for $\mathbf{H_3L1}$ (a) and $\mathbf{H_4L2}$ (b), at fixed concentration of the ligands: c_L=1 mM; log β and pK values for $\mathbf{H_3L1}$ and $\mathbf{H_4L2}$ are given in Table 1.

ESI-MS results: stoichiometry evaluation

The spectra were collected for M-ligand solutions in metal-to-ligand molar ratios of 1:1, 2:1 and 1:3 for Fe(III) and Ga(III), 1:1 and 1:2 for Zr(IV), all at pH 3. The spectra collected for the same M-L system differed only slightly by intensity that is why here we show only the spectra for 1:1 metal-to-ligand molar ratio.

a)

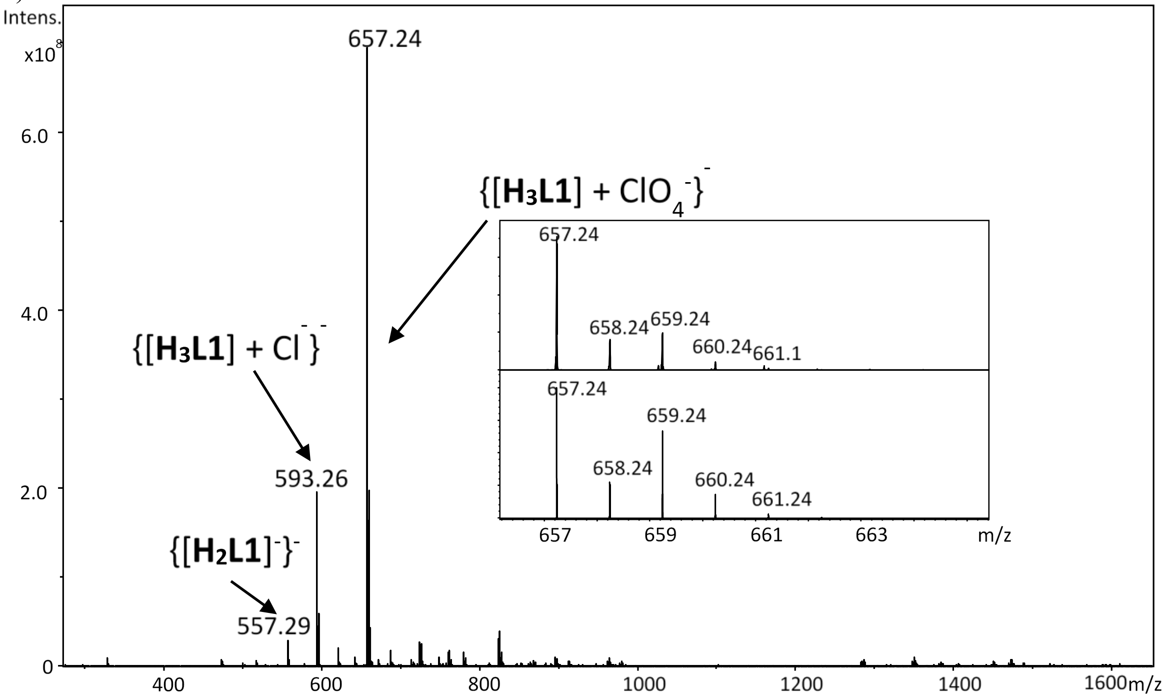

b)

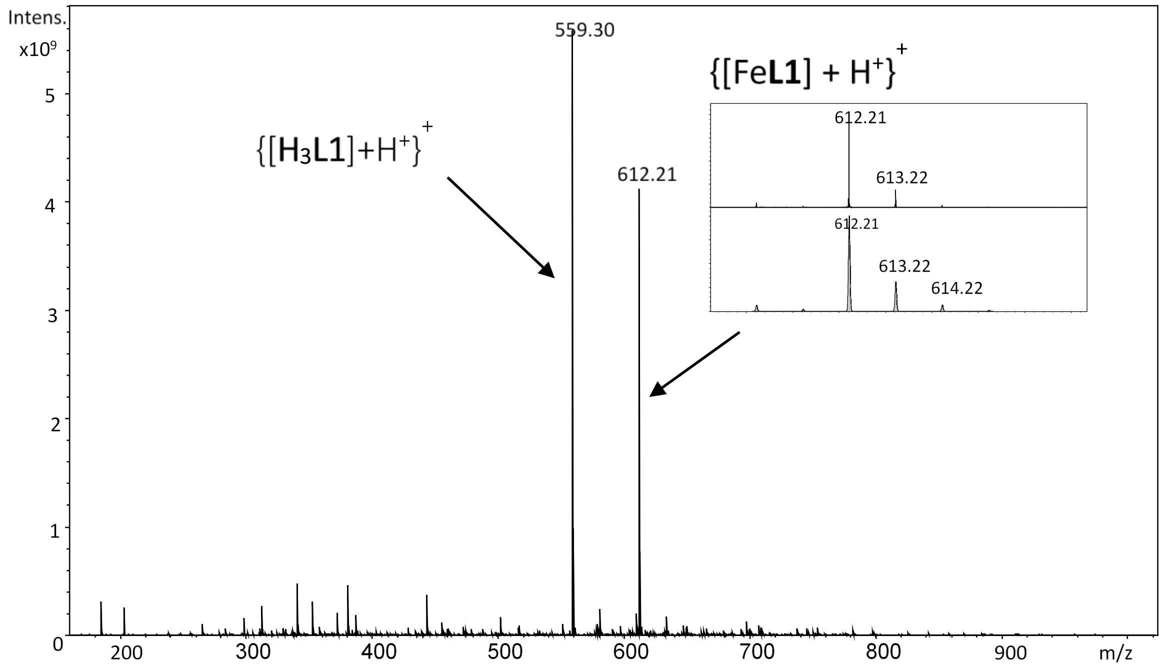
Intens.
x10^9
559.30
612.21
{[H3L1]+H+}+
{[FeL1] + H+}+
612.21
613.22
612.21
613.22
614.22
m/z

c)

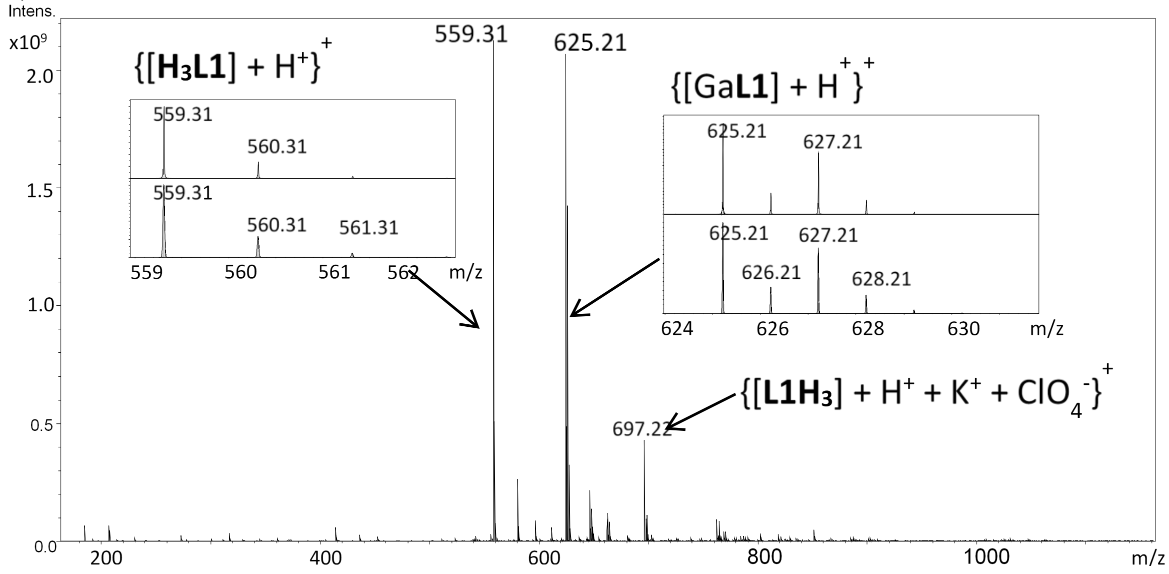
Intens.
x10^9
559.31
625.21
{[H3L1] + H+}+
559.31
560.31
559.31
560.31
561.31
{[GaL1] + H+}+
625.21
627.21
625.21
626.21
627.21
628.21
{[L1H3] + H+ + K+ + ClO4-}+
697.22
m/z

d)

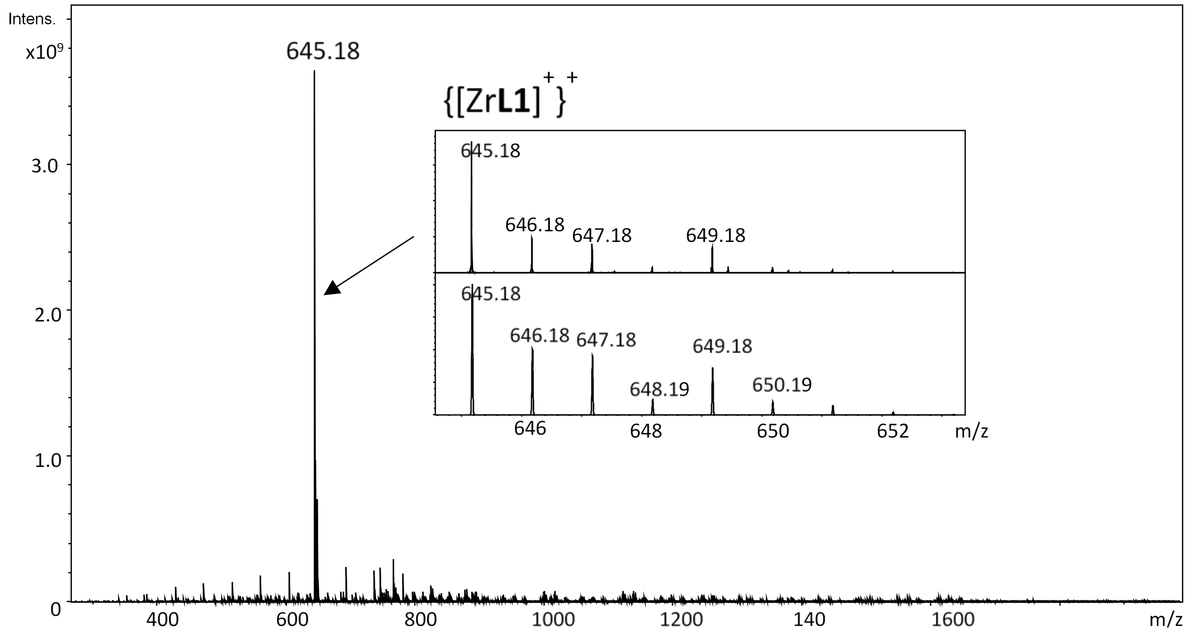
Intens.
x10⁹
645.18
{[ZrL1]+ +}
645.18
646.18
647.18
649.18
645.18
646.18
647.18
648.19
649.18
650.19
646
648
650
652
m/z
3.0
2.0
1.0
0
400
600
800
1000
1200
140
1600
m/z

e)

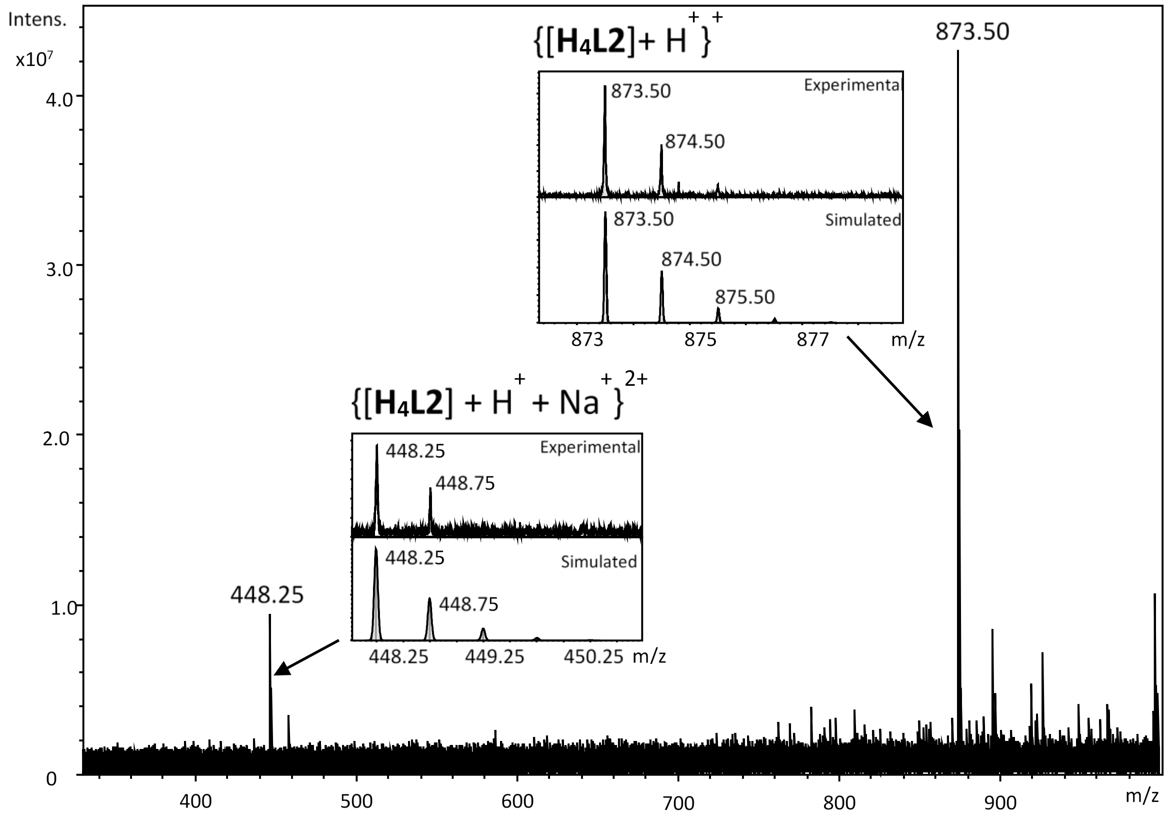
Intens.
x10⁷
{[H4L2]+ H+}+
873.50
873.50
Experimental
874.50
873.50
Simulated
874.50
875.50
873
875
877
m/z
{[H4L2] + H+ + Na+}2+
448.25
Experimental
448.75
448.25
Simulated
448.75
448.25
449.25
450.25
m/z
448.25
4.0
3.0
2.0
1.0
0
400
500
600
700
800
900
m/z

f)

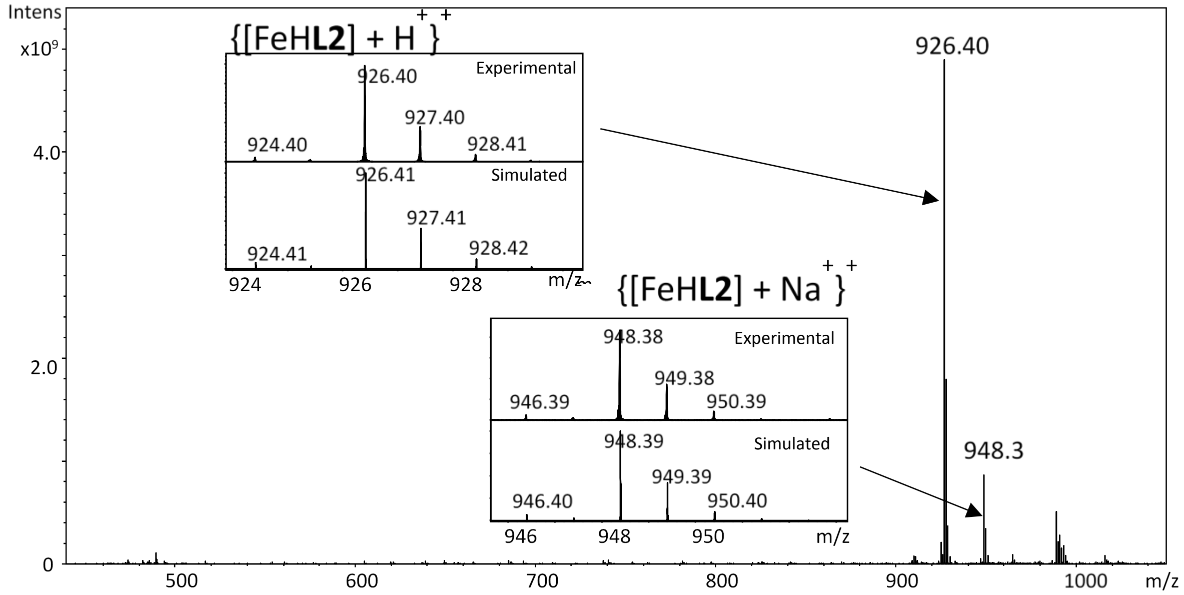
Intens
x10^9
4.0
2.0
0
500
600
700
800
900
1000
m/z
926.40
948.3
{[FeHL2] + H+}+
Experimental
926.40
927.40
924.40
928.41
Simulated
926.41
927.41
924.41
928.42
924
926
928
m/z
{[FeHL2] + Na+}+
Experimental
948.38
949.38
946.39
950.39
Simulated
948.39
949.39
946.40
950.40
946
948
950
m/z

g)

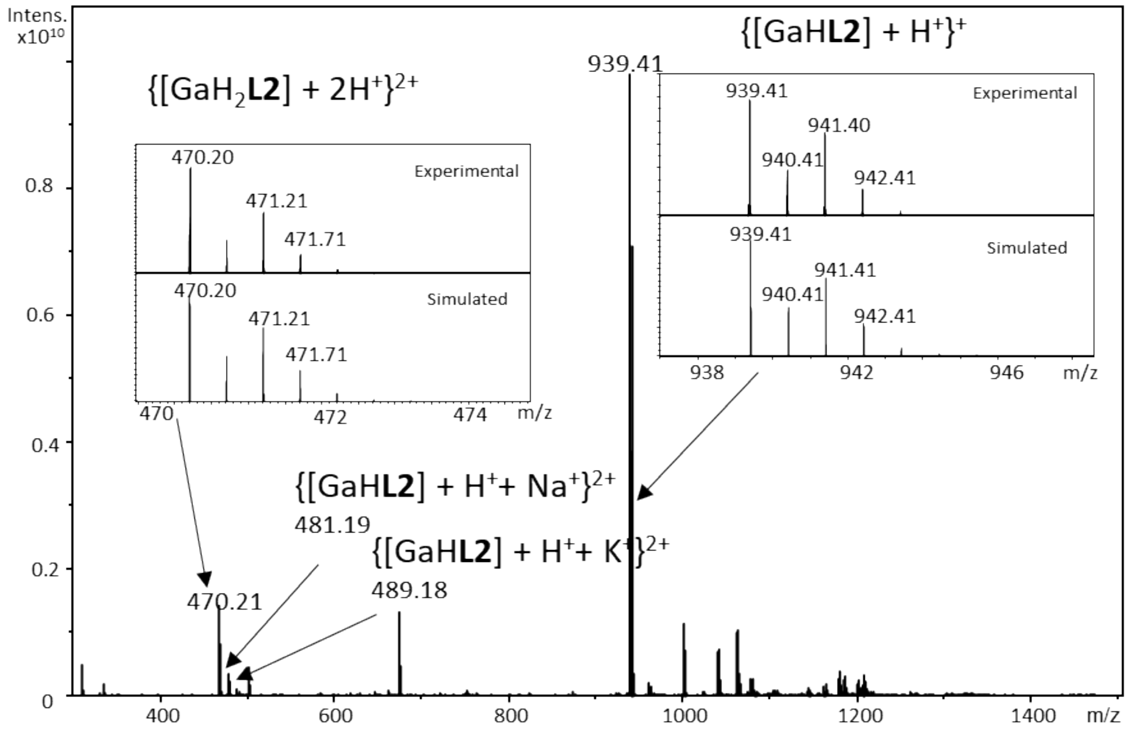
Intens.
x10^10
0.8
0.6
0.4
0.2
0
400
600
800
1000
1200
1400
m/z
939.41
{[GaHL2] + H+}+
Experimental
939.41
941.40
940.41
942.41
Simulated
939.41
941.41
940.41
942.41
938
942
946
m/z
{[GaH2L2] + 2H+}2+
Experimental
470.20
471.21
471.71
Simulated
470.20
471.21
471.71
470
472
474
m/z
{[GaHL2] + H++ Na+}2+
481.19
{[GaHL2] + H++ K+}2+
489.18
470.21

h)

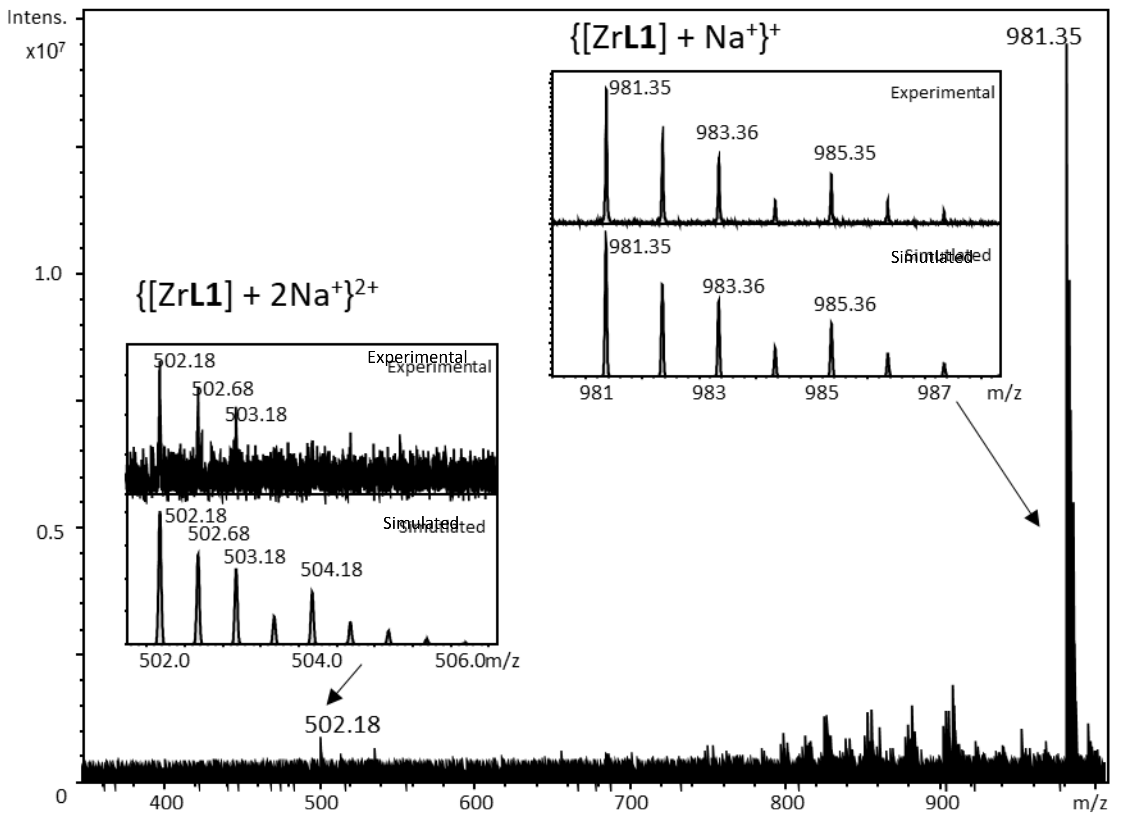

Intens.
x10^7
1.0
0.5
0
{[ZrL1] + Na+}+
981.35
Experimental
983.36
985.35
Simulated
985.36
981
983
985
987
m/z
{[ZrL1] + 2Na+}2+
502.18
502.68
503.18
504.18
502.0
504.0
506.0m/z
400
500
600
700
800
900

i)

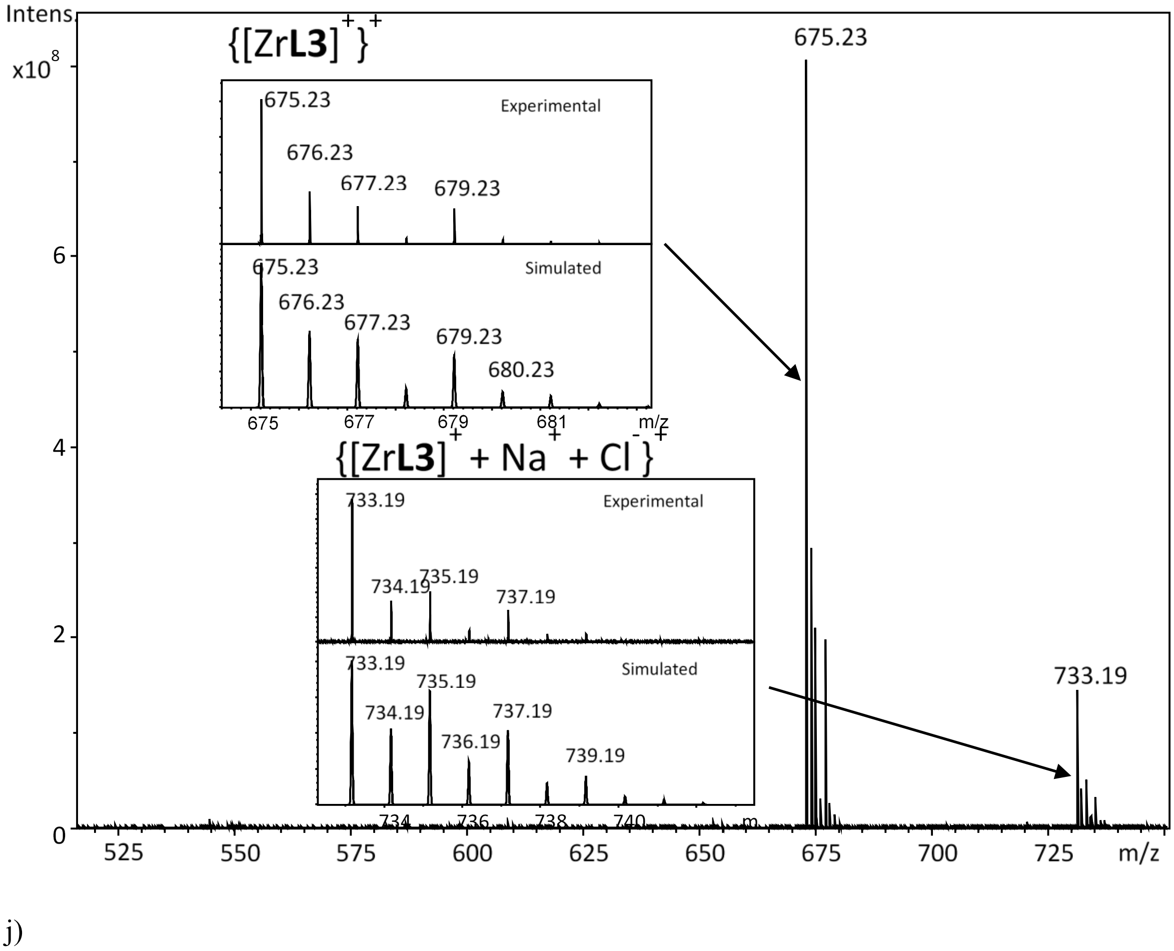

Intens.
x10^8
6
4
2
0
{[ZrL3]+}+
675.23
Experimental
676.23
677.23
679.23
Simulated
680.23
675
677
679
681
{[ZrL3]+ + Na+ + Cl-}+
733.19
734.19
735.19
736.19
737.19
739.19
734
736
738
740
525
550
575
600
625
650
675
700
725
m/z

j)

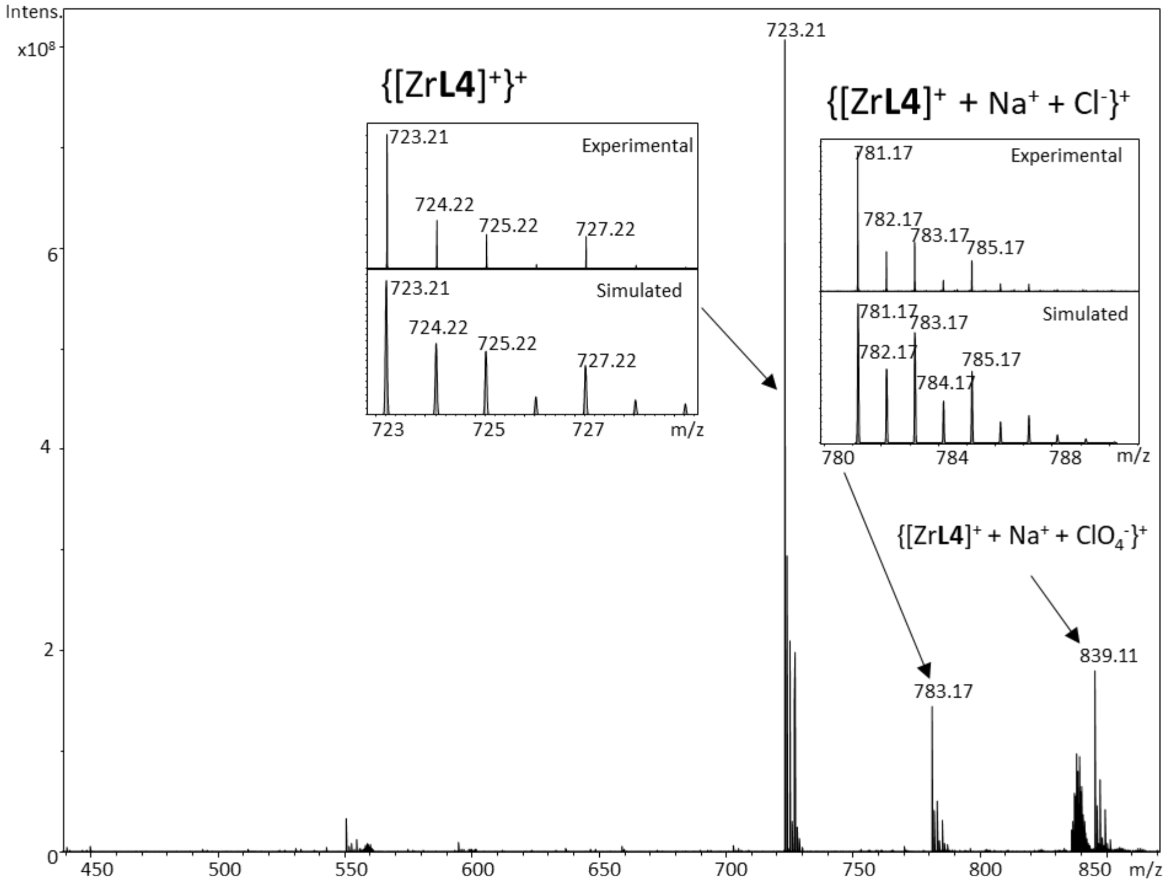

Fig. S3. ESI-MS spectra for **H_3L1** (a) and H_4L2 (e) at pH 3; and for Fe(III)-**H_3L1** (b), Ga(III)-**H_3L1** (c), Zr(IV)-**H_3L1** (d), Fe(III)-**H_4L2** (f), Ga(III)-**H_4L2** (g), Zr(IV)-**H_4L2** (h), Zr(IV)-**H_3L3** (i) and Zr(IV)-**H_3L4** (j) solutions all in metal-to-ligand molar ratio 1:1 at pH 3. The spectra were collected in MeOH/H_2O 80/20 by weight mixture in positive mode.

The ligand **H_3L1** is characterized by the signals at 557.29, 593.26 and 657.24 *m/z*, corresponding to {[H_2**L1**]$^-$}$^-$ and adducts with the Cl^- and ClO_4^- ions (Fig. S3a). The peaks corresponding to ferric complexes of **H_3L1** were 612.22, 634.21 and 650.18 *m/z*, corresponding to mononuclear complex {[Fe**L1**] + H^+}$^+$ and its adducts with Na^+ and K^+ ions (Fig. S3b). The Ga(III)-**H_3L1** spectra were characterized by the presence of three peaks, corresponding to the mononuclear complex {[Ga**L1**] + H^+}$^+$ and its adducts with Na^+ and K^+ ions (625.21, 647.20 and 663.17 *m/z*, respectively; Fig. S3c). The Zr(IV)-**H_3L1** spectra showed single peak at 645.18 *m/z* corresponding to the {[Zr**L1**]$^+$}$^+$ complex (Fig. S3d).

The ligand **H_4L2** is characterized by the signals at 448.25 and 873.50 *m/z*, corresponding to {[H_4**L2**] + H^+ + Na^+}$^{2+}$ and {[H_4**L2**] + H^+}$^+$ forms, respectively (Fig. S3e). The ESI-MS spectra of ferric complexes of **H_3L1** shows one major peak corresponding to the mononuclear {[FeH**L2**] + H^+}$^+$ complex (926.40 *m/z*), with other noticeable peaks on the spectrum corresponding to its adduct with Na^+ ion (948.38 *m/z*) (Fig. S3f). On the spectra collected for Ga(III)-**H_4L2** system, the peaks 470.21 and 939.40 *m/z* are clearly visible and correspond to the {[GaH**L2**] + $2H^+$}$^{2+}$ and {[GaH**L2**] + H^+}$^+$ complexes; its adducts with Na^+ and K^+ (peaks 481.19 and 489.18 *m/z*, respectively) were observed less intense in

~~on~~ the spectra (Fig. S3g). The Zr(IV)-**H_4L2** spectra were characterized by the presence of two peaks 981.35 and 502.18 *m/z* corresponding to mononuclear complexes $\{[Zr\mathbf{L2}] + Na^+\}^+$ and $\{[Zr\mathbf{L2}] + 2Na^+\}^{2+}$ (Fig. S3h).

In the spectra collected for Zr(IV) complexes with **H_3L3** and **H_3L4**, only signals corresponding to mononuclear species were found, with 684.23 *m/z* peak corresponding to $\{[Zr\mathbf{L3}]^+\}^+$ (Fig. S3i) and 723.21 *m/z* for $\{[Zr\mathbf{L4}]^+\}^+$ (Fig. S3j).

Table S1. Intensity maxima of major complexes observed by ESI-MS[a]

Complex	Pseudomolecular ion	m/z experimental	m/z simulated
Fe(III)-H_3L1	$\{[FeH\mathbf{L1}]\}^+$	612.21	612.21
Ga(III)-H_3L1	$\{[\mathbf{H_4L1}]^+\}^+$	559.31	559.31
	$\{[Ga\mathbf{L1}] + H^+\}^+$	625.21	625.21
Zr(IV)-H_3L1	$\{[Zr\mathbf{L1}]^+\}^+$	645.18	645.18
Fe(III)-H_4L2	$\{[FeH\mathbf{L2}] + H^+\}^+$	926.40	926.41
	$\{[FeH\mathbf{L2}] + Na^+\}^+$	948.38	348.39
Ga(III)-H_4L2	$\{[GaH\mathbf{L2}] + 2H^+\}^{2+}$	470.20	470.20
	$\{[GaH\mathbf{L2}] + H^+ + Na^+\}^{2+}$	481.19	481.20
	$\{[GaH\mathbf{L2}] + H^+ + K^+\}^{2+}$	489.18	489.18
	$\{[GaH\mathbf{L2}] + H^+\}^+$	939.41	939.41
Zr(IV)-H_4L2	$\{[Zr\mathbf{L2}] + 2Na^+\}^{2+}$	502.18	502.18
	$\{[Zr\mathbf{L2}] + Na^+\}^+$	981.35	981.35
Zr(IV)-H_3L3	$\{[Zr\mathbf{L3}]^+\}^+$	675.23	675.23
	$\{[Zr\mathbf{L3}]^+ + Na^+ + Cl^-\}^+$	733.19	733.19
Zr(IV)-H_3L4	$\{[Zr\mathbf{L4}]^+\}^+$	723.27	723.27
	$\{[Zr\mathbf{L4}]^+ + Na^+ + Cl^-\}^+$	783.17	783.17
	$\{[Zr\mathbf{L4}]^+ + Na^+ + ClO_4^-\}^+$	839.11	839.11

[a] MeOH/H_2O 80/20 by weight mixture in positive mode.

Fe(III) complex formation equilibria

It is well known, that ferric hydroxamate complex formation starts at very low pH,[1-3] and as the pH-metric method cannot be applied at very acidic pH, spectrophotometric measurements over two different pH ranges, i.e. 1.0 - 2.0 and 2.0-11, were also performed. The experimental protocol was similar to that of previously described by us.[1,4] To identify specific complex form from the UV-Vis spectra, the relationship between the spectroscopic parameters of ligand-to-metal charge transfer

(LMCT) band of a complex and the number of hydroxamates bound to ferric ion was used[5]. The coordination of one hydroxamate group results in $\varepsilon_{510} \sim$ 1000 $M^{-1}cm^{-1}$, when two and three lead to $\varepsilon_{470} \sim$ 1800 $M^{-1}cm^{-1}$ and $\varepsilon_{420\text{-}430} \sim$ 2600-2800 $M^{-1}cm^{-1}$, respectively.

Accordingly, the following speciation could be drawn from the spectra collected: the complexes formation starts at pH<1 involving two hydroxamate groups coordinated to Fe(III) ion, while the three hydroxamate complexes are observed over pH range 3-9. Above pH 9, the absorbance begins to decrease, while the baseline starts to slightly increase, most probably because of the hydrolysis of ferric ions which competes with the complexation process. Of importance, to calculate the thermodynamic stability constants we used the experimental data only from the pH range where no decrease of absorbance was observed.

The formation of the dihydroxamate [FeH**L1**]$^+$ complex form were observed by spectroscopic evolutions of 470 nm band at the pH range 1.0 up to 2.35; then the maximum was shifted slightly towards shorter wavelengths (λ_{max}=430 nm) with pH increase up to 6.1 and the formation of trihydroxamate [Fe**L1**] species (Fig. S4). The thermodynamic stability constants of the $log\beta_{[FeHL1]+}$ = 31.8(3) (ε_{470} = 2030 $M^{-1}cm^{-1}$) and $log\beta_{[FeL1]}$ = 28.59(2) (ε_{430} = 2700 $M^{-1}cm^{-1}$), pK= 3.21, were calculated and listed in Table 2. The pK calculated for the same deprotonation step by refinement of the potentiometric data is 2.99 (the $log\beta_{[FeHL2]+}$ was kept fixed during the potentiometric data evaluation), which is again in good agreement.

a) b)

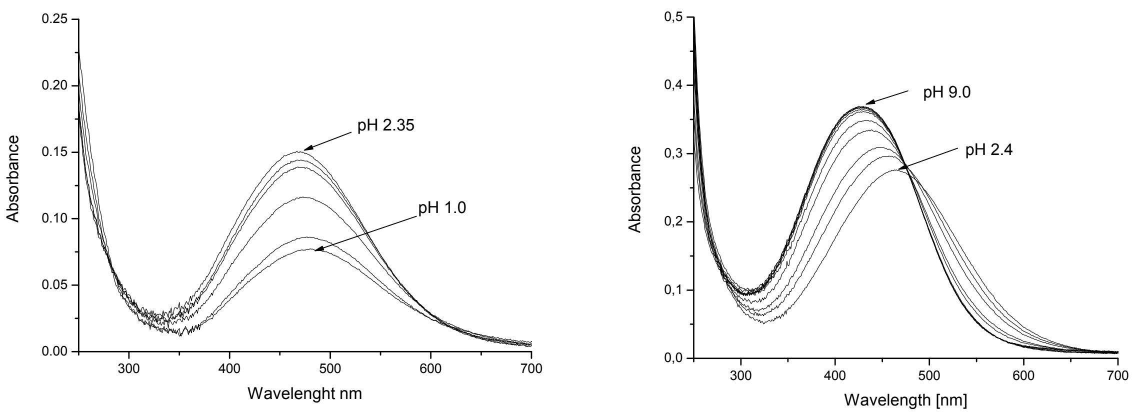

Fig. S4. The UV-Vis spectra of Fe(III)-$\mathbf{H_3L1}$ system at the metal-to-ligand molar ration 1:1 at pH range 1.0-2.35, $c_{Fe(III)}$=0.082 mM (a), and 2.4 – 9.0, $c_{Fe(III)}$=0.15 mM (b); I = 0.1 M $NaClO_4$, T = 25°C.

The formation of the dihydroxamate [FeH$_2$**L2**]$^+$ complex species was observed by spectroscopic evolutions of the wide band with λ_{max} at 470 nm, when the pH raised form 0.1 up to 1.9 (Fig. S5). The $log\beta_{[FeH2L2]+}$ calculated is 39.96(3) with the extinction coefficient ε_{470} = 2150 $M^{-1}cm^{-1}$.

a) b)

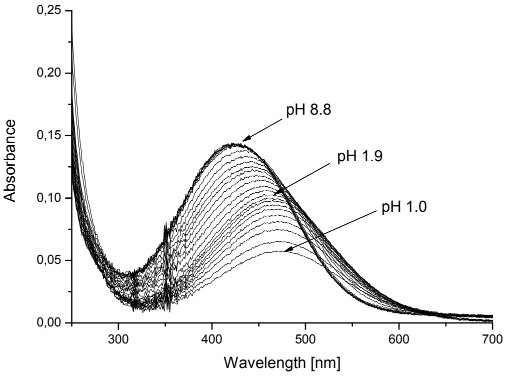

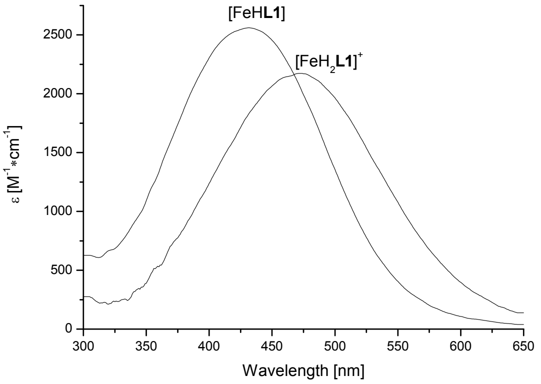

Fig. S5. The UV-Vis spectra of Fe(III)-$\mathbf{H_4L2}$ system at the metal-to-ligand molar ratio 1:1 at pH range 1.0-8.8, $c_{Fe(III)}$=0.048 mM (a); electronic spectra of ferric complexes with $\mathbf{H_4L2}$ (b); I = 0.1 M $NaClO_4$, T = 25°C.

Starting from a pH 2 up to 4.5 the new band appears at 430 nm and isosbestic point at 470 nm is observed, suggesting the formation of fully coordinated trihydroxamate [FeH**L2**] complex with the *logβ* = 36.96(7) (ε_{430} = 2570 $M^{-1}cm^{-1}$). The pK value calculated from UV-Vis data is 3.00(6), while the pK calculated for the same deprotonation step from the potentiometric data refinement is 3.14(6) ($log\beta_{[FeHL2]+}$ 36.96(7) was kept fixed during potentiometric data refinement), what is in good agreement (Table 2).

Allowing the structural difference and change in ionic strength, the extinction coefficients of both $\mathbf{H_3L1}$ and $\mathbf{H_4L2}$ systems for the di- and trihydroxamate complexes are in excellent agreement with previously published data (Table S2).[1,3,6,7] Based on the UV-Vis data distribution diagrams were calculated and are shown in the Fig. 6S.

The thermodynamic study of Fe(III)-$\mathbf{H_3L3}$ and Fe(III)-$\mathbf{H_3L4}$ complexes were already published elsewhere,[1,8] and the data were used here when needed.

Table S2. Comparison of the extension coefficients ($M^{-1}cm^{-1}$) of the Fe(III) complexes.

Form of Fe(III) complexes	Ligands					
	$\mathbf{H_3L1}$[a]	$\mathbf{H_4L2}$[a]	DFOE[b3]	DFOB[3b]	DFOB[6c]	$\mathbf{H_3L4}$(T4)[1d]
Dihydroxamate complex form	ε_{470} 2030	ε_{470} 2170	-	-	ε_{477} 2258	ε_{472} 2130
Trihydroxamate complex form	ε_{430} 2700	ε_{430} 2570	ε_{435} 2620	ε_{440} 2640	ε_{435} 2454	ε_{430} 2750

[a]results obtained in this work, I = 0.1M $NaClO_4$, T = 25°C; [b]0.1 M $NaNO_3$, 20°C; [c]1M $NaClO_4$, 25°C; [d]0.1M/1M $NaClO_4$, 25°C.

a) b)

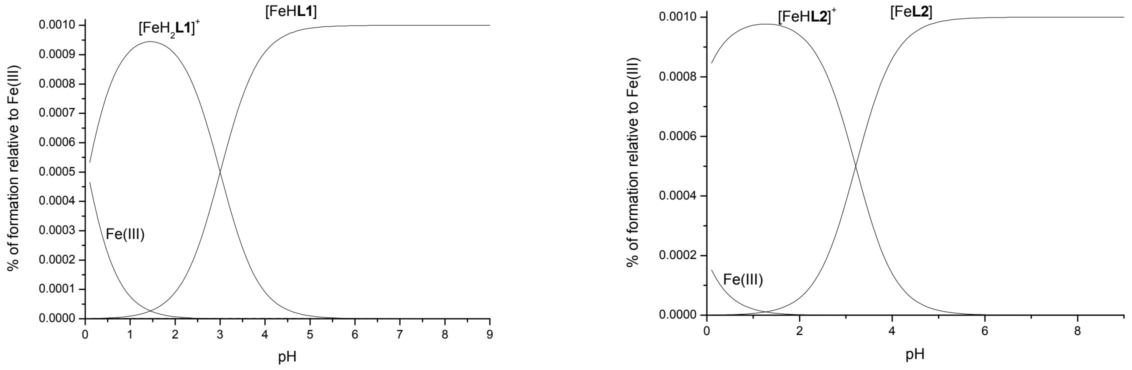

Fig. S6. Calculated species distribution diagram for Fe(III)- $\mathbf{H_3L1}$ (a) and Fe(III)- $\mathbf{H_4L2}$ (b) systems; $c_{Fe(III)}$ =1 mM, c_L=1 mM; log β values for $\mathbf{H_3L1}$ and $\mathbf{H_4L2}$ are given in Table 1; log β values for complexes $[FeH_2\mathbf{L1}]^+$, [FeH**L1**], $[FeH\mathbf{L2}]^+$ and [Fe**L2**] are given in Table 2; log β values for hydroxocomplexes of Fe(III) are given in 'Experimental section'.

Ga(III) complex formation equilibria

a) b)

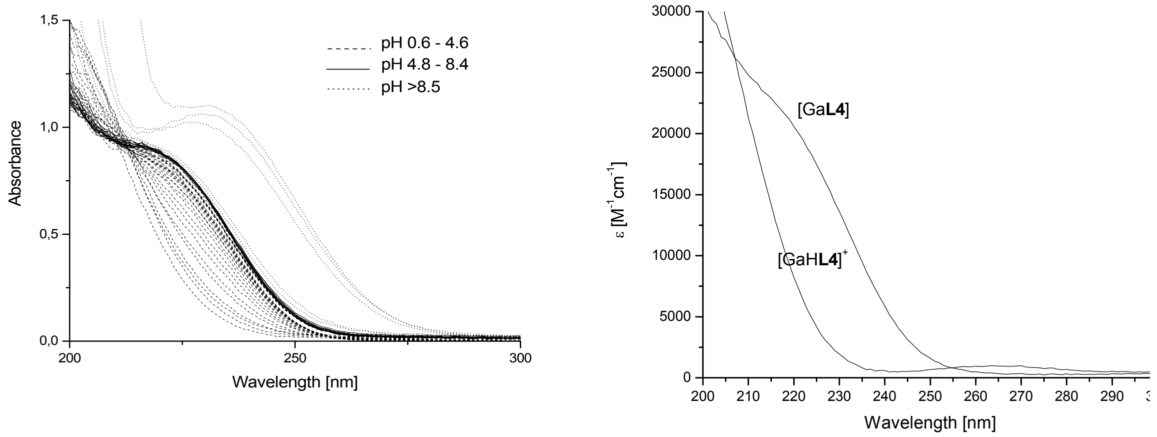

Fig. S7. The UV-Vis spectra of Ga(III)-$\mathbf{H_3L4}$ system at the metal-to-ligand molar ratio 1:1 in pH range 0.6-11.0, c_{L4} = 0.05 mM, 0.1 M $NaClO_4$, T=25°C.

a) b)

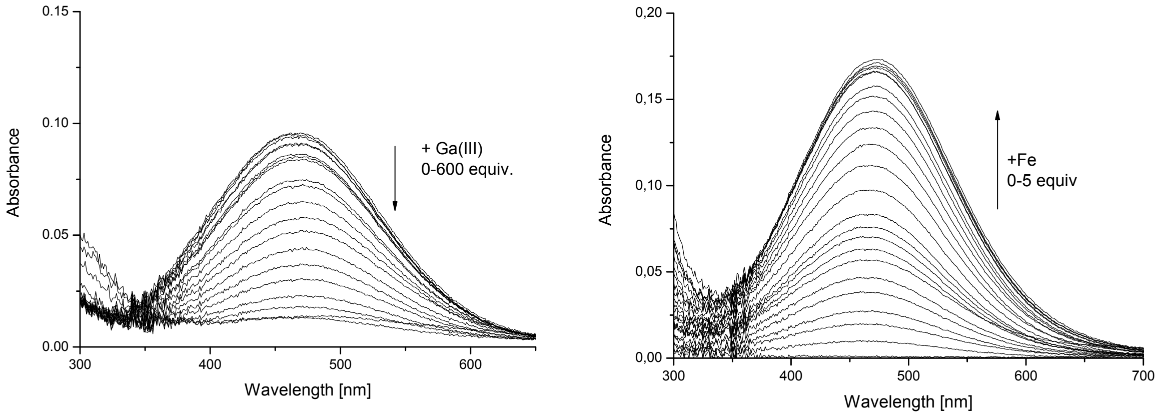

Fig. S8. The UV-Vis metal-metal competition titrations for Fe(III)-$\mathbf{H_4L2}$+Ga(III) ($c_{Fe(III)}$ = 0.05 mM, $c_{\mathbf{L2}}$ = 0.05 mM) (a) and Ga(III)-$\mathbf{H_4L2}$+Fe(III) ($c_{Fe(III)}$ = 0.082 mM , $c_{\mathbf{L2}}$ = 0.082 mM) (b) systems at 1.5 pH, I = 0.1 M $NaClO_4$, T = 25˚C.

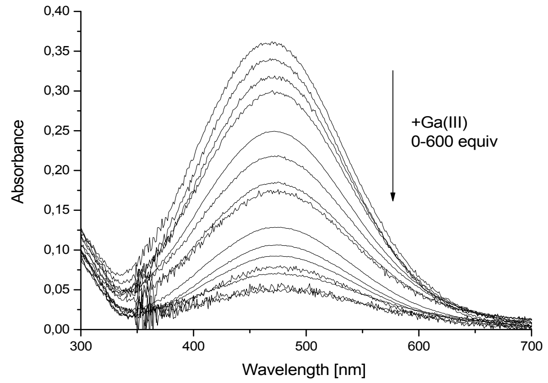

Fig S9. The UV-Vis metal-metal competition experiment for Ga(III)-$\mathbf{H_3L4}$+Fe(III) ($c_{Fe(III)}$ = 0.200 mM , $c_{\mathbf{L4}}$ = 0.200 mM) system at 1.5 pH, 0.1 M $NaClO_4$.

Zr(IV) complex formation equilibria

Metal-metal competition experiment with NTA as an additional competitor agent in the case of Zr(IV)-DFOB system

The metal competition titrations are demonstrated in Fig. S10, and reflect the changes in UV–Vis absorbance upon the addition of 0 up to 50 equiv. of Zr(IV)-NTA solution to a solution of Fe(III)-DFOB; the large LMCT band centered at 430 nm characteristic of trihydroxamate $[FeHDFOB]^+$ complex form decreased gradually.[9] The refinement of the titration data, using the Fe(III)-DFOB,[6] Fe(III)-NTA,[10] Zr(IV)-NTA[11] stability constants, together with the Fe(III)[12] and Zr(IV)[13] hydrolysis constants, yielded a $\log\beta_{ZrHL}$ value of 45.9 (2) (spectra collected after 1h incubation) and 46.1 (2) (spectra collected after 24h incubation). Allowing for the change in ionic strength, the data obtained in the present work are in a very good agreement with those published earlier by us.[6]

a) b)

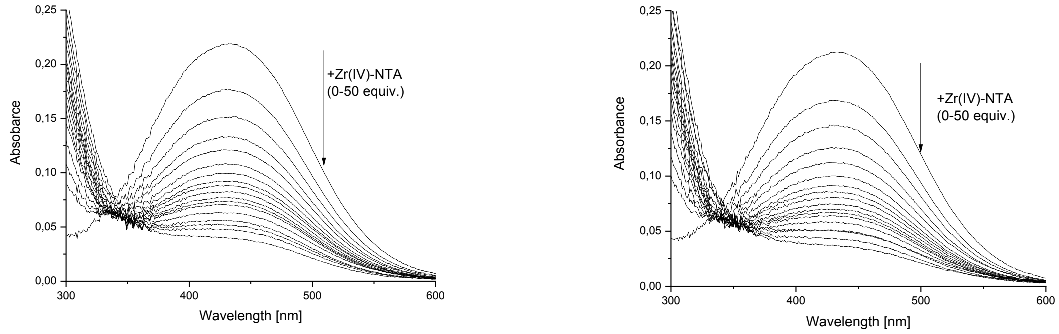

Fig. S10. The UV-Vis spectra of competition titration of Fe(III)-DFOB+Zr(IV)-NTA system at pH 2, collected 1 (a) and 24 (b) hours after preparation: $c_{Fe(III)} = 0.08$ mM, $c_{DFOB} = 0.08$ mM; Zr(IV)-NTA solution was added at the metal-to-ligand molar ration 1:1, from 0 up to 50 equiv. of Zr(IV); $I = 0.1$ M $NaClO_4$, T = 25°C.

Zr(IV) complex formation equilibria for H_3L3, H_3L4 and DFOE

a)

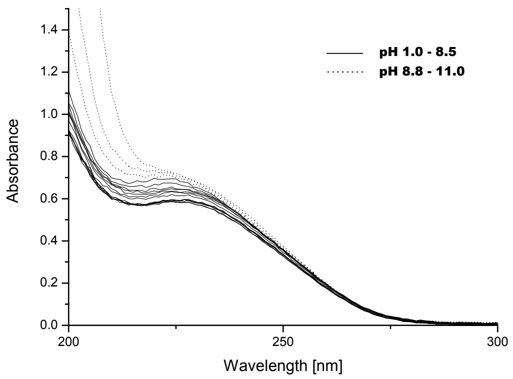

b)

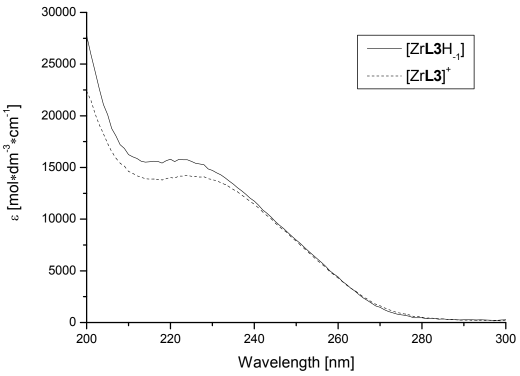

c)

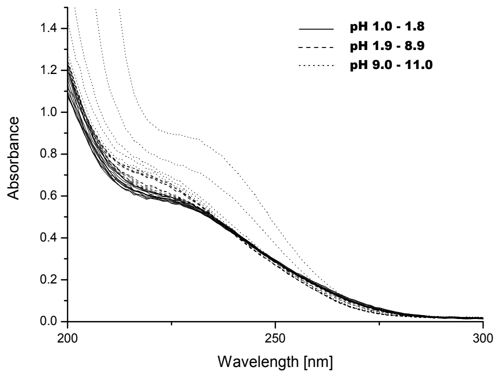

d)

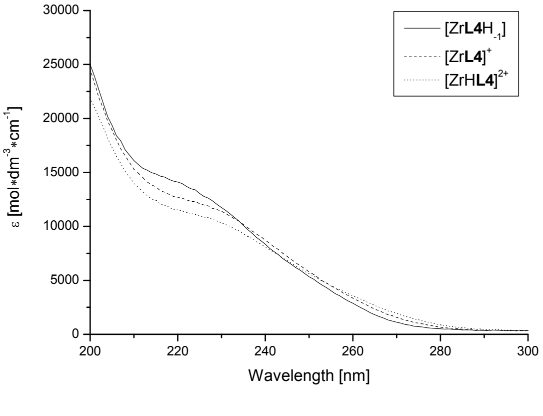

Fig S11. The pH-depended UV-Vis titration of the Zr(IV)- $\mathbf{H_3L3}$, $c_{Zr(IV)} = 0.045$, $c_{\mathbf{L3}} = 0.05$ (a); Zr(IV)-$\mathbf{H_3L4}$, $c_{Zr(IV)} = 0.045$, $c_{\mathbf{L4}} = 0.050$ (c) systems; together with the calculated electronic spectra Zr(IV)-$\mathbf{H_3L3}$ (b), Zr(IV)-$\mathbf{H_3L4}$ (d). All measurements were performed at 0.1 M $NaClO_4$, 25°C, exact pH ranges are written at each spectra.

a)

b)

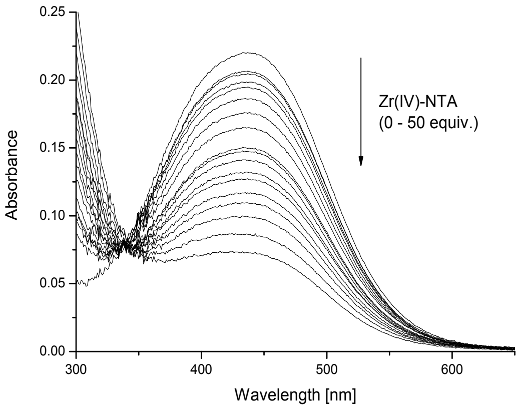

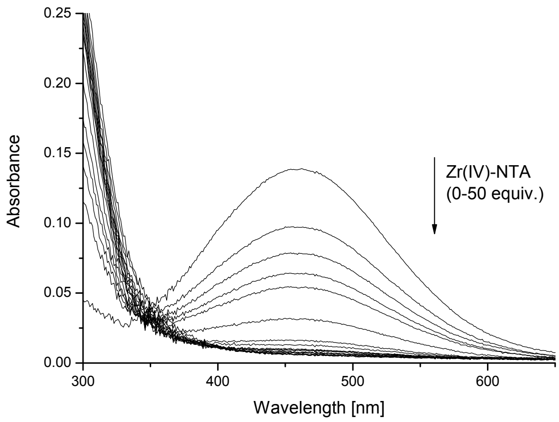

d)

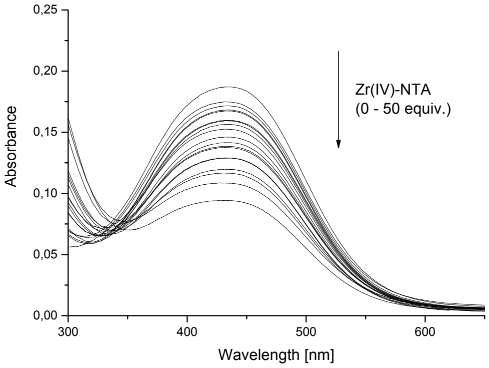

Fig. S12. The metal-metal competition titration of Fe(III)-$\mathbf{H_3L3}$ $c_{Fe(III)} = c_{\mathbf{L3}} = 0.08$ mM (a); Fe(III)-$\mathbf{H_4L4}$ $c_{Fe(III)} = c_{\mathbf{L4}} = 0.06$ mM (b), Fe(III)-**DFOE**, $c_{Fe(III)} = c_{\mathbf{DFOE}} = 0.07$ mM (c), all measurements were performed at 0.1 M $NaClO_4$, 25°C, pH = 2.0.

a)

b)

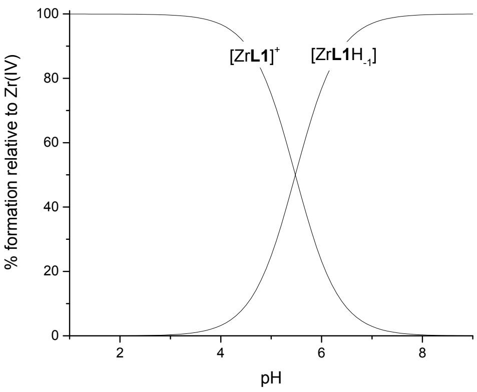

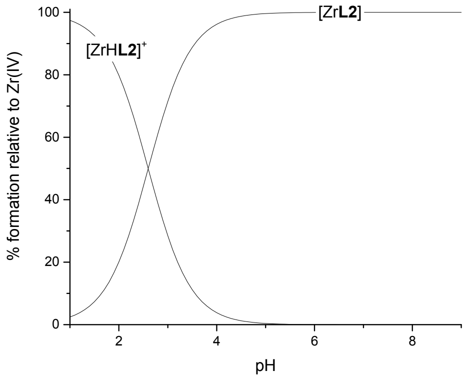

c)

d)

14

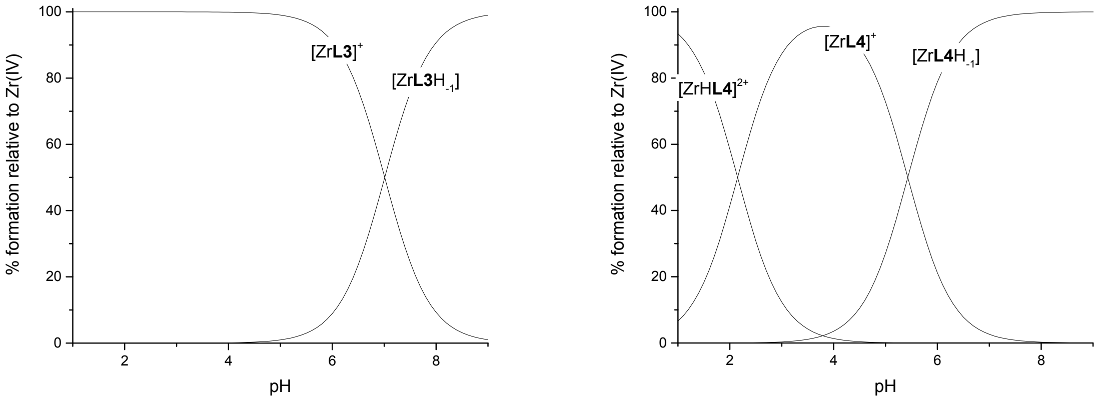

Fig. S13. Calculated species distribution diagrams for Zr(IV) - $\mathbf{H_3L1}$ (a), Zr(IV)- $\mathbf{H_4L2}$ (b), Zr(IV) - $\mathbf{H_3L3}$ (c) and Zr(IV) - $\mathbf{H_3L4}$ (d) systems; $c_{Zr(IV)}$= 1 mM, c_L= 1 mM; logβ values for ligands and complexes are given in Table 1; logβ values for hydroxocomplexes of Zr(IV) are given in Experimental section.

Ligands' sequestering ability

Table S3. *p*Fe values with various synthetic and biological chelators[a]

Ligand	*p*Fe	Chelating groups and ligands geometry
$\mathbf{H_3L1}$	24.3	3 hydroxamate groups in a cyclic arrangement
$\mathbf{H_4L2}$	23.3	4 hydroxamate groups in a linear arrangement
$\mathbf{H_3L3}$	26.9	3 hydroxamate groups in a cyclic arrangement
$\mathbf{H_3L4}$	23.6[1]	3 hydroxamate groups in a linear arrangement
DFOB	26.1[6]	3 hydroxamate groups in a linear arrangement
DFOE	27.7[3]	3 hydroxamate groups in a cyclic arrangement

[a]the values (re)calculated at pH=7.4 with c_L=10 µM and $c_{Ga(III)/Zr(IV)}$=1 µM, and based on the protonation and stability constants given in original publications; the hydrolysis constants of Fe(III) ions were taken from the literature[14] and are given in the experimental section

Experimental section

^{1}H NMR and ^{13}C NMR spectra obtained for intermediate products. HR-ESI-MS for H_3L1 and H_4L2.

Compound 2

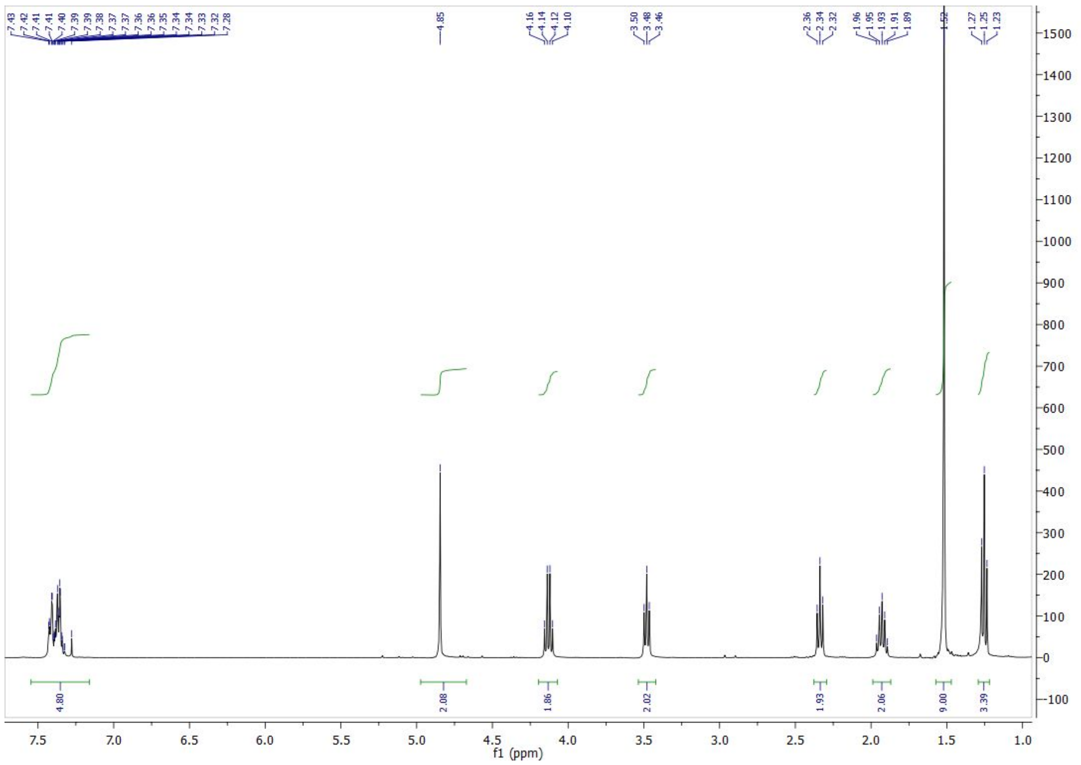

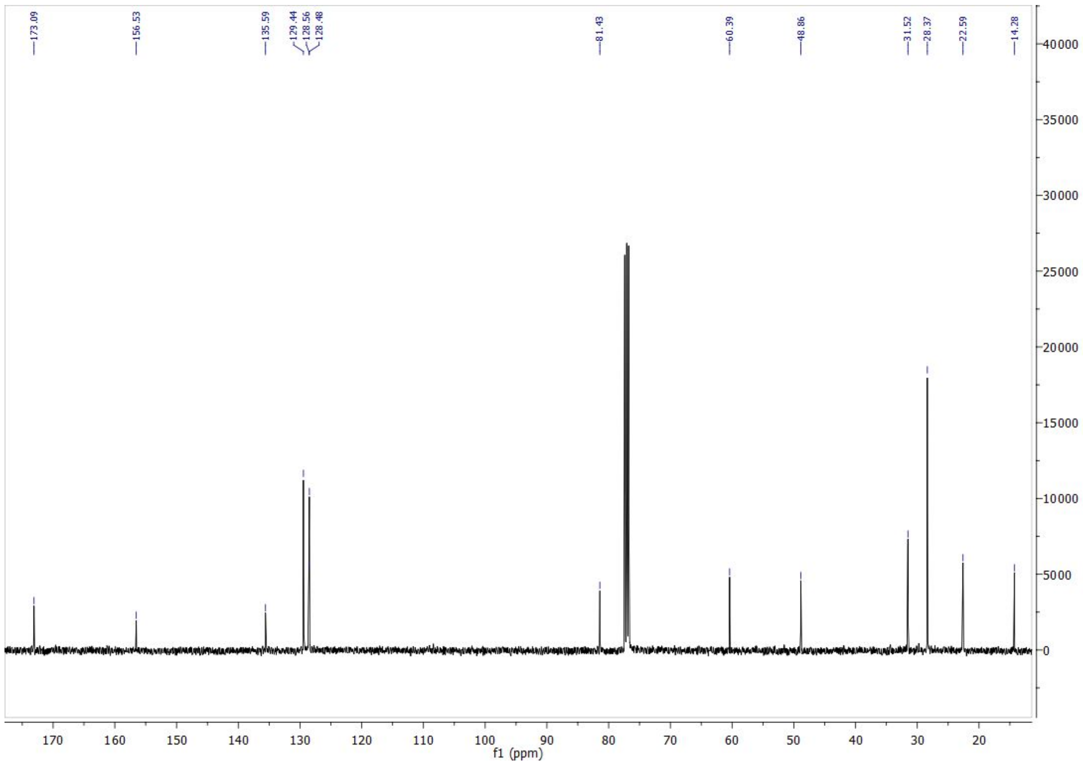
173.09
156.53
135.59
129.44
128.56
128.48
81.43
60.39
48.86
31.52
28.37
22.59
14.28
f1 (ppm)

Compound 4

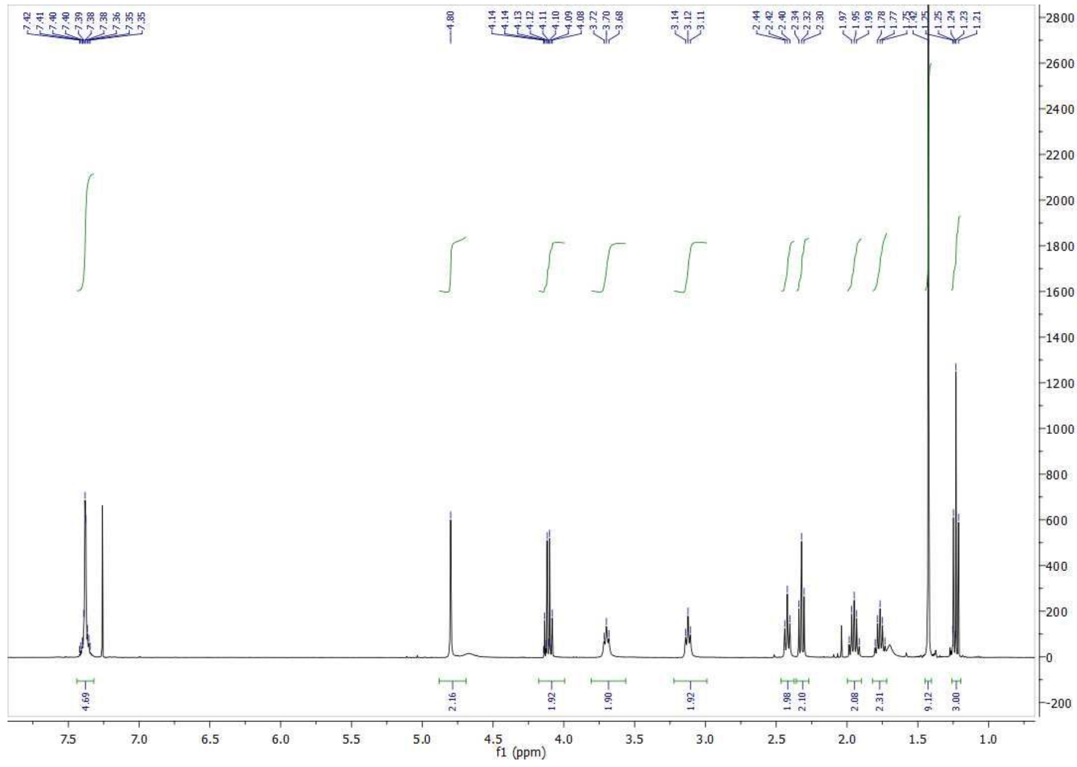
4.80
3.72
3.70
3.68
3.14
3.12
3.11
4.69
2.16
1.92
1.90
1.92
1.98
2.10
2.08
2.31
9.12
3.00
f1 (ppm)

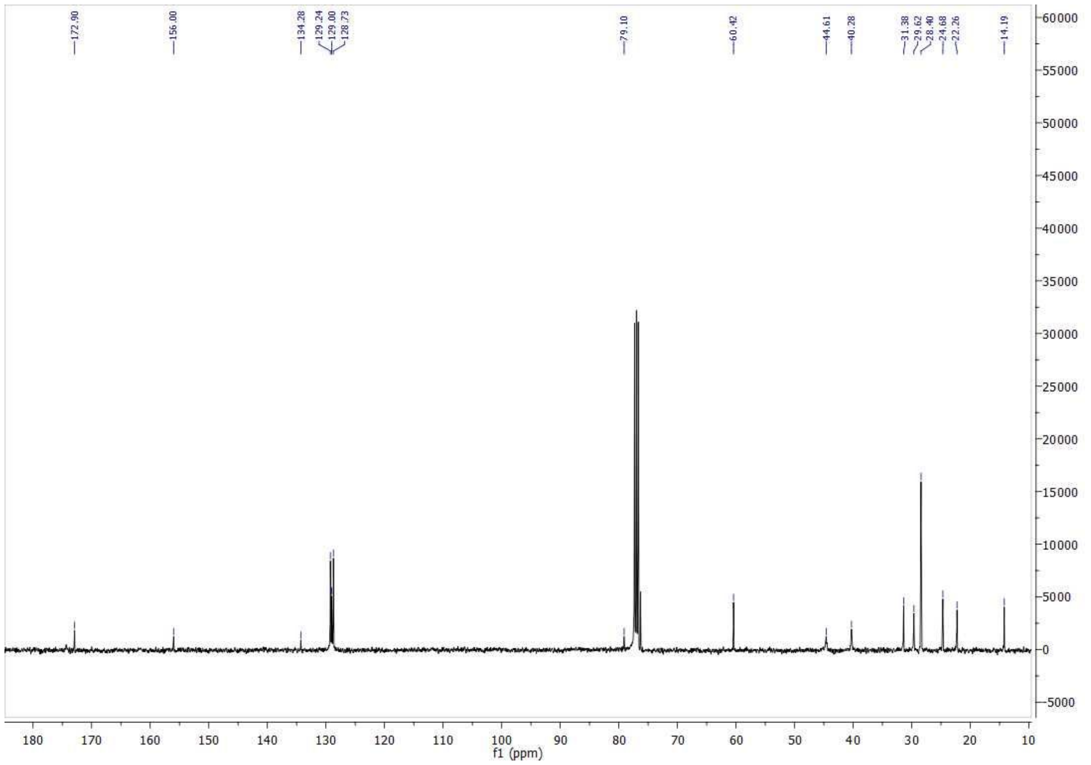
172.90
156.00
134.28
129.24
129.00
128.73
79.10
60.42
44.61
40.28
31.38
29.62
28.40
24.68
22.26
14.19
f1 (ppm)

Compound 7

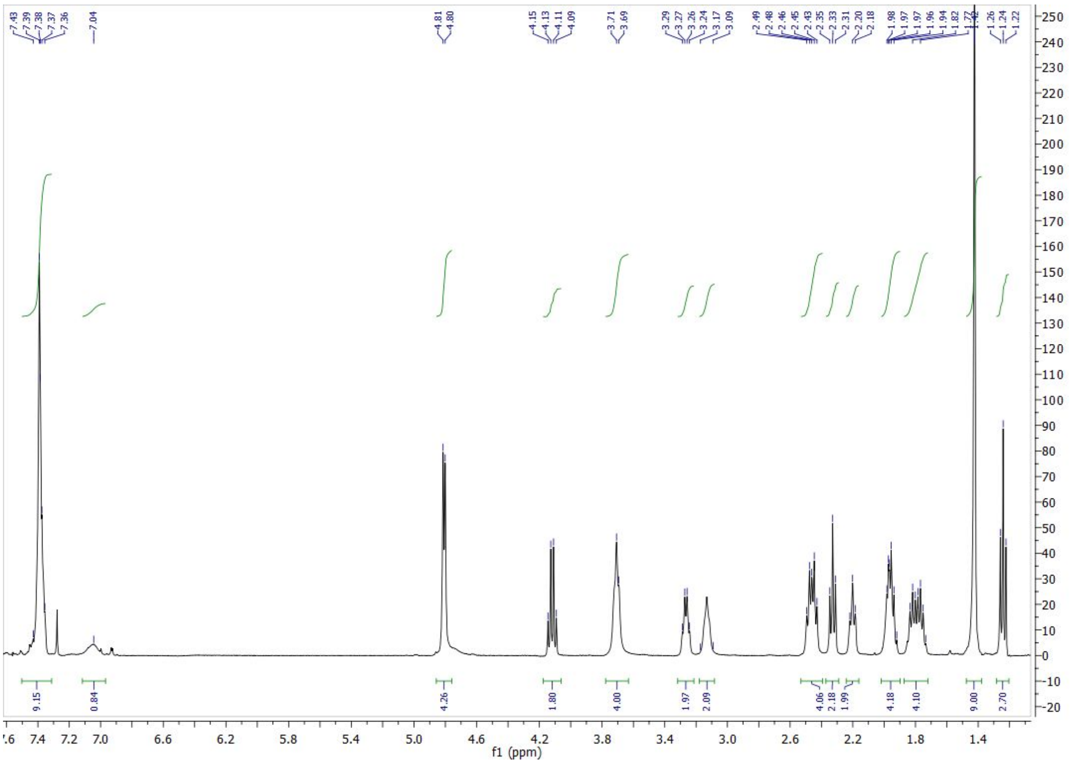

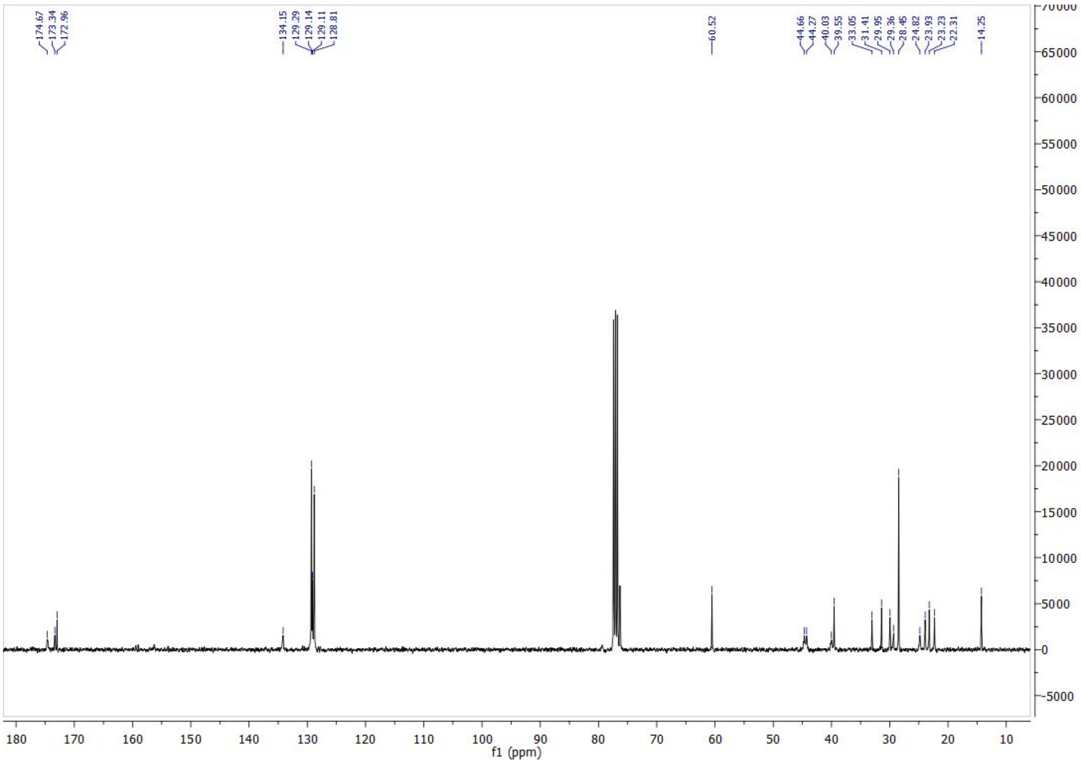

Compound 9

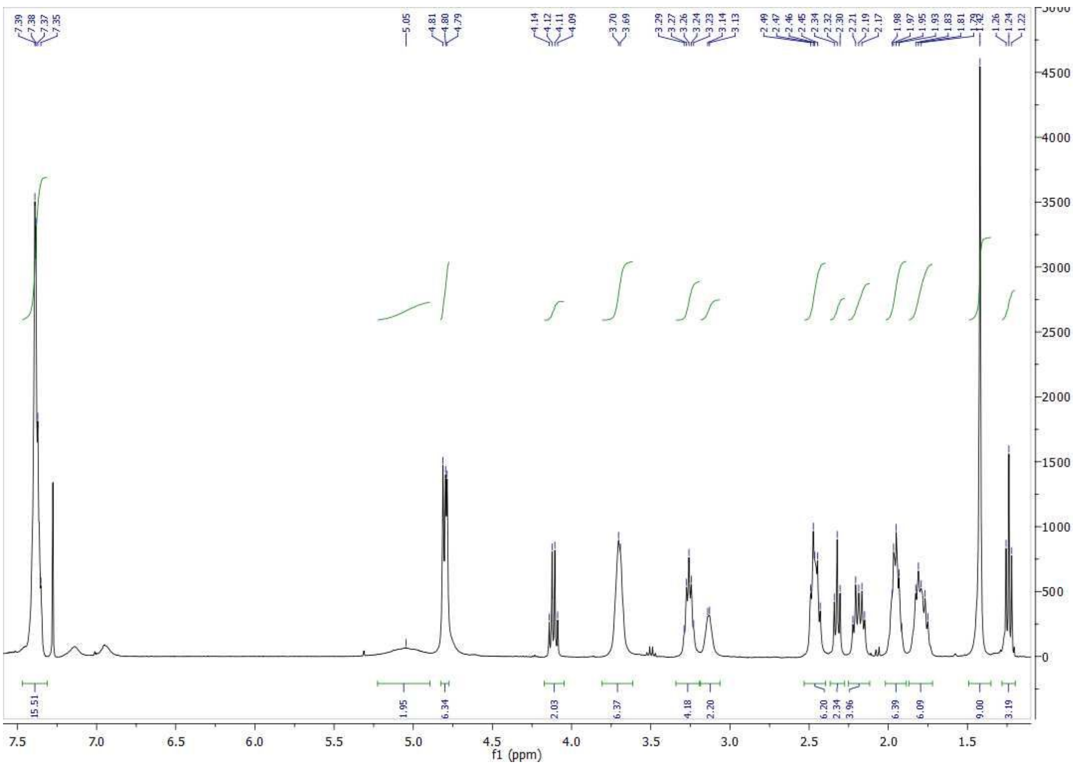

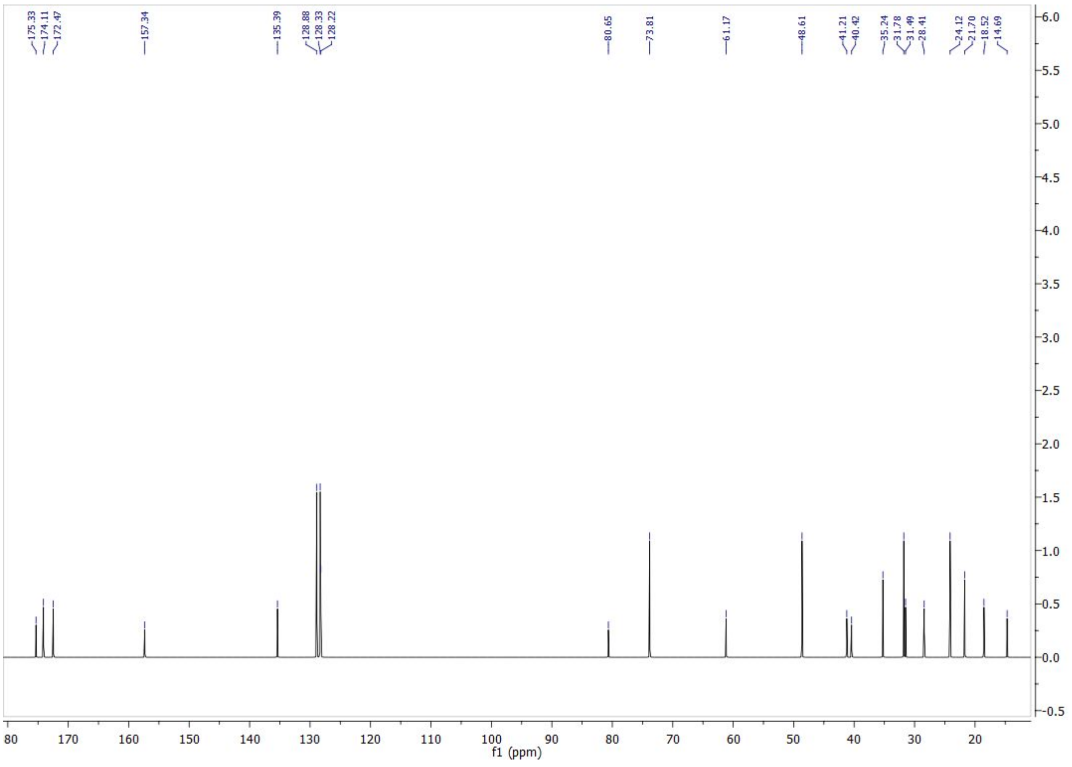

Compound 10

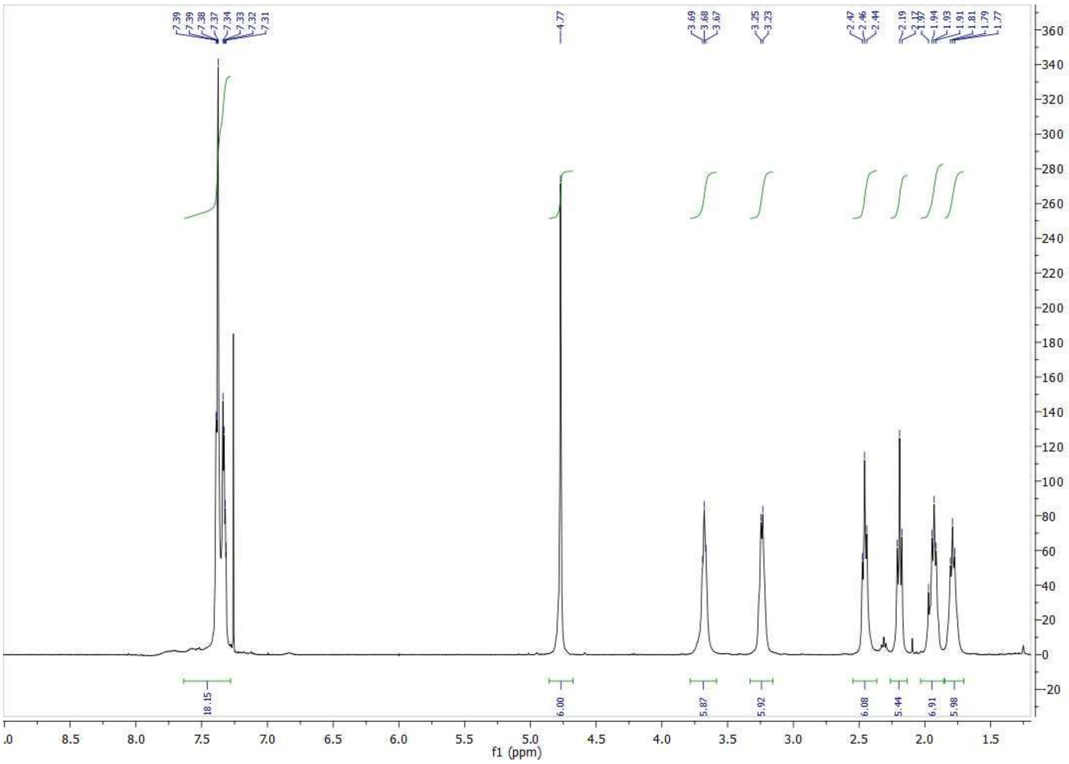
7.39
7.39
7.38
7.37
7.34
7.33
7.32
7.31
4.77
3.69
3.68
3.67
3.25
3.23
2.47
2.46
2.44
2.19
2.17
1.97
1.94
1.93
1.91
1.81
1.79
1.77
18.15
6.00
5.87
5.92
6.08
5.44
6.91
5.98
f1 (ppm)

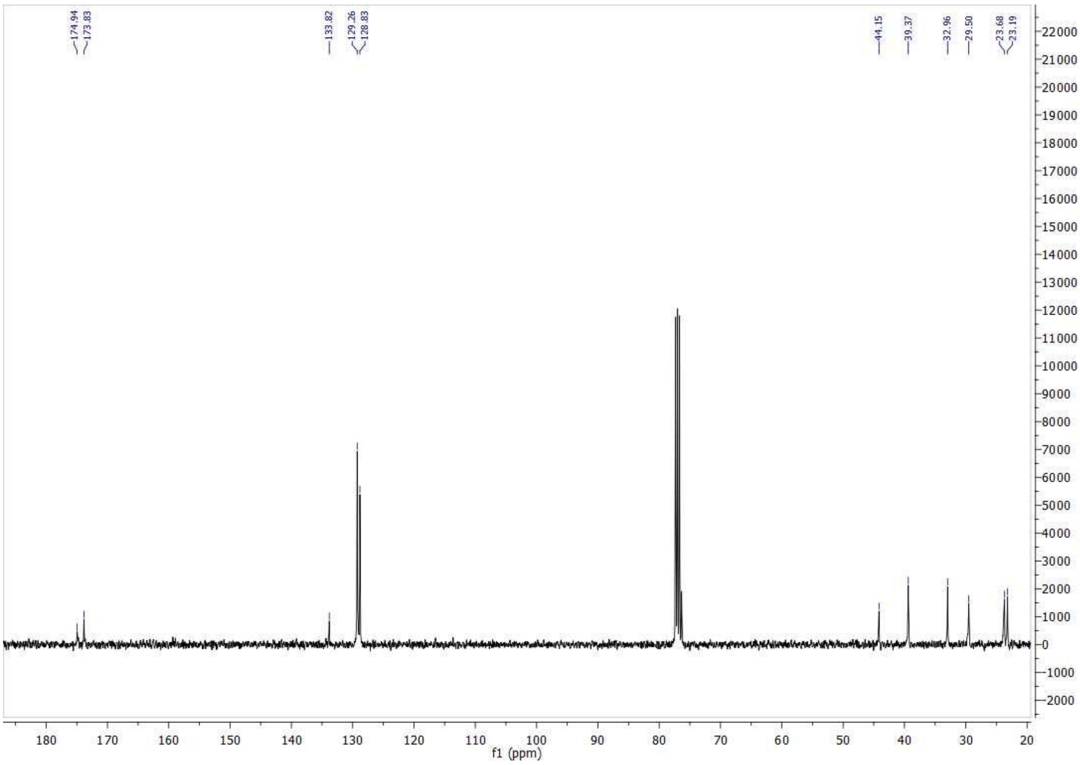
174.94
173.83
133.82
129.26
128.83
44.15
39.37
32.96
29.50
23.68
23.19
f1 (ppm)

Compound H_3L1 (or 11)

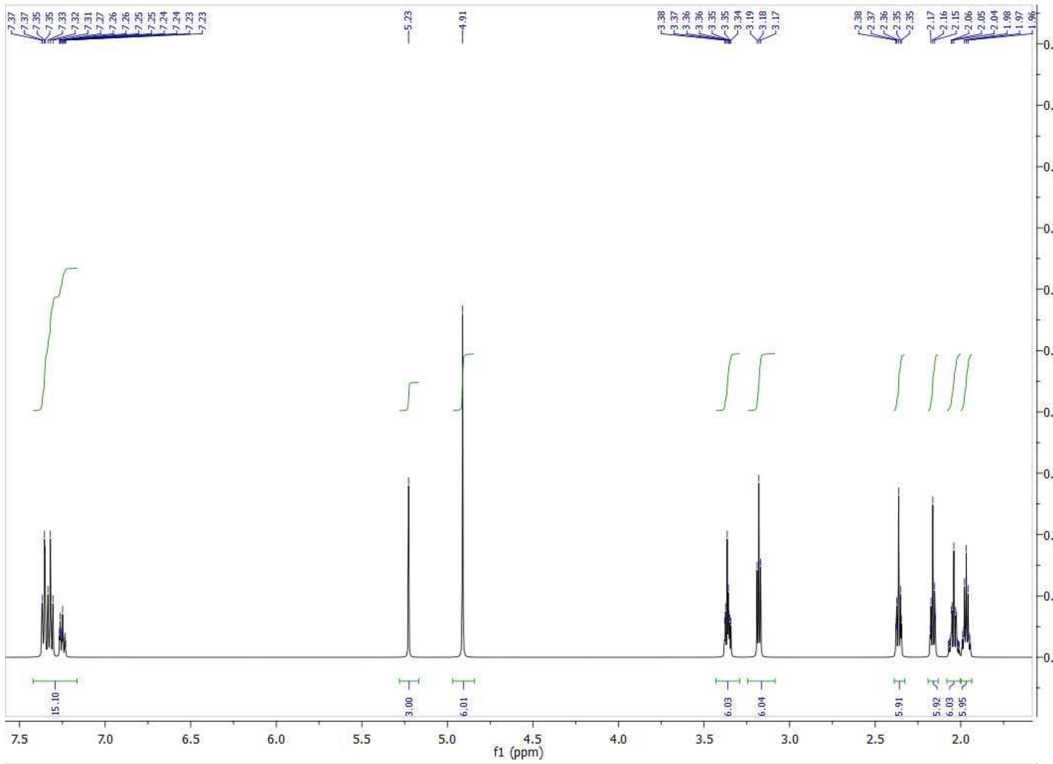

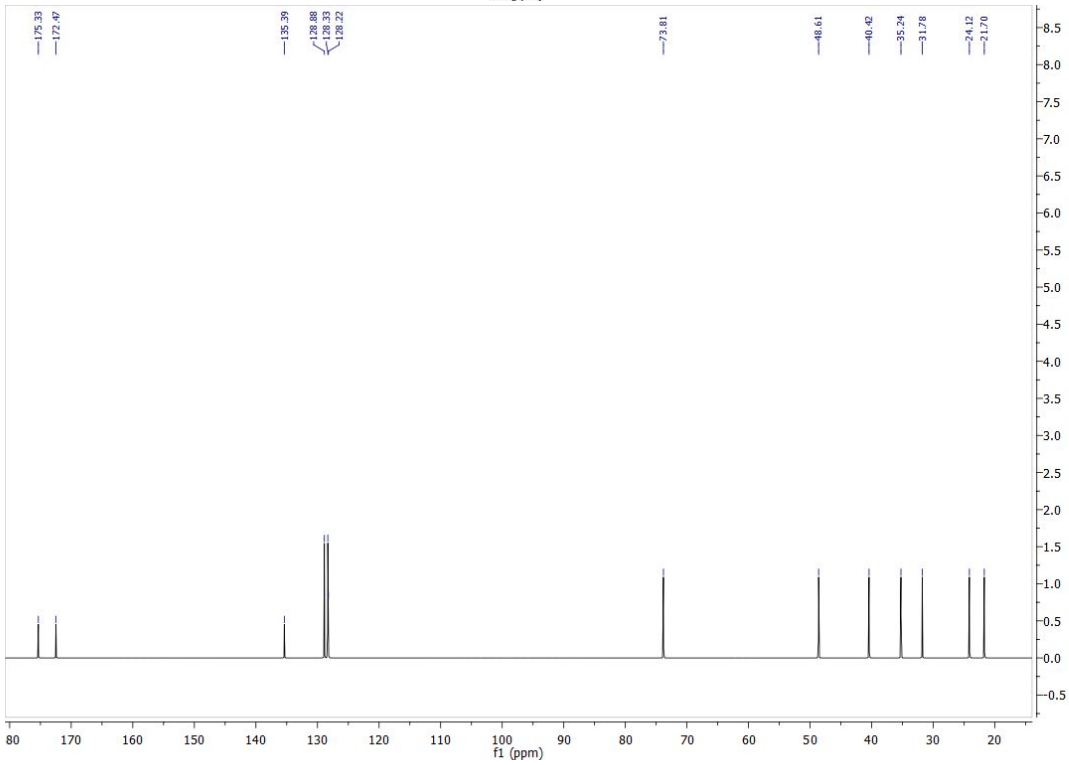

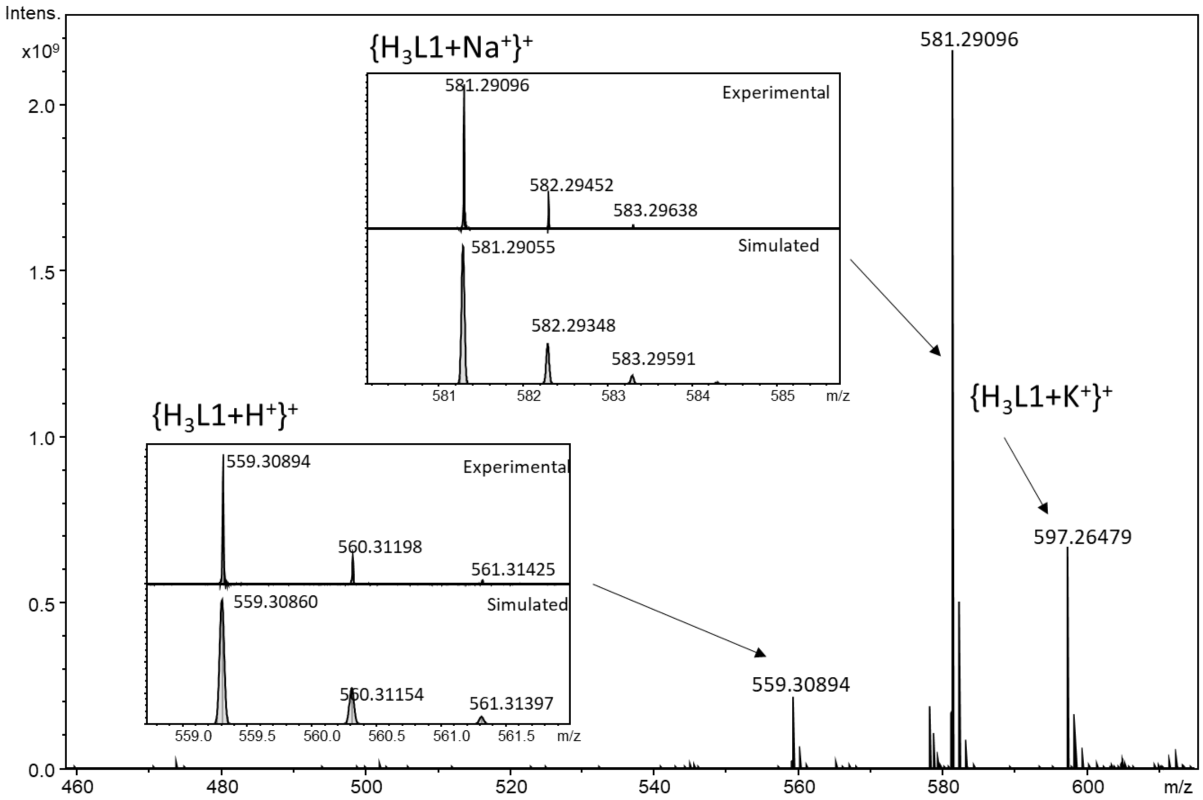
Intens.
x10^9
{H3L1+Na+}+
581.29096
Experimental
582.29452
583.29638
581.29055
Simulated
582.29348
583.29591
{H3L1+H+}+
559.30894
Experimental
560.31198
561.31425
559.30860
Simulated
560.31154
561.31397
{H3L1+K+}+
597.26479
559.30894
581.29096
m/z

Compound 13

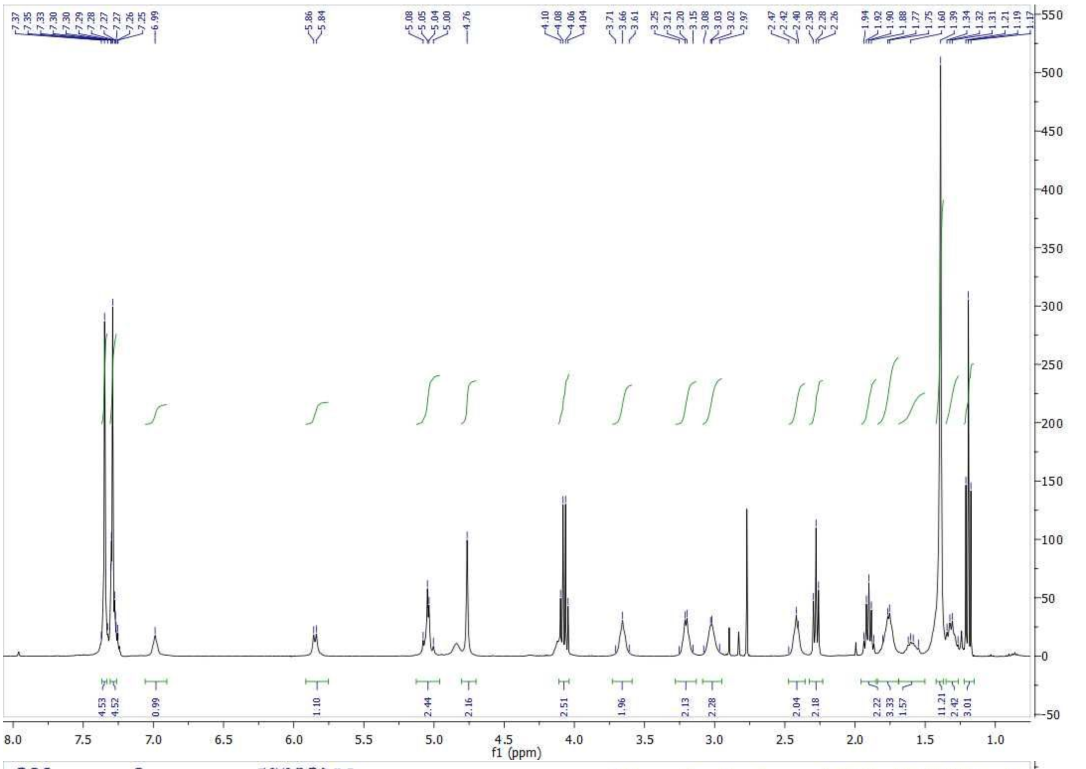

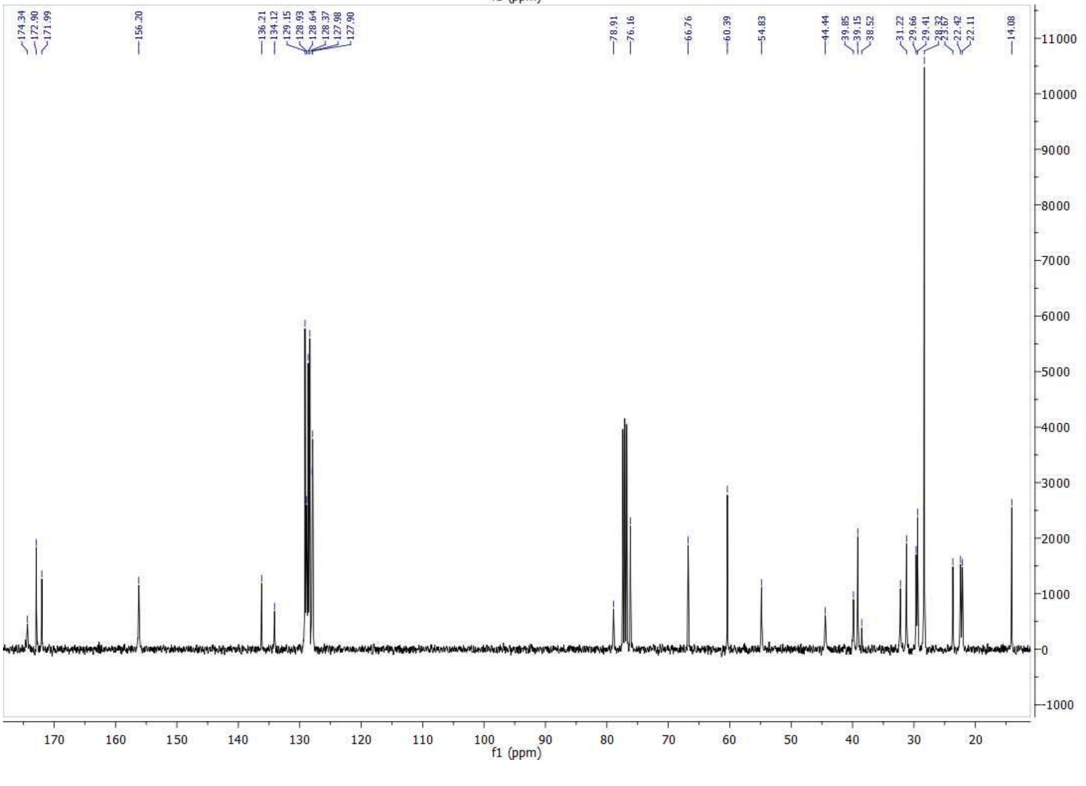

Compound 15

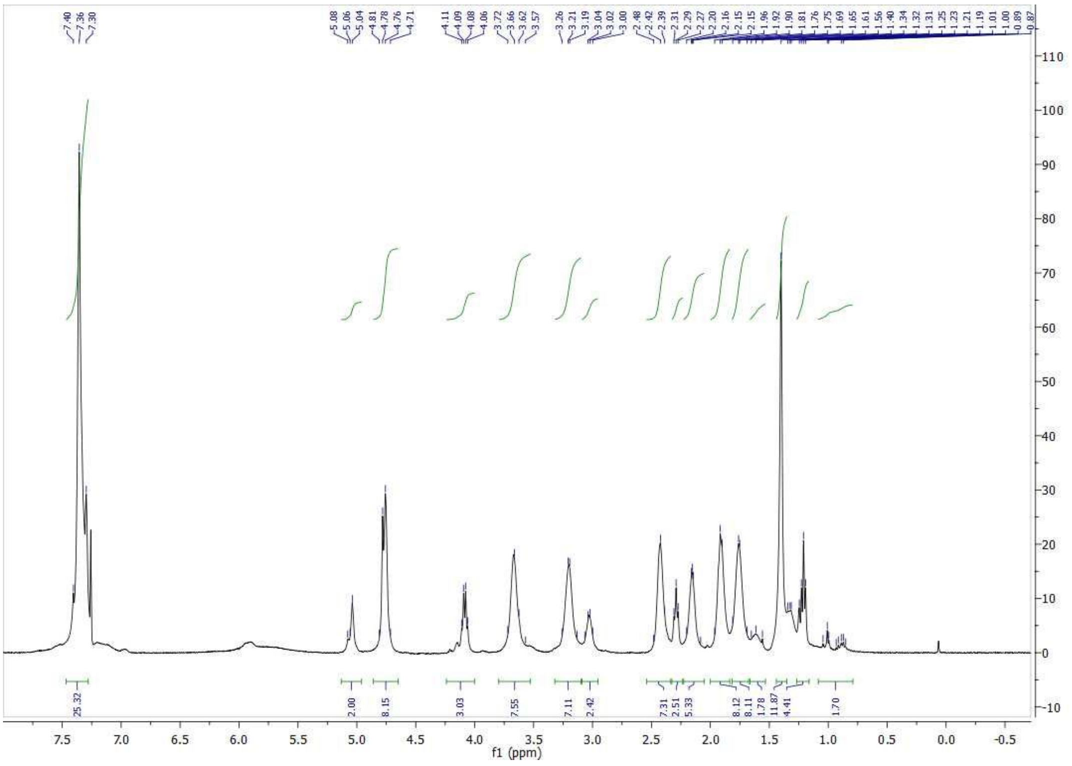

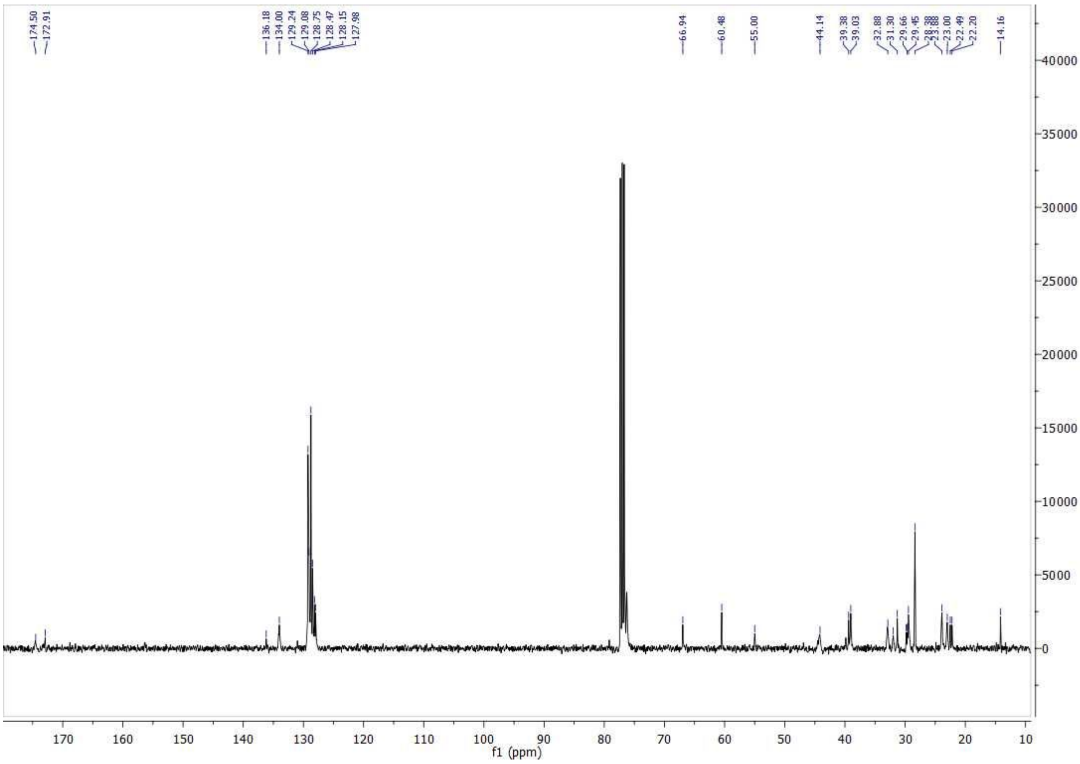

Compound 16

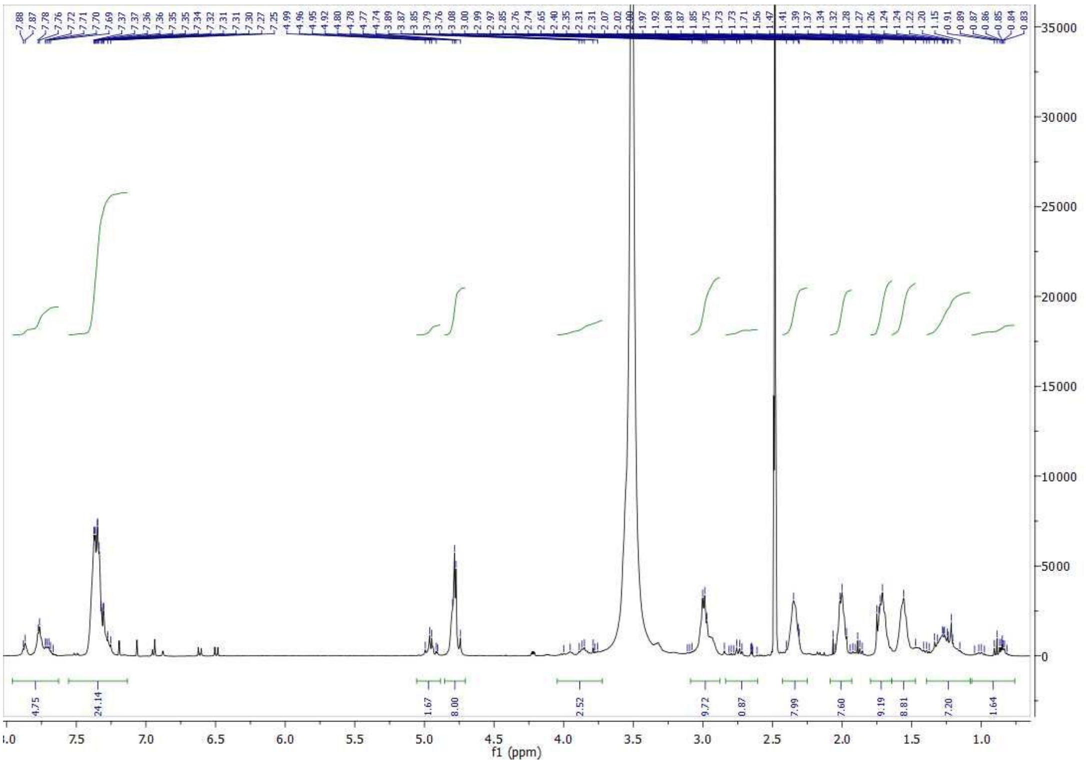

7.88 7.87 7.78 7.76 7.72 7.71 7.70 7.69 7.37 7.37 7.36 7.36 7.35 7.35 7.34 7.32 7.31 7.31 7.30 7.27 7.25 4.99 4.96 4.95 4.92 4.80 4.78 4.77 4.74 3.89 3.87 3.85 3.79 3.76 3.08 3.00 2.99 2.97 2.85 2.76 2.74 2.65 2.40 2.35 2.31 2.31 2.07 2.02 1.97 1.92 1.89 1.87 1.85 1.75 1.73 1.73 1.71 1.56 1.47 1.41 1.39 1.37 1.34 1.32 1.28 1.27 1.26 1.24 1.24 1.22 1.20 1.15 0.91 0.89 0.87 0.86 0.85 0.84 0.83
4.75 24.14 1.67 8.00 2.52 9.72 0.87 7.99 7.60 9.19 8.81 7.20 1.64
f1 (ppm)

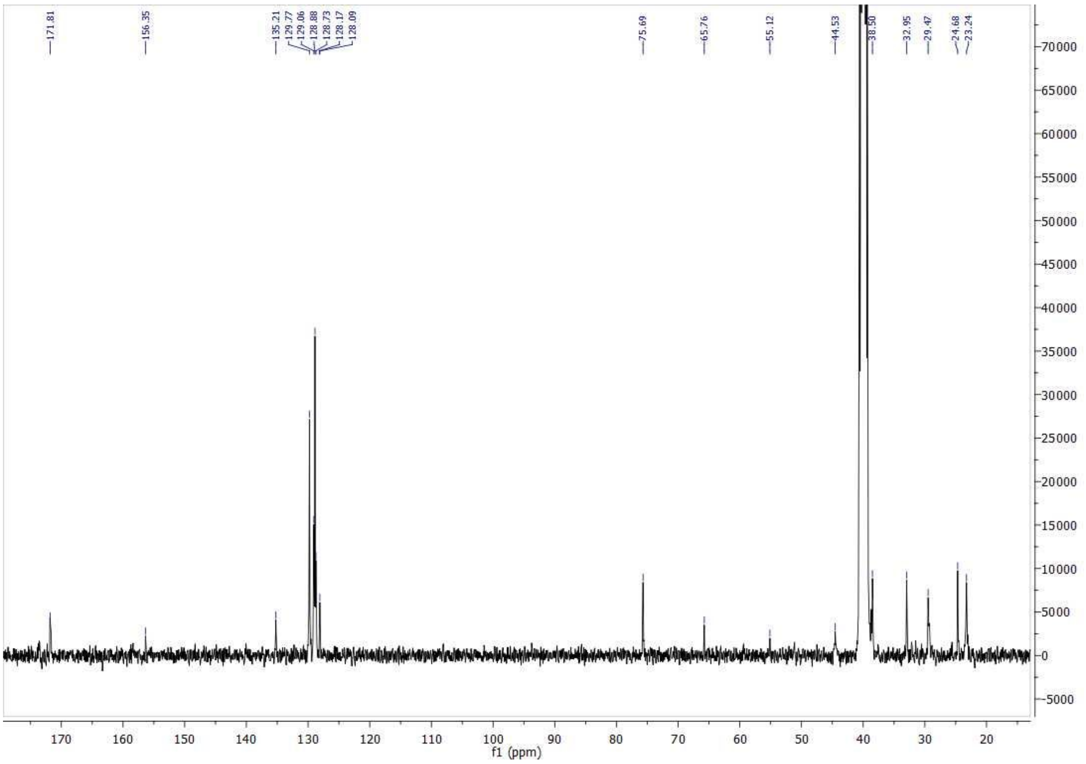

171.81 156.35 135.21 129.77 129.06 128.88 128.73 128.17 128.09 75.69 65.76 55.12 44.53 38.50 32.95 29.47 24.68 23.24
f1 (ppm)

Compound H_4L2 (or 17)

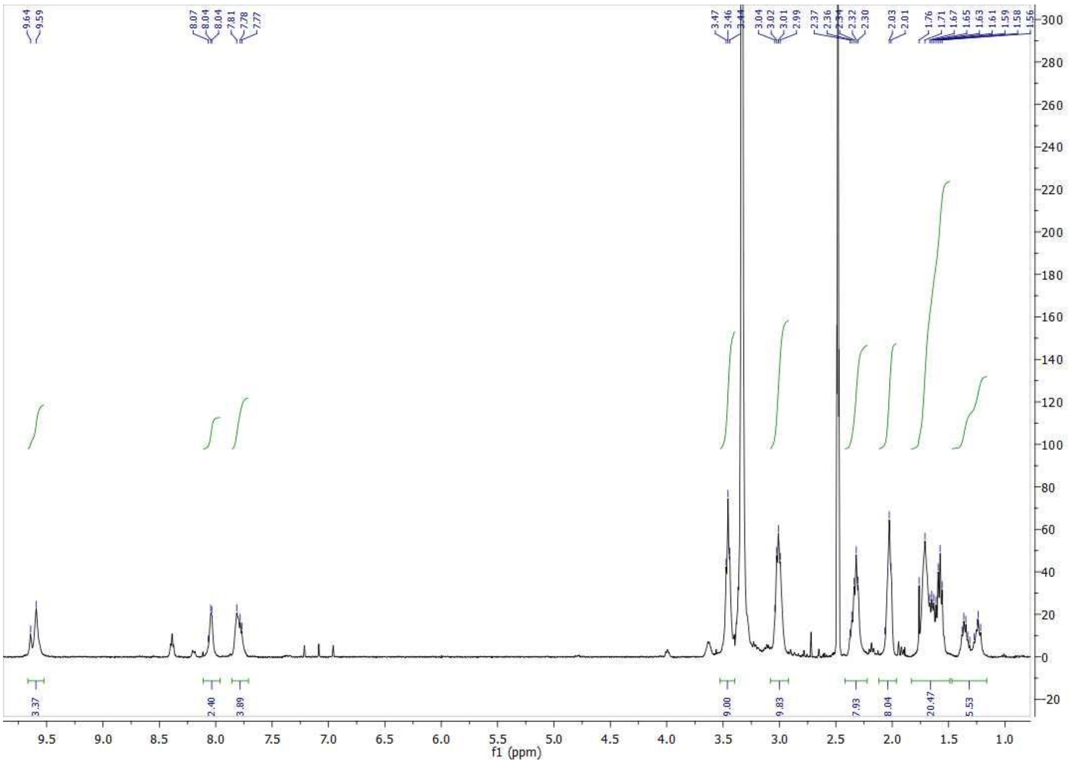

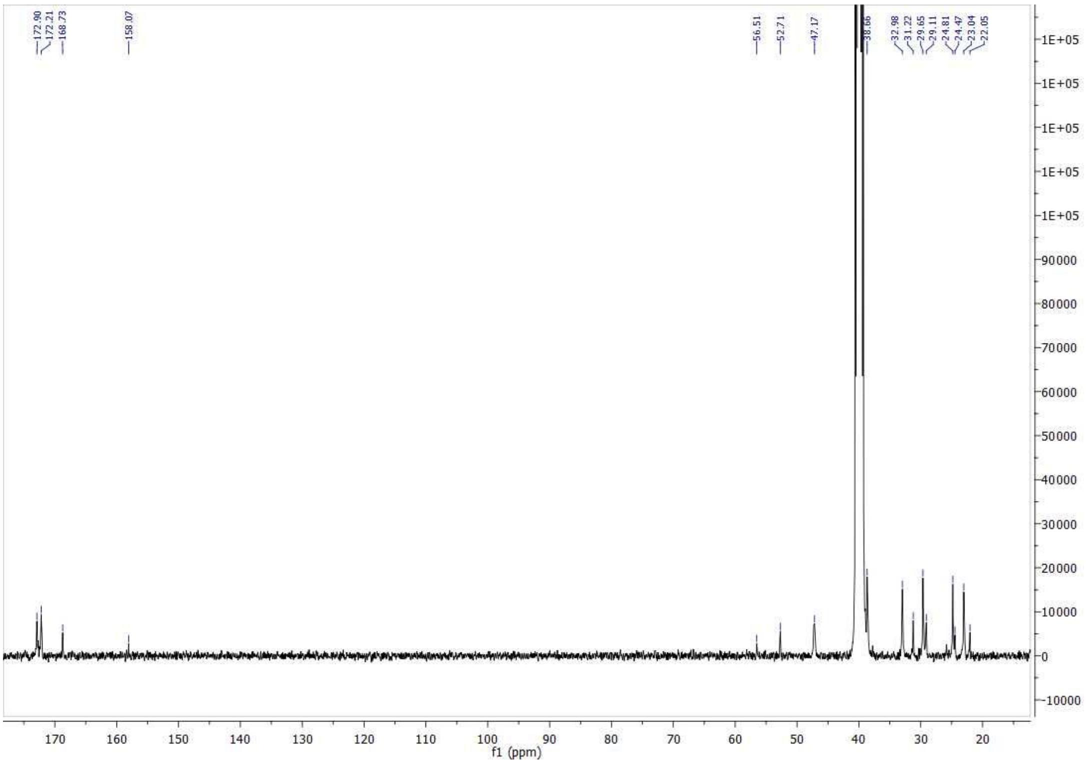

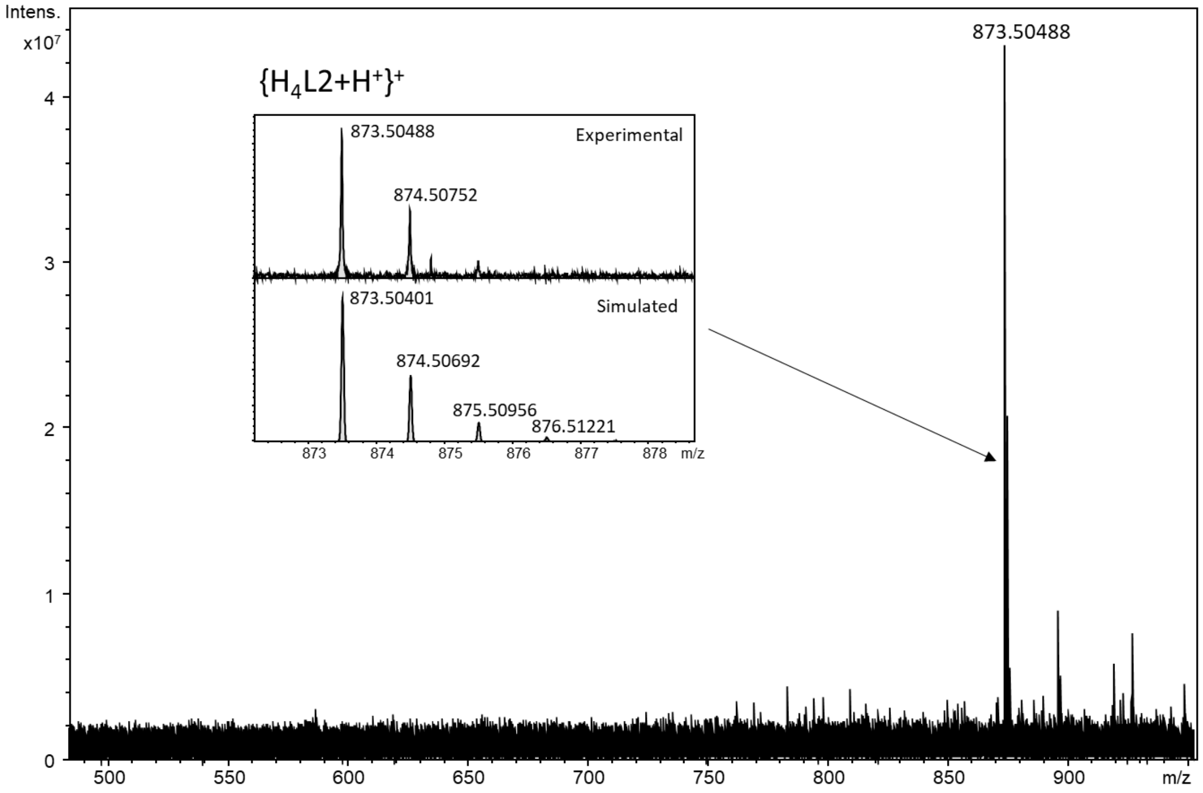
Intens.
x10^7
4
3
2
1
0
500
550
600
650
700
750
800
850
900
m/z
873.50488
{H4L2+H+}+
873.50488
Experimental
874.50752
873.50401
Simulated
874.50692
875.50956
876.51221
873
874
875
876
877
878
m/z

Analitycal HPLC

Compound H_3L1

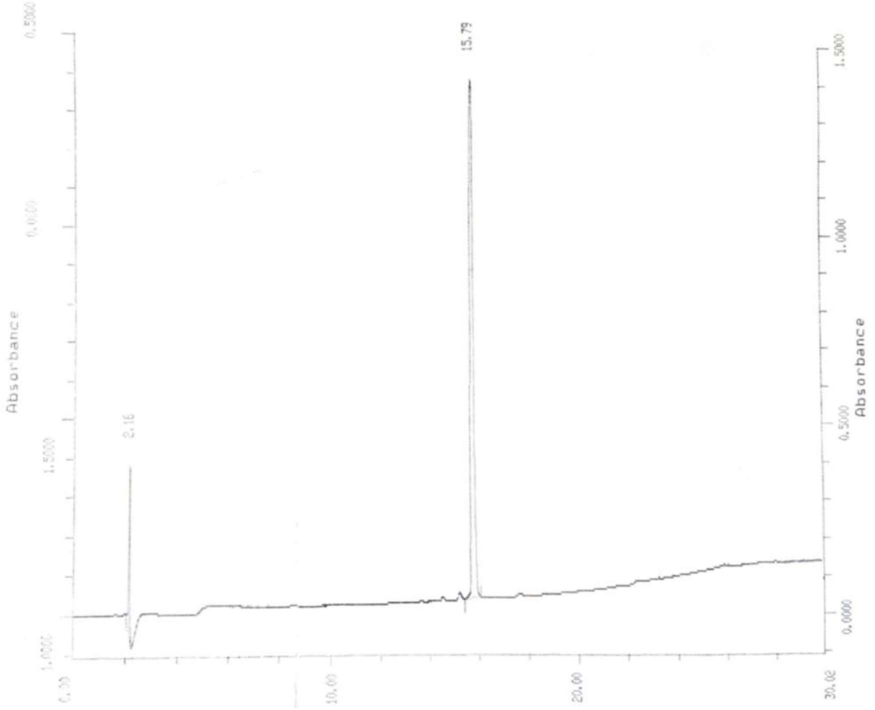

Compound H_4L2

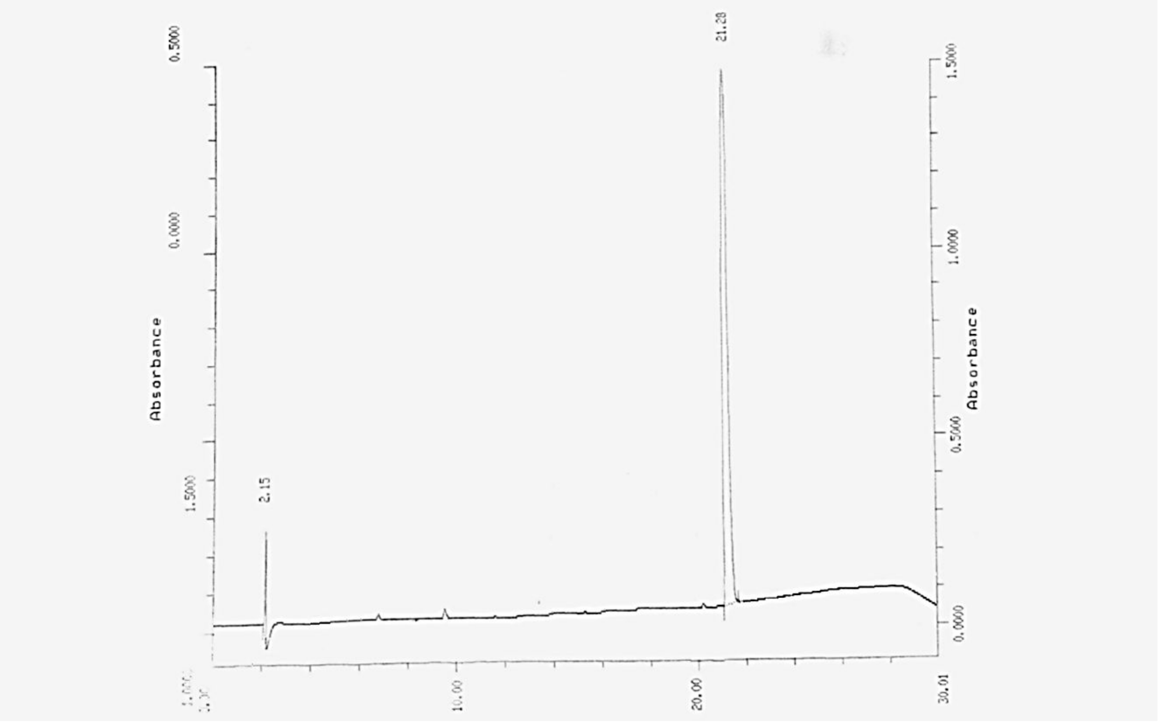

(1) Olshvang, E.; Szebesczyk, A.; Kozlowski, H.; Hadar, Y.; Gumienna-Kontecka, E.; Shanzer, A. Biomimetic ferrichrome: structural motifs for switching between narrow- and broad-spectrum activities in P. putida and E. coli. *Dalton Trans.* **2015,** *44* (48), 20850-20858.
(2) Schwarzenbach, G.; Schwarzenbach, K. Hydroxamatkomplexe. 1. Die stabilitat der eisen(iii)-komplexe einfacher hydroxamsauren und des ferrioxamins B. *Helv. Chim. Acta* **1963,** *46* (4), 1390-1399.
(3) Anderegg, G.; Leplatte.F; Schwarzenbach, G. Hydroxamatkomplexe. 3. Eisen(iii)-austausch zwischen sideraminen und komplexonen - diskussion der bildungskonstanten der hydroxamatkomplexe. *Helv. Chim. Acta* **1963,** *46* (4), 1409-1422.
(4) Szebesczyk, A.; Olshvang, E.; Besserglick, J.; Gumienna-Kontecka, E. Influence of structural elements on iron(III) chelating properties in a new series of amino acid-derived monohydroxamates. *Inorg. Chim. Acta* **2018,** *473*, 286-296.
(5) A.L. Crumbliss, Aqueous solution equilibrium and kinetic studies of iron siderophore and model siderophore complexes, in: G. Winkelmann (Ed.) Handbook of microbial iron chelates, CRC Press Inc., New York, **1991**, pp. 177.
(6) Toporivska, Y.; Gumienna-Kontecka, E. The solution thermodynamic stability of desferrioxamine B (DFO) with Zr(IV). *J. Inorg. Biochem.* **2019,** *198*, 110753-110758.
(7) Caudle, M. T.; Crumbliss, A. L. Dissociation kinetics of (n-methylacetohydroxamato) iron (iii) complexes - a model for probing electronic and structural effects in the dissociation of siderophore complexes. *Inorg. Chem.* **1994,** *33* (18), 4077-4085.
(8) Mular, A.; Shanzer, A.; Kozłowski, H.; Decristoforo, C.; Gumienna-Kontecka, E. *Unpublished results*.
(9) Zeglis, B. M.; Lewis, J. S. A practical guide to the construction of radiometallated bioconjugates for positron emission tomography. *Dalton Trans.* **2011,** *40* (23), 6168-6195.
(10) Sanchiz, J.; Esparza, P.; Dominguez, S.; Brito, F.; Mederos, A. Solution studies of complexes of iron(III) with iminodiacetic, alkyl-substituted iminodiacetic and nitrilotriacetic acids by potentiometry and cyclic voltammetry. *Inorg. Chim. Acta* **1999,** *291* (1-2), 158-165.
(11) Intorre, B. J.; Martell, A. E. Zirconium complexes in aqueous solution. 3. Estimation of formation constants. *Inorg. Chem.* **1964,** *3* (1), 81-87.
(12) Perera, W. N.; Hefter, G. Mononuclear cyano- and hydroxo-complexes of iron(III). *Inorg. Chem.* **2003,** *42* (19), 5917-5923.
(13) Brown, P.L.; Ekberg, C. Hydrolysis of Metal Ions, Willey, Vebrlag GmbH and Co. KGaA, **2016**.
(14) Baes, C. F.; Mesmer, R. E. The thermodynamics of cation hydrolysis. *Am. J. Sci.* **1981,** *281* (7), 935-962.
